# Direct synthesis of mycophenolic acid aryl esters with antioxidant and antiproliferative properties

**DOI:** 10.1038/s41598-025-11871-5

**Published:** 2025-07-17

**Authors:** Juliusz Walczak, Dorota Iwaszkiewicz-Grześ, Magdalena Śliwka-Kaszyńska, Agnieszka Kurdyn, Ewa Augustin, Agnieszka Viapiana, Alina Plenis, Grzegorz Cholewiński

**Affiliations:** 1 Department of Organic Chemistry, Faculty of Chemistry, Gdańsk University of Technology, ul. G. Narutowicza 11/12, Gdańsk, 80-233 Poland; 2 Laboratoire de Chimie des Polymères, Faculté des Sciences, Université Libre de Bruxelles (ULB), Boulevard du Triomphe, Bruxelles, 1050 Belgium; 3 Department of Medical Immunology, Faculty of Medicine, Medical University of Gdansk, ul. Dębinki 7, Gdańsk, 80-210 Poland; 4 Department of Pharmaceutical Technology and Biochemistry, Faculty of Chemistry, Gdańsk University of Technology, ul. G. Narutowicza 11/12, Gdańsk, 80-233 Poland; 5 Department of Analytical Chemistry, Faculty of Pharmacy, Medical University of Gdańsk, Al. Gen. J. Hallera 107, Gdańsk, 80-416 Poland

**Keywords:** Mycophenolic acid, Natural phenols, Esterification, IMPDH inhibition, Antioxidants, Medicinal chemistry, Organic chemistry

## Abstract

Naturally occurring phenols were incorporated into the mycophenolic acid (MPA) core to form sixteen new MPA derivatives. Ester conjugates of MPA with isoeugenol, methyl *o*-coumarate, and raspberry ketone exhibited the highest antioxidant activity in the DPPH test. These derivatives were then tested against the human pancreatic cancer AsPC-1 cells, showing cytotoxicity similar to that of the referential parent compounds MPA and mycophenolate mofetil (MMF). Subsequently, most of the obtained MPA esters proved activity against the T-Jurkat cells and peripheral blood mononuclear cells (PBMCs), serving as an immunosuppressive model, and worked as inosine-5’-monophosphate dehydrogenase (IMPDH) inhibitors. The most promising immunosuppressive conjugates were the esters of MPA with sesamol and raspberry ketone structural motifs, which held the highest selectivity index (SI) for PBMCs, thus serving as a good starting point for new drug development.

**Supplementary Information:**

The online version contains supplementary material available at 10.1038/s41598-025-11871-5.

## Introduction

Mycophenolic acid (MPA) is a natural compound^1,2^ that is an effective inhibitor of inosine-5’-monophosphate dehydrogenase (IMPDH), widely applied in clinics in the prophylaxis of allograft rejection and autoimmune disorders treatment, in the forms of its prodrugs: sodium mycophenolate (MPS) and mycophenolate mofetil (2-morfolinoethyl, MMF)^3–5^. Disruption of IMPDH in the biosynthesis of purine nucleotides, which are needed for lymphocyte T and B to proliferate correctly, modifies immunological response. Noteworthy, novel MPA derivatives are also investigated to improve therapeutic effectiveness and mitigate undesired side effects^6–9^. Nevertheless, the biological properties of MPA and its derivatives are not limited to immunosuppressive potential^10^. MPA inhibits both isoforms of the enzyme: IMPDH I, which is expressed mainly in normal cells, and IMPDH II being significant in neoplastic cells. The selectivity towards IMPDH II is one among many possible explanations for MPA and its derivatives to exhibit activity against cancer cells^11–13^. Moreover, these compounds also have antibacterial, antifungal, antiviral, and antiparasitic properties^14–20^.

One may find reported data concerning the antioxidant activity of MPA against reactive oxygen species (ROS) as well, which at least may be partially related to its anti-inflammatory or anticancer features^21–25^. Recently, the MPA-curcumin *O*-aryl ester conjugate was described as a promising antipsoriatic agent, possessing antiproliferative properties against tumor necrosis factor alpha (TNF-α), induced human keratinocyte cells (HaCaT)^26^. Furthermore, phenolic compounds and their derivatives were widely investigated as antioxidants bearing anticancer, anti-inflammatory, and antimicrobial activities^27–36^.

The primary objective of this study was the synthesis of novel ester derivatives of mycophenolic acid (MPA) with naturally occurring phenolic compounds possessing well-documented antioxidant properties, with the aim of evaluating their potential antioxidant and anticancer activities. This work represents an intentional shift away from the conventional immunosuppressive activity typically associated with MPA and its derivatives, by exploring alternative mechanisms of action. In particular, the study sought to identify compounds with promising antioxidant capabilities that may have relevance in the context of carcinogenesis. The antioxidant properties of the synthesized esters were assessed using the DPPH radical scavenging assay, while their cytotoxic effects were evaluated against the human pancreatic cancer cell line AsPC-1. Additionally, the antiproliferative activity of the resulting conjugates was preliminarily investigated using the T-Jurkat cell line, as a model for immunosuppression, and peripheral blood mononuclear cells (PBMCs), in order to monitor the retention or attenuation of immunosuppressive properties following structural modification.

**Fig. 1:**
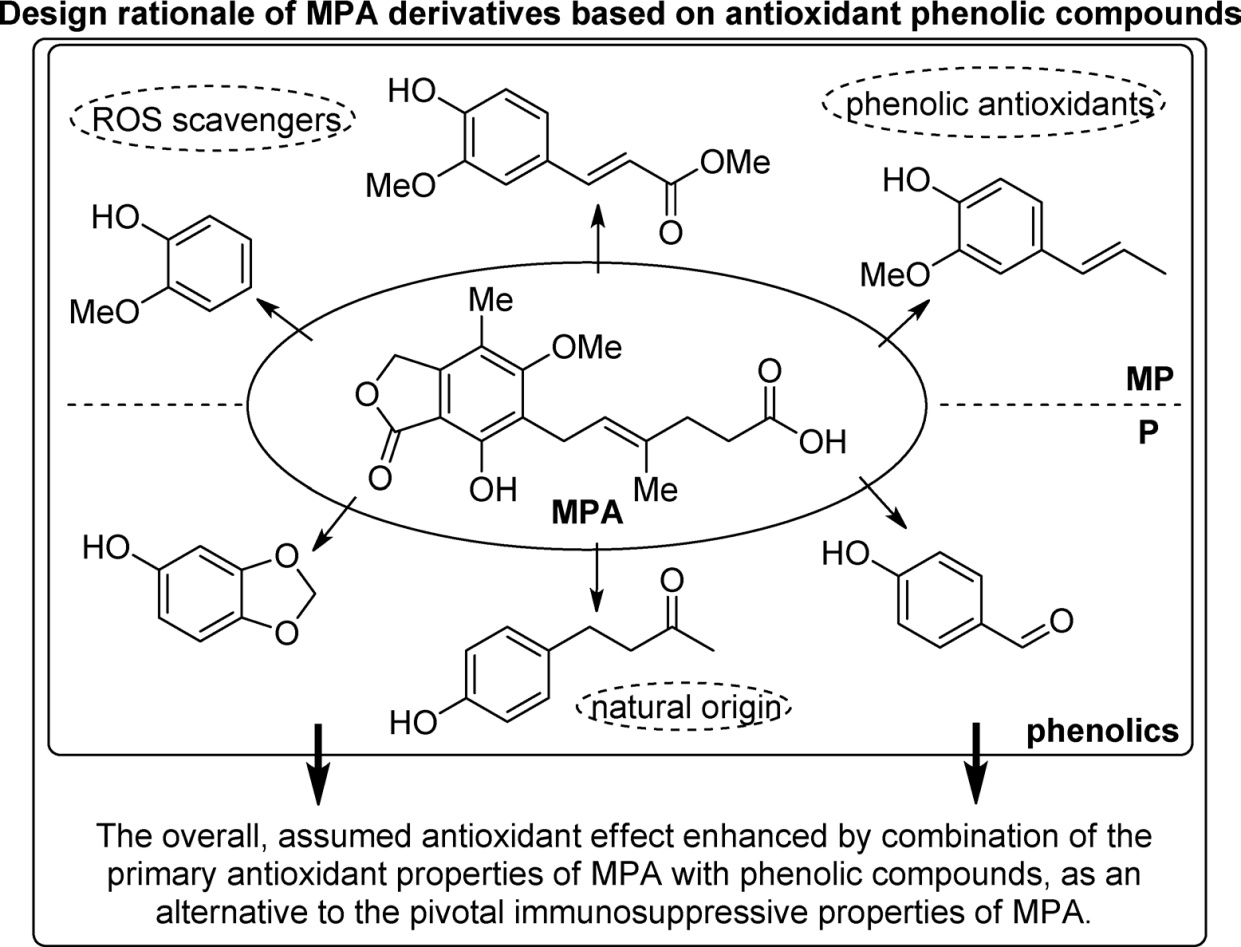
Selection of antioxidant phenolic compounds used for the synthesis of MPA derivatives.

To illustrate the rationale behind the compound selection, a simplified design scheme is presented in Fig. 1.

## Results and discussion

### Synthesis

**Fig. 2:**
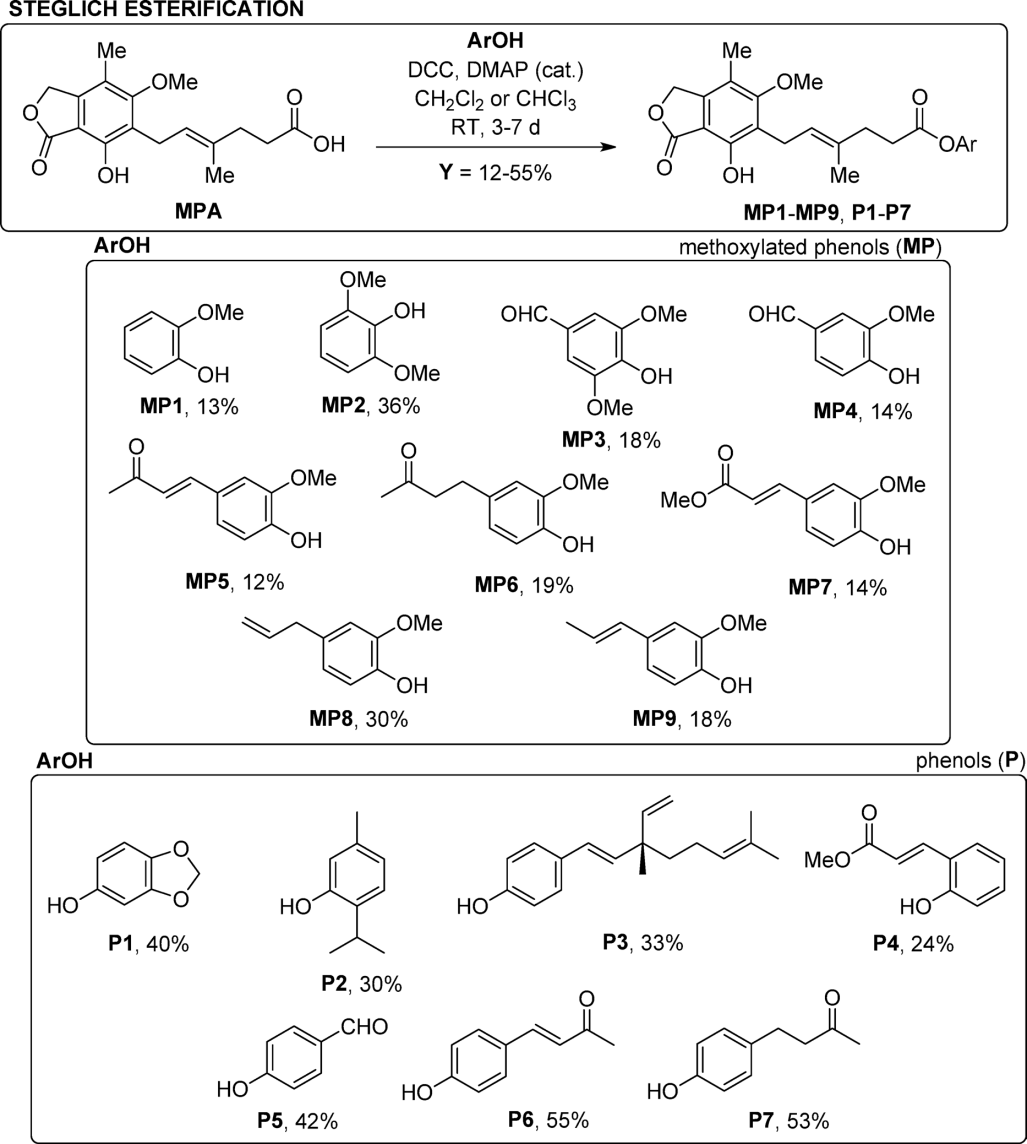
A collection of synthesized ester derivatives of MPA through the Steglich reaction.

Designed MPA conjugates include selected, naturally occurring phenolic moieties with various functional groups in their side chains, affecting their overall bioactivity. Their syntheses are shown in Fig. 2. The synthesized compounds fall into two subclasses, based on the type of attached phenolic unit: MP, *ortho*-methoxylated phenols (MP1 — guaiacol, MP2 — syringol, MP3 — syringic aldehyde, MP4 — vanillin, MP5 — dehydrozingerone, MP6 — zingerone, MP7 — methyl ferulate, MP8 — eugenol, MP9 — isoeugenol), and P, phenolics without *ortho*-methoxy substituent (P1 — sesamol, P2 — thymol, P3 — bakuchiol, P4 — methyl *o*-coumarate, P5 — *p*-hydroxybenzaldehyde, P6 — dehydroframbinone, P7 — frambinone, raspberry ketone).

Although the MPA molecule also possesses a phenolic group that can participate in undesired esterification, other substituents present in the aromatic ring rather impaired its nucleophilicity. Thus, we attempted to optimize the direct synthesis of designed ester derivatives of MPA without needing to protect its free phenolic group.

For this class of compounds, we used the Steglich esterification reaction protocol^37,38^as it requires easily accessible reagents and can be done straightforwardly under mild conditions. The Mukaiyama esterification methodology^39^ also provided us with a few expected esters with similar yields. However, due to the presence of a yellow-staining pyrid-2-one by-product, which was not easily removed chromatographically, and the lack of reaction for poorly nucleophilic phenols, we primarily used the first procedure. Furthermore, acid chloride-based methods (e.g. Yamaguchi esterification^40^) were not convenient and did not yield the expected target molecules.

In turn, one may observe reduced reaction outcomes due to the concomitant competition between various nucleophiles towards activated carboxylic acid moiety and ambiguous trends in phenolic group nucleophilic activity. When MPA functionalized with the *t*-butyldimethylsilyl (TBDMS) group on the free phenolic moiety was used, the yield of the Steglich reaction (with dehydrozingerone) was higher. Although the use of TBDMS protection improved the Steglich reaction yield, subsequent deprotection conditions promoted ester hydrolysis, which ultimately resulted in similar overall outcomes. Therefore, full optimization was beyond the scope of this initial screening study. Additionally, an example of obtaining MPA and curcumin ester can be found in the literature, and as those presented herein, its yield is suboptimal and equals 16%^26^.

It is not possible to determine a clear trend in the reactivity of these phenols with the activated carboxyl function of MPA, but some correlation can be observed, although it may be burdened with a large error. In the group of methoxylated phenols (MP), adding a methoxy group increases the yield of the resulting products (MP1 — guaiacol, 13% vs. MP2 — syringol, 36%). However, the introduction of additional functionalities, especially electron-withdrawing groups, such as aldehyde (MP3 — syringaldehyde, 18%) or carboxylic ester (MP7 — methyl ferulate, 14%), leads to a significant drop in yield. Similarly, MP4 (vanillin, 14%) and MP5 (dehydrozingerone, 12%) follow this trend, indicating that the presence of an aldehyde function can have a detrimental effect on the reactivity, possibly due to electron-withdrawing properties and/or competitive reactivity.

The influence of steric hindrance is also notable, especially in derivatives with longer side chain, such as MP5 and MP6 (dehydrozingerone and zingerone, 12 and 19%), where decreased yields may stem from reduced accessibility of the phenolic hydroxyl group. Interestingly, eugenol (MP8, 30%) and isoeugenol (MP9, 18%) showed moderate reactivity, with the geometry of the side chain (allyl vs. propenyl) possibly contributing to this difference.

In the class of non-methoxylated phenolics (P series), the highest yields were obtained for dehydroframbinone (P6, 55%) and raspberry ketone (P7, 53%), suggesting that certain ketone-substituted structures favor esterification under Steglich conditions. Sesamol (P1, 40%) and 4-hydroxybenzaldehyde (P5, 42%) also showed good reactivity, potentially due to their relatively small size.

Conversely, some structures like *o*-coumaric acid methyl ester-derived ester (P4, 24%) showed moderate yields, possibly due to conjugation effects and reduced nucleophilicity of the phenolic OH. Thymol (P2, 30%) and bakuchiol (P3, 33%) provided moderate yields, which may result from a balance of steric and electronic effects.

### DDPH test

Antioxidative properties of the obtained MPA derivatives were assessed chemically using a colorimetric DPPH test. The results, given as IC_50_ values, for MP1-MP9, P1-P7, and some reference compounds, are presented in Table 1. The IC_50_ value represents the scavenging activity, defined as the concentration of a compound required to scavenge 50% of DPPH radicals.

**Table 1:** DPPH test results for ester derivatives of MPA and selected references.

Type	Sample name	IC_50_ [mM]
Ester derivatives	MP1	23.788 ± 0.411^i^
	MP2	3.724 ± 0.230^b^
	MP3	12.239 ± 0.252^e^
	MP4	6.403 ± 0.311^c^
	MP5	(↓)
	MP6	14.902 ± 0.340^b, c^
	MP7	3.643 ± 0.113^b^
	MP8	6.902 ± 0.225^c^
	MP9	0.373 ± 0.041^a^
	P1	43.570 ± 0.380^l^
	P2	11.667 ± 0.113^d^
	P3	20.100 ± 0.258^h^
	P4	0.308 ± 0.031^a^
	P5	28.225 ± 0.357^j^
	P6	35.844 ± 0.403^k^
	P7	0.124 ± 0.000^a^
References	MPA	15.733 ± 0.144^f^
	MMF	18.962 ± 0.279^g^
	RES	0.131 ± 0.000^a^
	Trolox	0.008 ± 0.000^a^
	Ascorbic acid	0.545 ± 0.000^a^

Means followed by the same letter (a-l) within the same column indicate no significant difference among samples according to Tukey’s HSD test (*p* < 0.05); (↓)—precipitating compound. Data is represented as mean ± standard deviation.

The synthesized compounds were able to reduce the stable radical DPPH in a concentration-dependent manner. Results were expressed as IC_50_ and all synthesized compounds exhibited measurable DPPH scavenging activity in the sub-millimolar range, although clearly lower than that of the reference antioxidants Trolox (IC_50_ = 0.008 mM) and ascorbic acid (IC_50_ = 0.545 mM). Additionally, resveratrol (RES) (IC_50_ = 0.131 mM) was used as a supplemental reference due to its well-known antioxidative properties^41,42^. Mycophenolic acid (MPA) (IC_50_ = 15.733 mM), and mofetil mycophenolate (MMF) (IC_50_ = 18.962 mM) served as constitutive antioxidative models for this class of compounds. In DPPH scavenging activity the most active esters were: P7 (IC_50_ = 0.124 mM), followed by P4 (IC_50_ = 0.308 mM), and MP9 (IC_50_ = 0.373 mM), overcoming referential MPA and MMF values. Moreover, these conjugates (P7, P4, and MP9) revealed higher activity than ascorbic acid, a well-known free radical scavenger involved in many biochemical processes related to oxidative stress^42,43^. Other samples displayed a medium range of DPPH scavenging activity.

The structure-activity relationship is, however, impossible to define as all of these compounds consist of structural patterns responsible for free radical stabilization, such as isolated or conjugated unsaturated systems (aromatic or non-aromatic), free phenolic group, aldehyde group, as well as methoxyl groups (electron donating groups in general) attached to an aromatic system^44,45^and none of them could unambiguously dominate over the other.

Among the most potent antioxidants (lowest IC_50_ values), MP9 (0.373 mM), P4 (0.308 mM), and P7 (0.124 mM) stand out. MP9 (derived from isoeugenol) and P4 (from methyl *o*-coumarate) feature conjugated systems, promoting resonance stabilization of possible, resulting radical species. Interestingly, P7 (based on raspberry ketone), despite lacking extended π-conjugation beyond the aromatic ring, shows excellent activity — possibly due to the favorable electronic effects of the carbonyl group.

On the other hand, several compounds exhibit weak antioxidant activity despite possessing multiple unsaturated bonds. P3 (from bakuchiol), for example, displays relatively poor activity (20.100 mM). Similarly, P6 (from dehydroframbinone), with its conjugated system, showed weak activity (35.844 mM), emphasizing that extensive unsaturation alone does not guarantee effective radical scavenging. Compound P1 (from sesamol) showed relatively weak antioxidant activity (43.570 mM). As the phenolic hydroxyl group is esterified, direct redox reactivity through classical phenolic mechanisms is not available. The benzodioxole system contributes the –I inductive effect and, despite possessing potential + M resonance-donating capacity, is not conjugated in a way that would meaningfully stabilize a radical intermediate. This likely accounts for the compound’s low efficacy in the DPPH assay.

While all compounds in this study are esters of MPA, their antioxidant activity — measured via the DPPH assay — varies noticeably depending on the structural characteristics of the phenolic moiety introduced. The redox behavior appears to be primarily governed by the nature of the aromatic fragment, with IC_50_ values ranging from highly active derivatives like MP2 (3.724 mM) and MP7 (3.643 mM) to weakly active ones such as MP1 (23.788 mM). For comparison, MPA itself exhibited an IC_50_ of 15.733 mM. This suggests that some esterified derivatives can either enhance or suppress antioxidant properties relative to the parent acid, depending on how the introduced substituents influence electron distribution, conjugation, and steric accessibility.

These findings confirm that certain phenolic structures can retain or even enhance antioxidant potential after esterification with MPA, supporting their further exploration as multifunctional bioactive compounds.

In the group of the most potent antioxidants, namely: MP9, P4, and P7, the first two possess additional unsaturated bonds and a functional group affecting the aromatic ring through electronic effects. The last one is lacking any conjugated, additionally stabilizing systems, merely having a carbonyl group in its side chain. In contrast, a notable example of the absence of antioxidant activity despite the presence of numerous isolated double bonds is observed in P3. The sole aromatic ring in P1 did not improve the IC_50_ value thus implying its minor role in independent radical stabilization for this class of compounds. It shows quite a complex character of redox properties exhibited by such multifunctional compounds, being the result of many structural and energetic factors. Although no dedicated hydrolytic stability study was performed, the esters were consistently handled in the presence of air, water-containing solvents, and during chromatographic and spectroscopic procedures without any signs of degradation. This suggests a reasonable degree of stability under the conditions used.

### Biological evaluation

Serious side effects may be observed with the intake of various drugs, including the well-known and clinically applied MPA^4–6^. Thus, the most common approach in medicinal chemistry is to assess the cytotoxicity and effectiveness of newly designed substances, to better understand their mechanisms of action, and to more reasonably manage the balance between beneficial and adverse properties.

Building on the previous chemical DPPH test results, the MTT test was conducted on the AsPC-1 cell line for the most potent antioxidants (namely: MP9, P4, P7) to evaluate their cytotoxic activity. The outcomes are summarized in Table 2 and ESI Fig. S1 (see electronic supplementary information, p. 2).

The XTT test was performed to assess toxicity towards the T-Jurkat cell line and PBMCs, with the results presented in Table 3 and ESI Fig. S2 (see electronic supplementary information, p. 2). Ester derivatives of MPA are likely insoluble in aqueous media, so IC_50_ values for some derivatives were not collected. The subsequent biological evaluation included proliferation tests, using the VPD540 staining assay, where the EC_50_ values for T-Jurkat and PBMCs were obtained. These results are summarized in Table 4 and depicted in a graph for the most potent derivatives (ESI Fig. S3, see electronic supplementary information, p. 3). Selectivity Index (SI) values, representing the ratio between the toxic concentration (IC_50_) and effective concentration (EC_50_), relative to MPA or MMF, are shown in Table 5. Given that the primary properties of MPA and MMF involve immunosuppressive activity through IMPDH enzyme inhibition, GMP tests (concerning PBMCs) for MPA ester derivatives were conducted. The results for the most potent derivers are summarized in ESI Fig. S4 (see electronic supplementary information, p. 4).

Given that oxidative stress and redox imbalance can be significant risk factors for the development of pancreatic cancer, we selected the AsPC-1 cell line as a model of ROS-dependent pancreatic cancer to test the most potent antioxidants (as identified by the DPPH assay).

To complement the antioxidant evaluation and monitor the broader biological relevance of the synthesized derivatives, two additional cell models were included. The T-Jurkat line, derived from human T lymphocytes, was used to monitor potential cytotoxic or immunomodulatory effects, given the known antiproliferative activity of MPA. PBMCs from healthy donors served as a standard non-cancerous control. GMP tests were conducted on healthy cells (PBMCs) for the most potent esters of MPA, as well as on AsPC-1 and T-Jurkat cell lines, to determine whether these compounds act as IMPDH inhibitors, thus allowing us to explore both the novel and typical bioactivities of MPA derivatives. This setup, routinely used in our group^7,9,46–48^provides internal verification of how far the synthesized derivatives diverge from MPA’s original immunosuppressive profile, in line with the intended shift toward redox-based activity.

### Assessment of cytotoxic activity using the MTT method on the AsPC-1 cell line

Table 2 presents the concentration of the tested compounds, namely: P4, P7, MP9, MPA, MMF, and gemcitabine (GEM; a standard), which inhibited the proliferation of AsPC-1 cells (human pancreatic cancer cells) by 50% (expressed as IC_50_ values) compared to the control. Cells were treated with compounds for 72 h and then a colorimetric MTT assay was performed. A graphical representation of these values can be seen in ESI Fig. S1.

**Table 2:** Cytotoxicity of the most promising antioxidants and gemcitabine (as a standard) against AsPC-1 cells expressed as IC_50_ values (*n* = 3).

AsPC-1 cell line	
Symbol	IC_50_ [µM]
P4	2.867** ± 0.049
P7	2.453** ± 0.092
MP9	3.812** ± 0.517
MPA	1.740 ± 0.259
MMF	2.029 ± 0.042
GEM	0.025*** ± 0.001

Significance was calculated using the two-tailed unpaired T-test; results are considered statistically significant according to the following criteria: *(*p* < 0.05), **(*p* < 0.01), and ***(*p* < 0.001), with MPA as a control. Data is represented as mean ± standard deviation.

Results presented above indicate that all studied compounds exhibit cytotoxic properties towards the AsPC-1 cells in the same order of magnitude as MPA and MMF. However, gemcitabine (a standard) is still a more potent chemotherapeutic for pancreatic cancer cells, though these studies were not carried out to overtake its biological properties, but to find other applications for MPA and its derivatives, based on redox processes. Therefore, the AsPC-1 cell line was selected for this evaluation due to its reactive oxygen species-related properties and thus free radicals sensibility^49,50^.

Upon this research, it cannot be concluded that the presence of free radicals’ stabilizing moieties (e.g. conjugated double bonds) translates directly into cytotoxic activity towards pancreatic cancer cells, where oxidative stress plays a crucial role in pathogenesis mechanisms. Although raspberry ketone itself has proven antioxidant properties^51^its esters may not possess them, due to the loss of the phenolic moiety, which is important from the perspective of radical processes. In the case of the P7 molecule, the presence of the MPA core most likely influences this property.

Additionally, redox processes occurring in cells, including the aforementioned oxidative stress, involve an imbalance between the production of free radicals and the mechanisms of their scavenging^50^. Therefore, the presence of potentially pro-oxidative ketone groups (referencing quinone systems^44^) does not exclude their importance in these processes. The slightly higher IC_50_ values ​​for P4 (methyl *o*-coumarate derivative, 2.867 µM) and MP9 (isoeugenol derivative, 3.812 µM) compared to P7 (raspberry ketone derivative, 2.453 µM) further support this reasoning.

Noteworthy, some adverse effects caused by MPA can be at least partially due to its oxidative stress promotion^52^. Therefore, although no correlation between DPPH antioxidant and cytotoxic activities of investigated MPA conjugates against AsPC-1 cells were observed, these compounds may provide chemotherapeutics with reduced toxicity similar to raspberry ketones, which were investigated as ROS inhibitors in cyclophosphamide therapies^28^.

### Determination of cytotoxic activity using the XTT method on the T-Jurkat cell line

Table 3 presents the IC_50_ values obtained from the XTT test on PBMCs and the T-Jurkat cell line, using MPA and MMF as standards. These values indicate the concentration of each compound required to reduce the viability of PBMCs (healthy cells) and T-Jurkat cells (T-cell leukemia) by 50% compared to the control. Cells were treated with compounds for 72 h (PBMCs) or 48 h (T-Jurkat), followed by a colorimetric XTT test. The results are illustrated in ESI Fig. S2 as a viability curve in relation to concentration.

**Table 3:** Cytotoxicity of MPA ester derivatives on PBMC and T-Jurkat cells.

Entry	Relative IC_50_ [µM]		IS [–]
	PBMCs	T-Jurkat	
MPA	18.732 ± 0.030	0.527 ± 0.040	35.558
MMF	16.613 ± 0.041	0.740 ± 0.034	22.456
MP1	14.782 ± 0.016	0.982 ± 0.005	15.051
MP2	22.519 ± 0.039	1.748 ± 0.006	12.883
MP3	10.588 ± 0.040	4.443 ± 0.007	2.383
MP4	23.484 ± 0.015	5.330 ± 0.005	4.406
MP5	30.698 ± 0.024	2.325 ± 0.004	13.203
MP6	14.715 ± 0.045	**–**	**–**
MP7	30.433 ± 0.044	1.406 ± 0.004	21.645
MP8	17.782 ± 0.011	1.705 ± 0.002	10.429
MP9	21.153 ± 0.015	1.964 ± 0.002	10.770
P1	125.475 ± 0.025	1.073 ± 0.002	116.938
P2	16.349 ± 0.010	2.473 ± 0.004	6.611
P3	14.117 ± 0.007	**–**	**–**
P4	25.887 ± 0.017	1.658 ± 0.003	15.613
P5	9.948 ± 0.046	1.582 ± 0.003	6.288
P6	8.836 ± 0.020	**–**	**–**
P7	50.294 ± 0.019	2.410 ± 0.002	20.869

IS—internal selectivity; defined as a ratio between IC_50_ values for PBMCs and T-Jurkat cell line. Data is represented as mean ± standard deviation.

Regarding the XTT test for PBMCs, it can be observed that nearly half of the tested esters are more cytotoxic towards healthy cells than the referential MPA and MMF. All of the esters are less cytotoxic on T-Jurkat cells, typically being an order of magnitude less potent for most cases, with MP1 (the guaiacol derivative) showing comparable values to the standards. Notably, reduced cytotoxicity towards the T-Jurkat cell line was observed for MP4, MP3, P2, P7, and MP5, most of which contain a ketone/aldehyde group within its structure. However, due to the low IC_50_ value for P5, no definitive conclusion can be drawn about the importance of this moiety in disrupting immunosuppressive properties.

A high IC_50_ value for PBMCs combined with a low IC_50_ value for the T-Jurkat cell line may indicate the selectivity of ester derivatives towards leukemic cells. Such compounds are characterized by low toxicity towards healthy cells and high toxicity towards diseased cells. Significantly high values of the internal selectivity factor IS can be observed for P1, MP7, P7, P4, and MP1, indicating their potential as immunosuppressants.

Taking into account the differentiation of the activity of compounds depending on their structure, certain SAR-type relationships can be observed. Compounds containing carbonyl groups, such as MP4 or MP3, show reduced cytotoxicity towards T-Jurkat cells, which may indicate the influence of these groups on the selectivity or permeability of cell membranes.

Considering the variation in activity depending on the compounds’ structure, certain SAR trends can be observed. Compounds containing carbonyl groups, such as MP4 or MP3, show reduced cytotoxicity against T-Jurkat cells, which may suggest that these moieties influence selectivity or membrane permeability. In contrast, MP1, a guaiacol derivative, displays high activity and a favorable selectivity index, suggesting that the presence of methoxy groups may enhance affinity toward immune-related targets or improve the bioavailability of the compound. Analysis of these features may serve as a starting point for designing further analogs with an improved activity profile.

Interestingly, the influence of methoxy-related motifs can be further explored by comparing MP1 (a guaiacol derivative), MP2 (a syringol derivative), and P1, which contains a benzodioxole moiety. While MP1 shows favorable cytotoxicity and selectivity, MP2 — with two methoxy groups — exhibits reduced selectivity, suggesting that increased methoxylation does not necessarily enhance activity. Notably, P1 demonstrates extremely high selectivity (IS = 116.938), despite relatively low cytotoxicity toward leukemic cells. This may be attributed to the structural rigidity and electronic characteristics of the benzodioxole ring, which differs significantly from simple methoxy substitutions. These findings highlight that not only the presence, but also the structural context of electron-donating groups can greatly influence biological activity, warranting further SAR exploration.

### Determination of antiproliferative activity using VPD450 in flow cytometry

Table 4 displays the EC_50_ values obtained from the VPD450 test on PBMCs and the T-Jurkat cell line, with MPA and MMF as reference standards. These values indicate the concentration of any compound at which proliferation of PBMCs (healthy cells), and T-Jurkat cells (T-cell leukemia), is inhibited by 50% compared to the control. Cells were treated with the compounds for 72 h (PBMCs) or 48 h (T-Jurkat), followed by a colorimetric VPD450 test. The results are presented in ESI Fig. S3 as proliferation curves concerning concentration.

**Table 4:** Antiproliferative activity of MPA ester derivatives on PMBC and T-Jurkat cells.

Entry	EC_50_ [µM]		IS [–]
	PBMCs	T-Jurkat	
MPA	1.154 ± 0.006	0.924 ± 0.034	1.249
MMF	1.037 ± 0.006	0.609 ± 0.030	1.704
MP1	1.106 ± 0.004	0.780 ± 0.034	1.418
MP2	5.395 ± 0.006	2.642 ± 0.035	2.042
MP3	13.742 ± 0.005	4.458 ± 0.040	3.083
MP4	2.900 ± 0.005	7.509 ± 0.036	0.386
MP5	11.406 ± 0.003	1.082 ± 0.036	10.542
MP6	1.162 ± 0.003	0.662 ± 0.031	1.755
MP7	7.734 ± 0.007	4.923 ± 0.034	1.571
MP8	1.154 ± 0.003	1.735 ± 0.023	0.665
MP9	2.356 ± 0.003	1.836 ± 0.043	1.283
P1	1.152 ± 0.004	2.012 ± 0.042	0.573
P2	1.417 ± 0.003	4.477 ± 0.033	0.317
P3	1.052 ± 0.004	1.340 ± 0.035	0.780
P4	4.865 ± 0.004	3.526 ± 0.045	1.380
P5	1.656 ± 0.003	2.357 ± 0.023	0.703
P6	2.215 ± 0.004	2.840 ± 0.033	0.780
P7	1.047 ± 0.006	2.226 ± 0.044	0.470

*IS* internal selectivity; defined as a ratio between EC_50_ values for PBMCs and T-Jurkat cell line. Data is represented as mean ± standard deviation.

As can be observed in Table 4 the vast majority of compounds have EC_50_ values higher than those of MPA or MMF for both PBMCs and T-Jurkat cells. However, a few ester derivatives show high efficacy in inhibiting T-Jurkat cell proliferation, specifically: MP6 (0.662 µM; lower than MPA and close to MMF), MP1 (0.780 µM; lower than MPA) and MP5 (1.082 µM; close to MPA). Six out of sixteen derivatives (P7, P3, MP1, P1, MP8, MP6) demonstrate similar toxicity towards PBMCs as MPA or MMF, consistent with the trend previously seen for IC_50_ values. Most compounds exhibit EC_50_ values an order of magnitude higher than referential MPA and MMF.

The highest internal selectivity factor values in MP5, MP3, MP2, MP6, and MP7, suggest a stronger efficacy in targeting the T-Jurkat cell line while exhibiting reduced antiproliferative effects on PBMCs. This trend contrasts with the IS values assessed for IC_50_ values, which are notably lower for the P class of derivatives (P1-P7), indicating a more pronounced cytotoxic character.

A closer inspection of the EC₅₀ and IS values reveals emerging structure–activity relationships within the ester derivatives. Notably, several derivatives displaying high internal selectivity factors—such as MP5, MP3, MP6, and MP7—share structural features involving carbonyl-containing side chains (aldehyde or ketone groups), which may modulate cellular uptake or enhance specific interactions with intracellular targets involved in cell proliferation. This observation is consistent with the pattern seen in the XTT assay and suggests that such moieties could play a dual role in influencing both cytotoxic and antiproliferative properties.

Interestingly, a divergence in selectivity patterns is observed between the MP and P compound series. While P-class derivatives (P1–P7) generally exhibited higher IS values in the XTT assay, indicating cytotoxic selectivity, the MP-series compounds—particularly MP5, MP6, and MP7—demonstrate greater selectivity in the VPD450 antiproliferative test. This suggests that the P-derivatives exert more direct cytotoxic effects, whereas selected MP-esters are more effective in inhibiting proliferative pathways.

### Selectivity index determination

In this subsection, Selectivity Index (SI) values are presented. It is collected in Table 5. This factor is important in interpolating between cytotoxicity and efficacy of tested compounds. The higher the SI values, the lower the risk of cytotoxicity towards healthy cells (here: PBMCs), and the greater the potency against cancer cells (here: T-Jurkat).

**Table 5:** Selectivity index calculated for inter-series cytotoxicity and efficacy values.

Entry	SI [–]	
	PBMCs	T-Jurkat
MPA	16.232	0.570
MMF	16.020	1.216
MP1	13.365	**1.259**
MP2	4.174	0.662
MP3	0.770	0.997
MP4	8.098	0.710
MP5	2.691	**2.149**
MP6	12.664	–
MP7	3.935	0.286
MP8	**15.409**	0.983
MP9	8.978	1.070
P1	**108.919**	0.533
P2	11.538	0.552
P3	13.419	–
P4	5.321	0.470
P5	6.007	0.671
P6	3.989	–
P7	**48.036**	**1.083**

*SI* selectivity index; external selectivity; defined as the ratio between IC_50_ values and EC_50_ values for experiments conducted on the PMBCs or the T-Jurkat cell line.

P1, P7, and MP8 exhibit strongly pronounced SI values for PMBCs. Conversely, the SI values for the T-Jurkat cell line are not as distinctively elevated. Nevertheless, MP5, MP1, and P7 show noticeably high SI values compared to MPA and MMF.

The raspberry ketone structural motif appears to influence bioactivity, with P7 standing out as a derivative that shows reduced cytotoxicity towards healthy cells while maintaining good antiproliferative activity against T-Jurkat cells.

SAR analysis of the SI data reveals that specific functional groups may influence the selectivity between cytotoxic and antiproliferative actions. Notably, derivatives such as P1 and P7 (e.g. benzenedioxol or raspberry ketone motifs), exhibit high SI values for PBMCs, suggesting a capacity to suppress leukemic cell proliferation with minimal harm to healthy cells. In contrast, compounds like MP3 and MP4 show low SI values in T-Jurkat cells, with MP4 displaying a significantly higher SI value for PBMCs, indicating varying selectivity profiles between efficacy and cytotoxicity. MP5, on the other hand, stands out with a relatively high SI in T-Jurkat cells analysis, suggesting that certain ketone-bearing side chains may improve selectivity by enhancing antiproliferative activity while minimizing toxicity. These trends imply that both electronic effects and steric properties of side chains contribute to the therapeutic index and should be further optimized in future derivatives.

### Enzyme inhibition in the presence of guanosine source

ESI Fig. S4 illustrates the concentration-dependent inhibition of PBMC proliferation by MPA, MMF, and their derivatives, both in the presence and absence of 50 µM GMP. A strong inhibition of proliferation is observed at higher compound concentrations (50 − 10 µM), which diminishes with lower concentrations (1-0.1 µM). Notably, for all compounds, co-incubation with GMP substantially rescues proliferation levels, indicating that the inhibition is reversible and dependent on IMPDH blockade^53^.

MPA and MMF, known IMPDH inhibitors, show near-complete inhibition at 50 µM, with proliferation restored close to 100% upon GMP addition, in the MPA case. A similar trend is observed for the derivatives P1, P4, P7, and MP9. For example, P1 inhibits proliferation by ca. 80% at 50 µM, and this effect is reversed in the presence of GMP. P4, P7 and MP9 show a comparable pattern, indicating that their mechanism of action aligns with classical IMPDH inhibition.

The consistent reversal of proliferation inhibition in the presence of GMP across all tested derivatives supports the hypothesis that these compounds act via the IMPDH pathway, similarly to MPA and MMF. This observation strengthens the conclusion that the primary immunosuppressive mechanism of these compounds involves IMPDH inhibition, despite additional antioxidant properties.

## Conclusions

In this study, we synthesized and biologically evaluated a new series of sixteen ester derivatives of MPA, exploring their potential in terms of antioxidative properties, cytotoxicity, and antiproliferative efficacy. The Steglich reaction facilitated synthesis of these derivatives, which were obtained without the need for protective groups on the free phenolic group of MPA. The DPPH test demonstrated that three of these esters (P7, P4, and MP9) exhibited superior antioxidant potential compared to ascorbic acid, with ten compounds showing improved antioxidative activity compared to the parent MPA and MMF.

Based on their antioxidant potential, the most promising derivatives (P7, P4, and MP9) were further evaluated for cytotoxicity using the AsPC-1 cell line, a model for free radical-sensitive cells. These derivatives exhibited cytotoxic activity comparable to MPA and MMF, although they did not surpass gemcitabine, a well-established chemotherapeutic agent for pancreatic cancer. The compounds displayed comparable potency to the parent MPA, suggesting that their antioxidative properties could be leveraged for therapeutic applications, particularly in redox-related pathways.

In the cytotoxicity and antiproliferative assays using PBMCs and the T-Jurkat cell line, several derivatives exhibited increased cytotoxicity compared to MPA and MMF, with MP1 and P1 showing IC_50_ values near those of the references. The analysis of the internal selectivity (IS) revealed that P1 demonstrated significant selectivity toward T-Jurkat cells, suggesting potential immunosuppressive properties. Additionally, compounds MP6, MP1, and MP5 maintained high efficacy against T-Jurkat cells, with MP6 surpassing MPA in terms of EC_50_ values. Notably, MP5 exhibited a particularly high IS value, highlighting its strong selective activity against T-Jurkat cells with minimal toxicity toward PBMCs.

The Selectivity Index (SI), an important parameter for determining the suitability of compounds for further biological research, was also assessed. P1 and P7 exhibited high SI values when tested against PBMCs, while MP5, MP1, and P7 showed strong SI values against T-Jurkat cells. This suggests that these compounds exhibit favorable profiles for potential therapeutic development, with high selectivity for leukemic cells and reduced toxicity toward healthy cells.

Finally, the IMPDH inhibition test revealed that derivatives P7, P4, MP9, and P1 acted similarly to MPA and MMF, inhibiting PBMC proliferation in the presence of GMP. This confirms that these compounds share the same mechanism of action as MPA and MMF, primarily involving IMPDH inhibition.

In conclusion, the novel MPA derivatives presented in this study demonstrate promising biological activity with respect to antioxidation, cytotoxicity, and immunosuppressive properties. Derivatives P1 and P7, in particular, stand out due to their high selectivity for T-Jurkat cells, suggesting they may have significant potential for future therapeutic applications. These compounds function as IMPDH inhibitors, with P1 and P7 showing the most favorable profiles in terms of immunosuppressive properties and selectivity, making them suitable candidates for further optimization and development.

## Methods

### General

Substrates for synthesis (MPA, phenolics, 4-(*N*,*N*-dimethylamino)pyridine (DMAP), *N*,*N*’-dicyclohexylcarbodiimide (DCC) were acquired from Sigma-Aldrich, Apollo Scientific, Chemat, or Alfa Aesar. Solvents were dried and distilled using standard procedures. Thin layer chromatography (TLC) analyses were performed on Supelco silica gel TLC aluminium foils with fluorescence indicator 254 nm and visualized with UV light (254 nm) or iodine staining. Column chromatography was carried out on silica gel 60 (230–450 Mesh, Alfa Aesar).

1D and 2D NMR spectra were documented using the Varian Unity Inova 500 spectrometer (500 MHz for ^1^H NMR and 126 MHz for ^13^C NMR assays). Chemical shifts (δ) are demonstrated in parts per million (ppm), in accordance with tetramethylsilane (TMS), and coupling constants (J) are shown in hertz (Hz). The residual chloroform peak serves as the internal standard (7.26 ppm for ^1^H NMR and 77.16 ppm for ^13^C NMR).

Agilent liquid chromatograph series 1290 was used for HPLC-based purity assessment. The samples (2 µL) were injected into a column thermostated at 40 °C packed with the Poroshell EC-C18 2.7 μm (3.0 mm × 150 mm). The mobile phase flow rate was 0.4 mL∙min^− 1^, and elution was performed using 10 mM ammonium formate in water (solvent A) and in MeCN: MeOH (1:1, v/v) (solvent B) in gradient mode: from 50% B to 100% B in 25 min. The UV signals were registered at 254 and 305 nm.

The accurate mass of the samples was determined by electrospray ionization quadrupole time-of-flight mass spectrometry (ESI(-)-QTOF) analysis using the Agilent 1290 LC system coupled with the Agilent QTOF mass spectrometer G6546A (Santa Clara, CA, USA) operating in negative ionization scan mode (*m/z*, 50-1000). The nebulizer pressure, nitrogen flow rate, drying gas temperature, drying gas flow rate, and sheath gas temperature were as given: 45 psi, 5 L∙min^− 1^, 300 °C, 11 L min^− 1^, and 250 °C. The capillary voltage was 3.5 kV and the fragmentation voltages were 150 and 200 V.

Summarized data (R_F_, eluent, NMR, HPLC, HR-MS) for MPA, MMF and ester derivatives is presented in the Electronic Supplementary Information (ESI).

### General method for MP1-MP9 and P1-P7 synthesis

In an oven-dried, round bottom flask were placed 200 mg of mycophenolic acid (MPA, 0.624 mmol, 1.00 eq) and 81 mg of guaiacol (phenolics, 74 µL, 0.656 mmol, 1.05 eq). Substrates were dissolved in 4.0 mL of dry dichloromethane (DCM) or chloroform (C). Then, 8 mg of 4-(*N*,*N*-dimethylamino)pyridine (DMAP, 0.062 mmol, 0.10 eq) was added. The reaction mixture was cooled down to 0^o^C and 155 mg of *N*,*N*’-dicyclohexylcarbodiimide (DCC, 0.749 mmol, 1.20 eq) was put in one portion. This temperature was maintained for about 5 min and then the reaction was stirred for at least 72 h at room temperature. Excessive evaporation of the solvent may be hampered by underlying the flask with a vessel filled with water at room temperature. The reaction mixture was then diluted with at least 5.0 mL DCM, and extracted using NaHCO_3_ (sat.), 1 M HCl, and NaCl (sat.). The organic phase was dried over anhydrous magnesium sulfate or sodium sulfate, filtered, and concentrated under reduced pressure. The raw mixture was then purified on column chromatography using petroleum ether: ethyl acetate 3:1 (v/v) (or other solvent systems) as an eluent. MP1 was obtained as a white powder with a 13% yield (35 mg).

### Antioxidant activity

Briefly, 400 µL solution of each synthesized compound with different concentrations (20–1000 µg/mL) was added to 1.6 mL DPPH solution (100 µmol/L). After shaking, the mixture was incubated at room temperature in the dark for 10 min, and then the absorbance was measured at 517 nm. The %Scavenging activity was calculated by using the formula:

$\% {\text{Scavenging Activity }}={\text{ }}[({{\text{A}}_{\text{c}}}-{{\text{A}}_{\text{t}}})/{{\text{A}}_{\text{c}}}] \cdot {\text{1}}00\% ,$ where, A_c_ = absorbance of the control sample, A_t_ = absorbance of the test sample after 10 min.

The scavenging activity was expressed as IC_50_ value, defined as the concentration (mM) of compound required for scavenging DPPH radicals by 50%. IC_50_ values were determined by linear regression analysis using at least three different concentrations in duplicate.

Three different concentrations were prepared for each sample, while each one was analyzed in duplicate. The obtained results were presented as mean values and standard deviation (SD). The statistical analysis was performed using Tukey’s HSD test (*p* < 0.05). The statistical software Statistica 10 software (StatSoft Inc., Tulsa, OK, USA) was implemented for this analysis.

### Biological evaluation

Research materials, Cell culture and Cell viability assay for AsPC-1 cell line, MPA derivatives preparation, PBMCs isolation, XTT dye-based in vitro cytotoxicity test, VPD450 dye-based in vitro cell proliferation test, enzyme inhibition in the presence of guanosine source (GMP test) (for T-Jurkat and PBMCs) and Statistical analyses were described in the Electronic Supplementary Information (ESI). AsPC-1 and T-Jurkat cell lines were purchased from ATCC, PBMCs (anonymous, healthy donors) were received as a gift from the Regional Centre for Blood Donation and Treatment in Gdańsk (Poland).

## Electronic supplementary material

Below is the link to the electronic supplementary material.

Supplementary Material 1

## Data Availability

All data generated or analysed during this study are included in this published article and its supplementary information files.
