# Supplementary material for: Direct synthesis of mycophenolic acid aryl esters with antioxidant and antiproliferative properties
Source: Sci Rep. 2025 Jul 17;15:26006. doi: 10.1038/s41598-025-11871-5 (PMC12271471; doi:10.1038/s41598-025-11871-5)
Supplement: Supplementary file 1 — Supplementary Material 1 [file 41598_2025_11871_MOESM1_ESM.pdf]

Electronic Supplementary Information (ESI)

Direct synthesis of mycophenolic acid aryl esters with antioxidant and antiproliferative properties

Juliusz Walczak[1], Dorota Iwaszkiewicz-Grześ[2], Magdalena Śliwka-Kaszyńska[1], Agnieszka Kurdyn[3], Ewa Augustin[3], Agnieszka Viapiana[4], Alina Plenis[4], Grzegorz Cholewiński[1,*]*

[1]Department of Organic Chemistry, Faculty of Chemistry, Gdańsk University of Technology, ul. G. Narutowicza 11/12, 80-233 Gdańsk, Poland.

[2]Department of Medical Immunology, Faculty of Medicine, Medical University of Gdansk, ul. Dębinki 7, 80-210 Gdańsk, Poland.

[3]Department of Pharmaceutical Technology and Biochemistry, Faculty of Chemistry, Gdańsk University of Technology, ul. G. Narutowicza 11/12, 80-233 Gdańsk, Poland.

[4]Department of Analytical Chemistry, Faculty of Pharmacy, Medical University of Gdańsk, Al. Gen. J. Hallera 107, 80-416 Gdańsk, Poland.

Supplementary materials .... **Page**

Growth inhibition of AsPC-1 cells after the treatment with the most promising antioxidants 2

Concentration-dependent change in viability under the treatment of ester derivatives of **MPA** 2

Concentration-dependent change in proliferation under the treatment of ester derivatives of **MPA** 3

Antiproliferative activity of various esters of MPA in the presence of guanosine source 4

Summarized properties of **MPA**, **MMF** and ester derivatives of **MPA** (**MP1**-**MP9**, **P1**-**P7**) 5-14

Biological evaluation — AsPC-1 cells 15

Biological evaluation — PBMC and T-Jurkat cells 16-17

^{1}H and ^{13}C NMR spectra of **MPA** 18-19

^{1}H and ^{13}C NMR spectra of **MMF** 20-21

^{1}H and ^{13}C NMR spectra of **MP1**-**MP9** 22-39

^{1}H and ^{13}C NMR spectra of **P1**-**P7** 40-53

HPLC chromatogram for **MPA** 54

HPLC chromatogram for **MMF** 55

HPLC chromatograms for **MP1**-**MP9** 56-64

HPLC chromatograms for **P1**-**P7** 65-71

MS spectrum of **MPA** and **MMF** 72

MS spectra of **MP1**-**MP9** 73-77

MS spectra of **P1**-**P7** 78-81

Remaining experiments (synthesis) 82-84

2D NMR spectra obtained for **P7** 85-91

* — Corresponding author; e-mail: grzegorz.cholewinski@pg.edu.pl

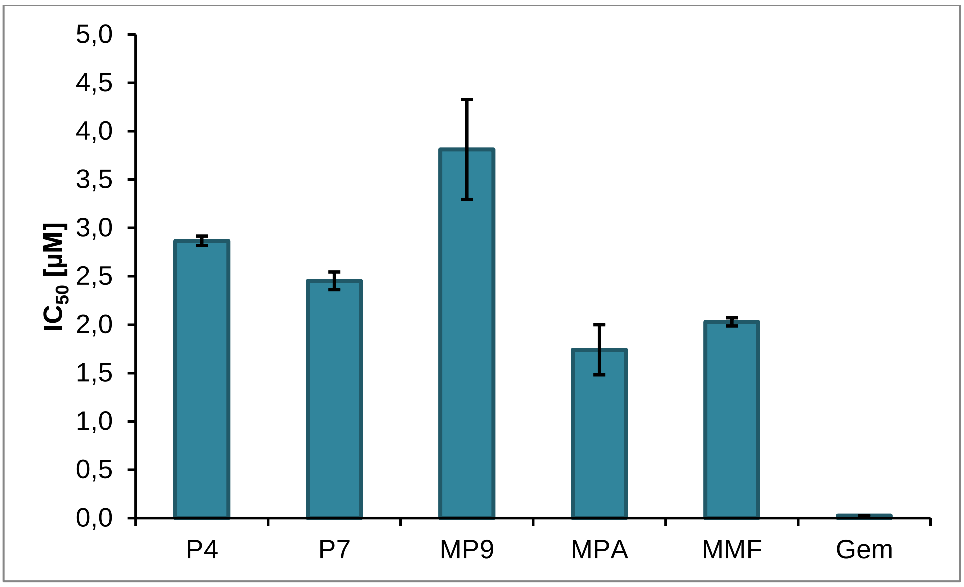

ESI Figure 1. Growth inhibition of AsPC-1 cells following treatment with the most promising antioxidants and gemcitabine (as a standard) for 72 hours (n = 3).

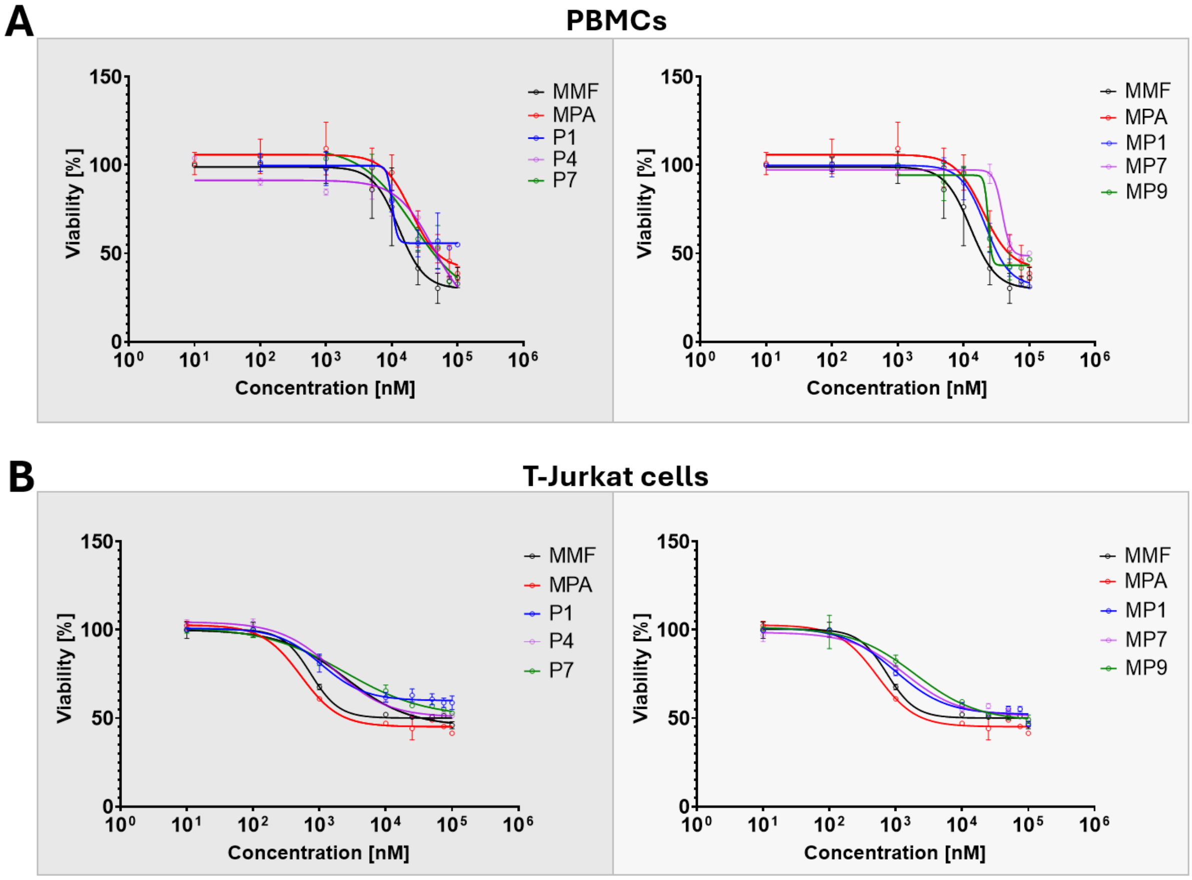

ESI Figure 2. Concentration-dependent change in viability under the treatment of ester derivatives of MPA. (A) PBMCs for 96 hours, and (B) T-Jurkat cell line for 48 hours, cultured in the presence of different concentrations (100; 50; 25; 10; 1; 0.1, and 0.01 µM) of MPA, MMF, and ester derivatives. The plots were separated based on the type of ester derivative (P or MP). The plates were subsequently incubated with XTT reagent for 24 hours. The orange formazan derived from a water-soluble tetrazolium compound serves as the basis for the colorimetric method performed on the microplate spectrophotometer. The XTT assay was conducted in at least three experiments and values are presented as mean ± SD.

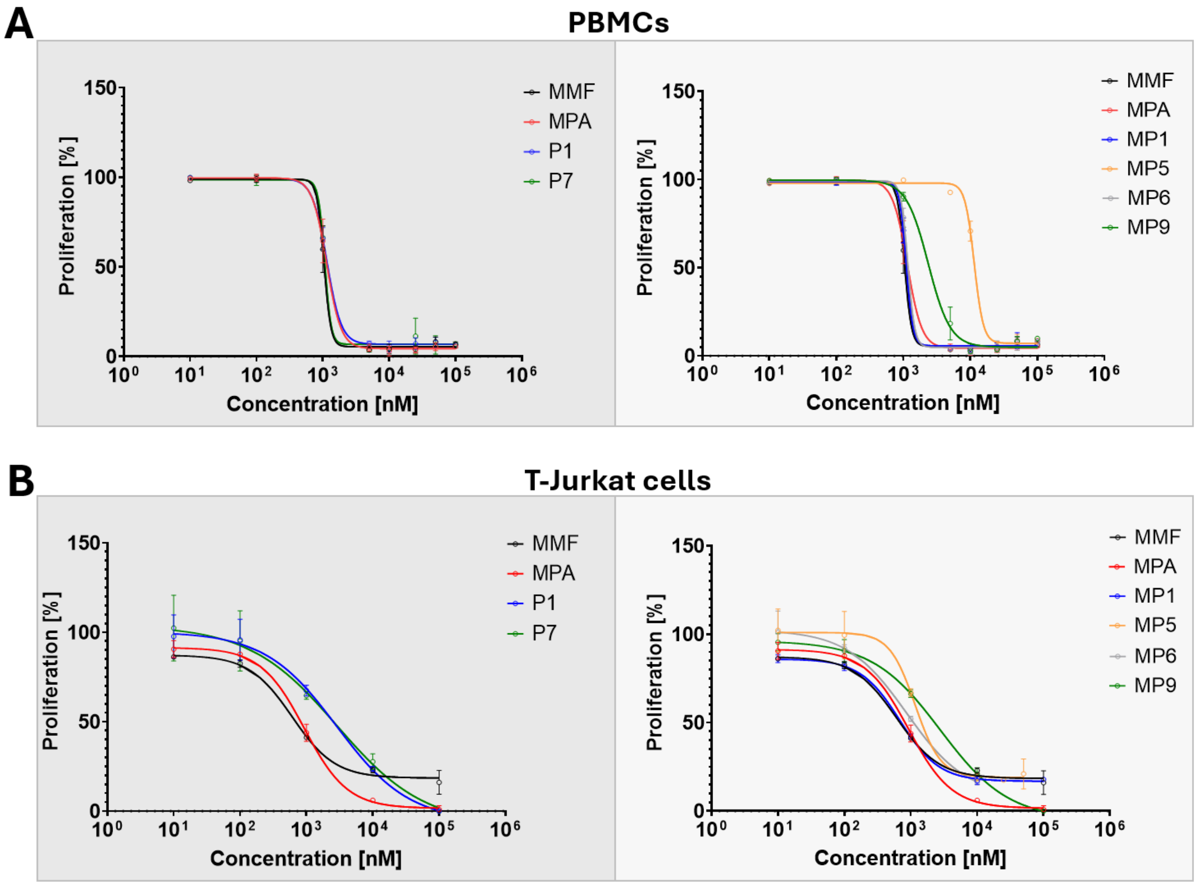

ESI Figure 3. Concentration-dependent change in proliferation under the treatment of ester derivatives of MPA. (A) VPD450-labelled PBMCs were cultured in the presence of different concentrations (100; 50; 25; 10; 1; 0.1, and 0.01 µM) of MPA, MMF, and ester derivatives, and stimulated with magnetic beads coated with anti-CD3 and anti-CD28 antibodies for 72 hours. (B) VPD450-labelled T-Jurkat cells were cultured in the presence of different concentrations (100; 10; 1; 0.1 and 0.01 µM) of MPA and ester derivatives for 48 hours. The plots were separated based on the type of ester derivative (P or MP). Cell proliferation was analyzed using flow cytometry. The results are expressed as the mean ± SD.

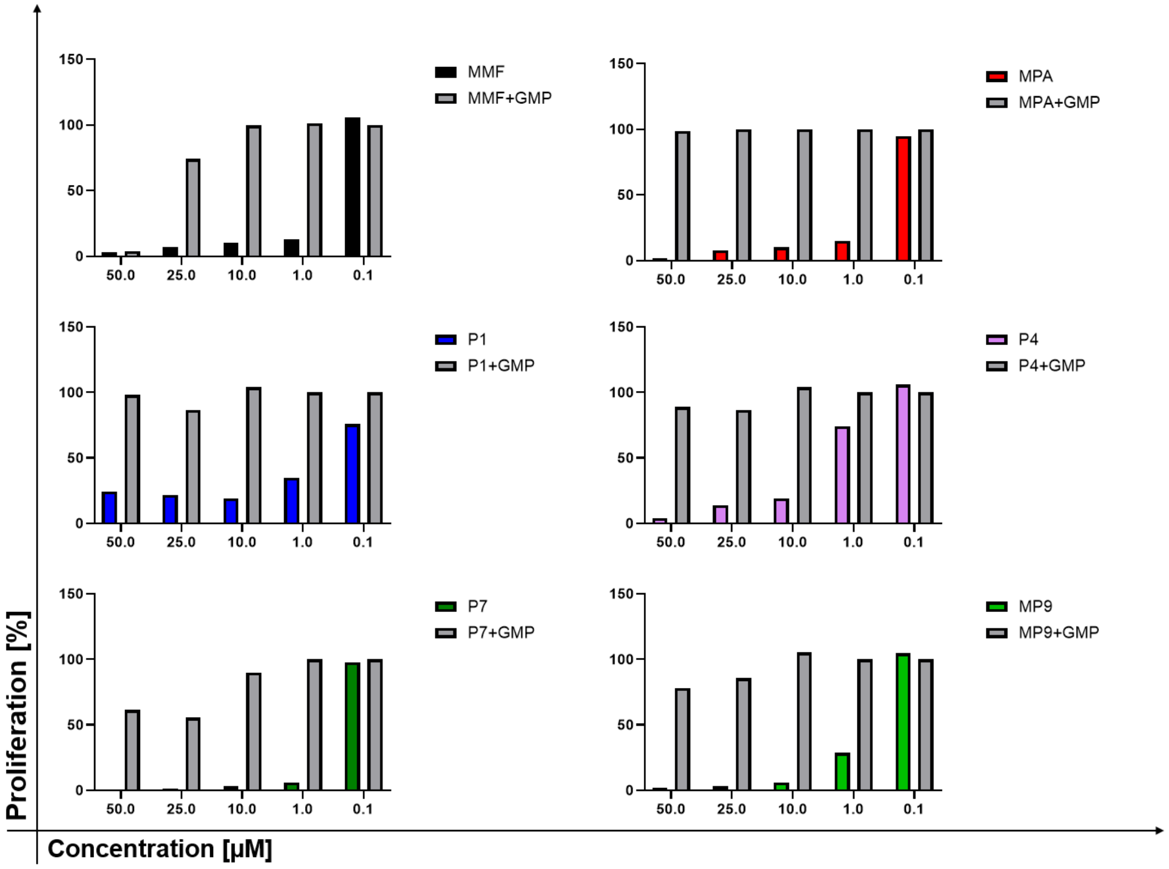

ESI Figure 4. Antiproliferative activity of MPA, MMF, and various esters of MPA in the presence of guanosine source — role of IMPDH inhibiton. VPD450-labeled human PBMCs, in the presence of different concentrations (50; 25; 10; 1, and 0.1 µM) of MPA, MMF, and esters, and stimulated (magnetic beads coated with anti-CD3 and anti-CD28 antibodies) were cultured with or without the addition of 50 µM GMP for 72 h. Cell proliferation was analyzed using flow cytometry.

Summarized properties of MPA, MMF and ester derivatives of MPA (MP1-MP9, P1-P7)

Mycophenolic acid (**MPA**):

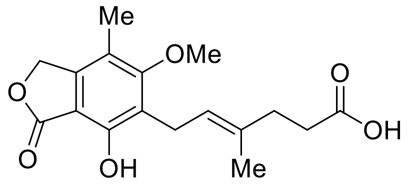

R_F = 0.42 (toluene:acetone, 3:1, v/v). ^{1}H NMR (DMSO-*d$_6$*) δ: 12.01 (s, 1H, -COOH), 9.40 (s, 1H, Ar-OH), 5.24 (s, 2H, Ar-CH$_2$O-), 5.13 (t, *J* = 6.9 Hz, 1H, >C=CH-), 3.69 (s, 3H, Ar-OCH$_3$), 3.29 (d, *J* = 6.8 Hz, 2H, Ar-CH$_2$-), 2.26 (t, *J* = 7.4 Hz, 2H, -CH$_2$-), 2.16 (t, *J* = 7.5 Hz, 2H, -CH$_2$), 2.08 (s, 3H, Ar-CH$_3$), 1.74 (s, 3H, -CH$_3$); ^{13}C NMR (DMSO-*d$_6$*) δ 174.52 (-COOH), 170.58 (-COO-), 163.01 (C$_{Ar}$-OMe), 153.16 (C$_{Ar}$-OH), 146.27 (C$_{Ar}$-CH$_2$O-), 134.04 (>C=C(-CH$_3$)-), 123.13 (C$_{Ar}$-CH$_2$-CH=), 122.85 (>C=C(-CH$_3$)-), 116.40 (C$_{Ar}$-CH$_3$), 107.41 (C$_{Ar}$-COO-), 69.06 (Ar-CH$_2$O-), 61.08 (Ar-OCH$_3$), 34.57 (-CH$_2$-COOH), 32.91 (-CH$_2$-CH$_2$-), 22.87 (Ar-CH$_2$-), 16.46 (=C(-CH$_3$)-), 11.51 (Ar-CH$_3$). $R_{T,HPLC}$ = 7.382 min, purity: 100.00%; HRMS (*m/z*) calculated for $C_{17}H_{20}O_6$ [M — H]$^-$: 319.1182; found: 319.1242.

Mycophenolate mofetil (**MMF**):

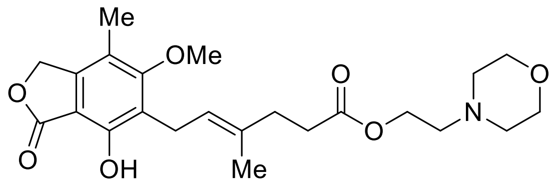

R_F = 0.58 (dichloromethane:methanol, 20:1, v/v). ^{1}H NMR (CDCl$_3$) δ 7.97 (s, 1H, Ar-OH), 5.23 – 5.16 (m, 3H, >C=CH- and Ar-CH$_2$O-), 4.12 (t, *J* = 5.8 Hz, 2H, -COOCH$_2$-), 3.74 (s, 3H, Ar-OCH$_3$), 3.72 – 3.64 (m, 4H, -CH$_2$-O-CH$_2$-), 3.36 (d, *J* = 6.9 Hz, 2H, Ar-CH$_2$-), 2.57 (t, *J* = 5.8 Hz, 2H, -CH$_2$-N<), 2.48 (s, 4H, -CH$_2$-N-CH$_2$-), 2.39 (t, *J* = 7.7 Hz, 2H, -CH$_2$-), 2.28 (t, *J* = 7.6 Hz, 2H, -CH$_2$-), 2.13 (s, 3H, Ar-CH$_3$), 1.78 (s, 3H, -CH$_3$). ^{13}C NMR (CDCl$_3$) δ 173.19 (-COO-CH$_2$-CH$_2$-N<), 172.76 (-COO-), 163.54 (C$_{Ar}$-OMe), 153.60 (C$_{Ar}$-OH), 144.06 (C$_{Ar}$-CH$_2$O-), 133.92 (>C=C(-CH$_3$)-), 122.81 (C$_{Ar}$-CH$_2$-CH=), 122.16 (>C=C(-CH$_3$)-), 116.60 (C$_{Ar}$-CH$_3$), 106.35 ((C$_{Ar}$-COO-)), 69.93 (Ar-CH$_2$O-), 66.83 (2C, -CH$_2$-O-CH$_2$-), 61.71 (-COO-CH$_2$-CH$_2$-N<), 60.99 (Ar-OCH$_3$), 57.01 (COOCH$_2$-CH$_2$-N<), 53.91 (2C, -CH$_2$-N-CH$_2$-), 34.63 (-CH$_2$-COO-CH$_2$-CH$_2$-), 32.97 (-CH$_2$-CH$_2$-), 22.62 (Ar-CH$_2$-), 16.07 (=C(-CH$_3$)-), 11.54 (Ar-CH$_3$). $R_{T,HPLC}$ = 14.093 min, purity: 100.00%; HRMS (*m/z*) calculated for $C_{23}H_{31}NO_7$ [M — H]$^-$: 432.2022; found: 432.2100.

Guaiacol mycophenolate (**MP1**):

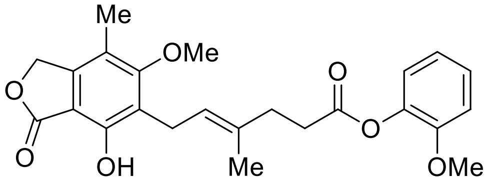

Eluent: petroleum ether:ethyl acetate, 3:1 (v/v). Obtained as a white solid with a 13% yield. R_F = 0.33 (dichloromethane). ^{1}H NMR ($CDCl_3$) δ 7.68 (s, 1H, Ar-OH), 7.17 (t, *J* = 7.7 Hz, 1H, $C_{Ar,MP}$(6)-H), 6.91 (dt, *J* = 15.2, 7.9 Hz, 3H, $C_{Ar,MP}$(3, 4 and 5)-H)., 5.33 (t, *J* = 6.9 Hz, 1H, >C=CH-), 5.20 (s, 2H, Ar-CH_2O-), 3.82 – 3.73 (m, 6H, Ar-OCH_3 and Ar_{MP}-OCH_3), 3.41 (t, *J* = 10.4 Hz, 2H, Ar-CH_2-), 2.72 – 2.63 (m, 2H, -CH_2-), 2.44 (dd, *J* = 18.9, 11.4 Hz, 2H, -CH_2-), 2.15 (s, 3H, Ar-CH_3), 1.86 (s, 3H, -CH_3). ^{13}C NMR ($CDCl_3$) δ 172.92 (-$\underline{C}$OO-Ar_{MP}), 171.38 (-$\underline{C}$OO-), 163.67 ($\underline{C}_{Ar}$-OMe), 153.59 ($\underline{C}_{Ar}$-OH), 151.07 ($\underline{C}_{Ar,MP}$-OMe), 144.01 ($\underline{C}_{Ar}$-CH_2O-), 139.70 ($\underline{C}_{Ar,MP}$-OOC-), 134.02 (>C=$\underline{C}$(-CH_3)-), 126.74 ($C_{Ar,MP}$(4)-H), 122.92 ($\underline{C}_{Ar}$-CH_2-CH=), 122.76 (>$\underline{C}$=C(-CH_3)-), 122.10 ($C_{Ar,MP}$(5)-H), 120.62 ($C_{Ar,MP}$(6)-H), 116.74 ($\underline{C}_{Ar}$-CH_3), 112.31 ($C_{Ar,MP}$(3)-H), 106.34 ($\underline{C}_{Ar}$-COO-), 70.05 (Ar-$\underline{C}H_2$O-), 61.01 (Ar-O$\underline{C}H_3$), 55.77 (Ar_{MP}-O$\underline{C}H_3$), 34.60 (-$\underline{C}H_2$-COO-Ar_{MP}), 32.75 (-$\underline{C}H_2$-CH_2-), 22.62 (Ar-$\underline{C}H_2$-), 16.17 (=C(-$\underline{C}H_3$)-), 11.58 (Ar-$\underline{C}H_3$). $R_{T,HPLC}$ = 14.093 min, purity: 100.00%; HRMS (*m/z*) calculated for $C_{24}H_{26}O_7$ [M — H]$^-$: 425.1600; found: 425.1673.

Syringol mycophenolate (**MP2**):

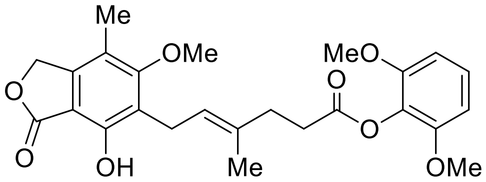

Eluent: chloroform. Obtained as a low-melting white powder with a 36% yield. R_F = 0.22 (dichloromethane). ^{1}H NMR ($CDCl_3$) δ 7.68 (s, 1H, Ar-OH), 7.11 (t, *J* = 8.4 Hz, 1H, $C_{Ar,MP}$(4)-H), 6.59 (d, *J* = 8.4 Hz, 2H, $C_{Ar,MP}$(3 and 5)-H), 5.32 (t, *J* = 6.8 Hz, 1H, >C=CH-), 5.20 (s, 2H, Ar-CH_2O-), 3.77 (d, *J* = 10.0 Hz, 9H, Ar-OCH_3 and Ar_{MP}-OCH_3), 3.42 (d, *J* = 6.9 Hz, 2H, Ar-CH_2-), 2.76 – 2.65 (m, 2H, -CH_2-), 2.52 – 2.41 (m, 2H, -CH_2-), 2.15 (s, 3H, Ar-CH_3), 1.86 (s, 3H, -CH_3). ^{13}C NMR ($CDCl_3$) δ 172.93 (-$\underline{C}$OO-Ar_{MP}), 171.14 (-$\underline{C}$OO-), 163.70 ($\underline{C}_{Ar}$-OMe), 153.59 ($\underline{C}_{Ar}$-OH), 152.28 (2C, $\underline{C}_{Ar,MP}$-OMe), 143.99 ($\underline{C}_{Ar}$-CH_2O-), 134.21 (>C=$\underline{C}$(-CH_3)-), 128.68 ($\underline{C}_{Ar,MP}$-OOC-), 126.11 ($C_{Ar,MP}$(4)-H), 122.76 ($\underline{C}_{Ar}$-CH_2-CH=), 122.15 (>$\underline{C}$=C(-CH_3)-), 116.72 ($\underline{C}_{Ar}$-CH_3), 106.32 ($\underline{C}_{Ar}$-COO-), 104.78 (2C, $C_{Ar,MP}$(3 and 5)-H), 70.05 (Ar-$\underline{C}H_2$O-), 61.02 (Ar-O$\underline{C}H_3$), 56.07 (2C, Ar_{MP}-O$\underline{C}H_3$), 34.57 (-$\underline{C}H_2$-COO-Ar_{MP}), 32.64 (-$\underline{C}H_2$-CH_2-), 22.62 (Ar-$\underline{C}H_2$-), 16.15 (=C(-$\underline{C}H_3$)-), 11.57 (Ar-$\underline{C}H_3$). $R_{T,HPLC}$ = 13.838 min, purity: 97.40%; HRMS (*m/z*) calculated for $C_{25}H_{28}O_8$ [M — H]$^-$: 455.1706; found: 455.1788.

Syringaldehyde mycophenolate (**MP3**):

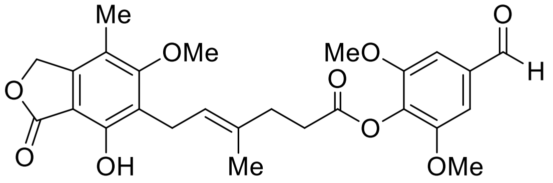

Eluent: chloroform. Obtained as a low-melting white precipitate with a 18% yield. R_F = 0.11 (dichloromethane). ^{1}H NMR ($CDCl_3$) δ 9.89 (s, 1H, Ar_{MP}-CHO), 7.68 (s, 1H, Ar-OH), 7.14 (d, *J* = 11.1 Hz, 2H, $C_{Ar,MP}$(3 and 5)-H), 5.33 (t, *J* = 7.5, 6.4 Hz, 1H, >C=CH-), 5.19 (s, 2H, Ar-CH_2O-), 3.87 (s, 6H, Ar_{MP}-OCH_3), 3.76 (s, 3H, Ar-OCH_3), 3.42 (d, *J* = 6.9 Hz, 2H, Ar-CH_2-), 2.72 (dd, *J* = 9.0, 6.9 Hz, 2H, -CH_2-), 2.49 – 2.40 (m, 2H, -CH_2-), 2.15 (s, 3H, Ar-CH_3), 1.86 (s, 3H, -CH_3). ^{13}C NMR ($CDCl_3$) δ 191.06 (Ar_{MP}-$\underline{C}$HO), 172.92 (-$\underline{C}$OO-Ar_{MP}), 170.46 (-$\underline{C}$OO-), 163.66 ($\underline{C}_{Ar}$-OMe), 153.59 ($\underline{C}_{Ar}$-OH), 152.86 (2C, $\underline{C_{Ar,MP}}$-OMe), 144.02 ($\underline{C}_{Ar}$-CH_2O-), 134.19 (>C=$\underline{C}$(-CH_3)-), 133.95 ($\underline{C_{Ar,MP}}$-OOC-), 133.82 ($\underline{C_{Ar,MP}}$-CHO), 122.91 ($\underline{C}_{Ar}$-CH_2-CH=), 122.07 (>$\underline{C}$=C(-CH_3)-), 116.73 ($\underline{C}_{Ar}$-CH_3), 106.35 ($\underline{C}_{Ar}$-COO-), 106.03 (2C, $C_{Ar,MP}$(3 and 5)-H), 70.05 (Ar-$\underline{C}H_2$O-), 61.01 (Ar-O$\underline{C}H_3$), 56.31 (2C, Ar_{MP}-O$\underline{C}H_3$), 34.46 (-$\underline{C}H_2$-COO-Ar_{MP}), 32.58 (-$\underline{C}H_2$-CH_2-), 22.63 (Ar-$\underline{C}H_2$-), 16.15 (=C(-$\underline{C}H_3$)-), 11.58 (Ar-$\underline{C}H_3$). $R_{T,HPLC}$ = 13.452 min, purity: 100.00%; HRMS (*m/z*) calculated for $C_{26}H_{28}O_9$ [M — H]$^-$: 483.1655; found: 483.1742.

Vanillin mycophenolate (**MP4**):

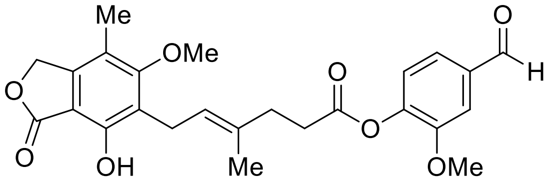

Eluent: petroleum ether:ethyl acetate, 3:1 (v/v). Obtained as a transparent oil with a 14% yield. R_F = 0.28 (dichloromethane). ^{1}H NMR ($CDCl_3$) δ 9.93 (s, 1H, Ar_{MP}-CHO), 7.69 (s, 1H, Ar-OH), 7.47 (d, *J* = 1.5 Hz, 1H, $C_{Ar,MP}$(3)-H), 7.42 (dd, *J* = 8.0, 1.6 Hz, 1H, $C_{Ar,MP}$(5 or 6)-H), 7.13 (d, *J* = 8.0 Hz, 1H, $C_{Ar,MP}$(5 or 6)-H), 5.34 (t, *J* = 6.7 Hz, 1H, >C=CH-), 5.20 (s, 2H, Ar-CH_2O-), 3.87 (s, 3H, Ar_{MP}-OCH_3), 3.77 (s, 3H, Ar-OCH_3), 3.42 (d, *J* = 6.9 Hz, 2H, Ar-CH_2-), 2.74 – 2.65 (m, 2H, -CH_2-), 2.45 (t, *J* = 7.7 Hz, 2H, -CH_2-), 2.16 (d, *J* = 10.8 Hz, 3H, Ar-CH_3), 1.86 (s, 3H, -CH_3). ^{13}C NMR ($CDCl_3$) δ 191.06 (Ar_{MP}-$\underline{C}$HO), 172.91 (-$\underline{C}$OO-Ar_{MP}), 170.72 (-$\underline{C}$OO-), 163.63 ($\underline{C}_{Ar}$-OMe), 153.59 ($\underline{C}_{Ar}$-OH), 151.92 ($\underline{C_{Ar,MP}}$-OMe), 144.94 ($\underline{C_{Ar,MP}}$-OOC-), 144.05 ($\underline{C}_{Ar}$-CH_2O-), 135.06 ($\underline{C_{Ar,MP}}$-CHO), 133.78 (>C=$\underline{C}$(-CH_3)-), 124.62 ($C_{Ar,MP}$(6)-H), 123.37 ($C_{Ar,MP}$(5)-H), 123.08 ($\underline{C}_{Ar}$-CH_2-CH=), 122.01 (>$\underline{C}$=C(-CH_3)-), 116.76 ($\underline{C}_{Ar}$-CH_3), 110.75 ($C_{Ar,MP}$(3)-H), 106.37 ($\underline{C}_{Ar}$-COO-), 70.06 (Ar-$\underline{C}H_2$O-), 61.01 (Ar-O$\underline{C}H_3$), 56.02 (Ar_{MP}-O$\underline{C}H_3$), 34.49 ($\underline{C}H_2$-COO-Ar_{MP}), 32.70 (-$\underline{C}H_2$-CH_2-), 22.62 (Ar-$\underline{C}H_2$-), 16.15 (=C(-$\underline{C}H_3$)-), 11.58 (Ar-$\underline{C}H_3$). $R_{T,HPLC}$ = 13.334 min, purity: 99.70%; HRMS (*m/z*) calculated for $C_{25}H_{26}O_8$ [M — H]$^-$: 453.1549; found: 453.1632.

Dehydrozingerone mycophenolate (**MP5**):

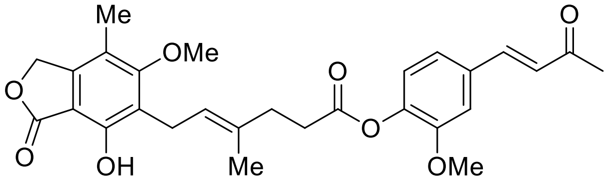

Eluent: petroleum ether:ethyl acetate, 3:1 (v/v). Obtained as a yellowish powder with a 12% yield. R_F = 0.10 (dichloromethane). ^{1}H NMR ($CDCl_3$) δ 7.69 (s, 1H, Ar-OH), 7.45 (d, *J* = 16.2 Hz, 1H, Ar$_{MP}$-CH=), 7.09 (d, *J* = 7.3 Hz, 2H, C$_{Ar,MP}$-H), 6.98 (d, *J* = 8.4 Hz, 1H, C$_{Ar,MP}$-H), 6.65 (d, *J* = 16.2 Hz, 1H, >C=CH-(MP)), 5.33 (t, *J* = 6.9 Hz, 1H, >C=CH-), 5.20 (s, 2H, Ar-CH$_2$O-), 3.83 (s, 3H, Ar$_{MP}$-OCH$_3$), 3.76 (s, 3H, Ar-OCH$_3$), 3.42 (d, *J* = 6.9 Hz, 2H, Ar-CH$_2$-), 2.73 – 2.63 (m, 2H, -CH$_2$-), 2.44 (t, *J* = 7.7 Hz, 2H, -CH$_2$-), 2.38 (s, 3H, -(C=O)-CH$_3$ (MP)), 2.15 (s, 3H, Ar-CH$_3$), 1.86 (s, 3H, -CH$_3$). ^{13}C NMR ($CDCl_3$) δ 198.26 (-CH=CH-($\underline{C}$=O)-CH$_3$ (MP)), 172.91 (-$\underline{C}$OO-Ar$_{MP}$), 171.08 (-$\underline{C}$OO), 163.65 ($\underline{C}_{Ar}$-OMe), 153.58 ($\underline{C}_{Ar}$-OH), 151.43 ($\underline{C}_{Ar,MP}$-OMe), 144.03 ($\underline{C}_{Ar}$-CH$_2$O-), 142.72 (C$_{Ar,MP}$-$\underline{C}$H=CH-), 141.62 ($\underline{C}_{Ar,MP}$-OOC-), 133.86 (>C=$\underline{C}$(-CH$_3$)-), 133.20 ($\underline{C}_{Ar,MP}$-CH=CH-), 127.21 (C$_{Ar,MP}$-CH=$\underline{C}$H-), 123.29 (C$_{Ar,MP}$(6)-H), 123.01 ($\underline{C}_{Ar}$-CH$_2$-CH=), 122.04 (>$\underline{C}$=C(-CH$_3$)-), 121.42 (C$_{Ar,MP}$(5)-H), 116.75 ($\underline{C}_{Ar}$-CH$_3$), 111.15 (C$_{Ar,MP}$(3)-H), 106.35 ($\underline{C}_{Ar}$-COO-), 70.06 (Ar-$\underline{C}$H$_2$O-), 61.01 (Ar-O$\underline{C}$H$_3$), 55.86 (Ar$_{MP}$-O$\underline{C}$H$_3$), 34.52 (-$\underline{C}$H$_2$-COO-Ar$_{MP}$), 32.71 (-$\underline{C}$H$_2$-CH$_2$-), 27.53 (-CH$_2$-(C=O)-$\underline{C}$H$_3$ (MP)), 22.62 (Ar-$\underline{C}$H$_2$-), 16.16 (=C(-$\underline{C}$H$_3$)-),), 11.58 (Ar-$\underline{C}$H$_3$). $R_{T,HPLC}$ = 14.068 min, purity: 98.50%; HRMS (*m/z*) calculated for $C_{28}H_{30}O_8$ [M — H]$^-$: 493.1862; found: 493.1951.

Zingerone mycophenolate (**MP6**):

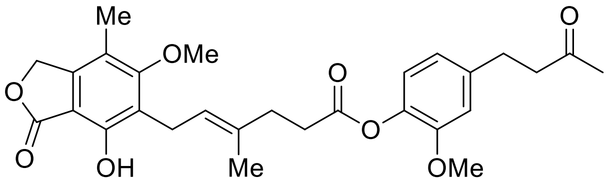

Eluent: petroleum ether:ethyl acetate, 3:1 (v/v). Obtained as a transparent oil with a 19% yield. R_F = 0.10 (dichloromethane). ^{1}H NMR ($CDCl_3$) δ 7.68 (s, 1H, Ar-OH), 6.82 (d, *J* = 8.0 Hz, 1H, C$_{Ar,MP}$-H), 6.76 (d, *J* = 1.6 Hz, 1H, C$_{Ar,MP}$-H), 6.68 (dd, *J* = 8.1, 1.7 Hz, 1H, C$_{Ar,MP}$-H), 5.32 (t, *J* = 6.5 Hz, 1H, >C=CH-), 5.20 (s, 2H, Ar-CH$_2$O-), 3.77 (d, *J* = 6.4 Hz, 6H, Ar-OCH$_3$ and Ar$_{MP}$-OCH$_3$), 3.42 (d, *J* = 6.9 Hz, 2H, Ar-CH$_2$-), 2.86 (t, *J* = 7.5 Hz, 2H, Ar$_{MP}$-CH$_2$-), 2.75 (t, *J* = 7.5 Hz, 2H, -CH$_2$-C(=O)- (MP)), 2.69 – 2.61 (m, 2H, -CH$_2$-), 2.43 (t, *J* = 7.7 Hz, 2H, -CH$_2$-), 2.16 (d, *J* = 11.9 Hz, 6H, Ar-CH$_3$ and -(C=O)-CH$_3$ (MP)), 1.85 (s, 3H, -CH$_3$). ^{13}C NMR ($CDCl_3$) δ 207.79 (-CH$_2$-($\underline{C}$=O)-CH$_3$ (MP)), 172.92 (-$\underline{C}$OO-Ar$_{MP}$), 171.53 (-$\underline{C}$OO-), 163.66 ($\underline{C}_{Ar}$-OMe), 153.59 ($\underline{C}_{Ar}$-OH), 150.83 ($\underline{C}_{Ar,MP}$-OMe), 144.01 ($\underline{C}_{Ar}$-CH$_2$O-), 139.89 ($\underline{C}_{Ar,MP}$-OOC-), 137.91 ($\underline{C}_{Ar,MP}$-CH$_2$-CH$_2$-), 134.02 (>C=$\underline{C}$(-CH$_3$)-), 122.89 ($\underline{C}_{Ar}$-CH$_2$-CH=), 122.57 (C$_{Ar,MP}$(6)-H), 122.10 (>$\underline{C}$=C(-CH$_3$)-), 120.20 (C$_{Ar,MP}$(5)-H), 116.74 ($\underline{C}_{Ar}$-CH$_3$), 112.58 (C$_{Ar,MP}$(3)-H), 106.34 ($\underline{C}_{Ar}$-COO-), 70.06 (Ar-$\underline{C}$H$_2$O-), 61.01 (Ar-O$\underline{C}$H$_3$), 55.77 (Ar$_{MP}$-O$\underline{C}$H$_3$), 45.12 (-$\underline{C}$H$_2$-(C=O)-CH$_3$ (MP)), 34.59 (-$\underline{C}$H$_2$-COO-Ar$_{MP}$), 32.74 (-$\underline{C}$H$_2$-CH$_2$-), 30.13 (-CH$_2$-(C=O)-$\underline{C}$H$_3$ (MP)), 29.54 (Ar$_{MP}$-$\underline{C}$H$_2$-CH$_2$-(C=O)-), 22.61 (Ar-$\underline{C}$H$_2$-), 16.16 (=C(-$\underline{C}$H$_3$)-), 11.58 (Ar-$\underline{C}$H$_3$). $R_{T,HPLC}$ = 14.332 min, purity: 97.70%; HRMS (*m/z*) calculated for $C_{28}H_{32}O_8$ [M — H]$^-$: 495.2019; found: 495.2118.

(Methyl ferulate) mycophenolate (**MP7**):

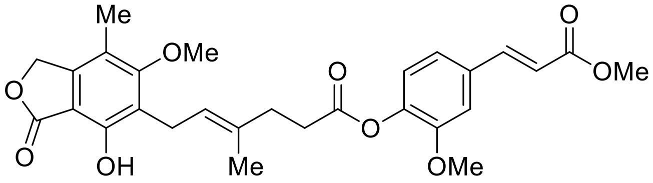

Eluent: dichloromethane. Obtained as a yellowish powder with a 14% yield. R_F = 0.15 (dichloromethane). ^{1}H NMR ($CDCl_3$) δ 7.69 (s, 1H, Ar-OH), 7.63 (d, *J* = 16.0 Hz, 1H, Ar_{MP}-CH=), 7.06 (d, *J* = 5.7 Hz, 2H, $C_{Ar,MP}$-H), 6.95 (d, *J* = 8.6 Hz, 1H, $C_{Ar,MP}$-H), 6.37 (d, *J* = 16.0 Hz, 1H, >C=CH- (MP)), 5.33 (t, *J* = 6.8 Hz, 1H, >C=CH-), 5.20 (s, 2H, Ar-CH_2O-), 3.81 (d, *J* = 9.4 Hz, 6H, Ar-OCH_3 and Ar_{MP}-OCH_3), 3.76 (s, 3H, -$COOCH_3$ (MP)), 3.42 (d, *J* = 6.9 Hz, 2H, Ar-CH_2-), 2.68 (t, *J* = 7.7 Hz, 2H, -CH_2-), 2.44 (t, *J* = 7.7 Hz, 2H, -CH_2-), 2.14 (s, 3H, Ar-CH_3), 1.86 (s, 3H, -CH_3). ^{13}C NMR ($CDCl_3$) δ 172.91 (-$\underline{C}$OO-Ar_{MP}), 171.11 (-$\underline{C}$OO-), 167.26 (-$\underline{C}OOCH_3$ (MP)), 163.64 ($\underline{C}_{Ar}$-OMe), 153.59 ($\underline{C}_{Ar}$-OH), 151.35 ($\underline{C}_{\underline{Ar,MP}}$-OMe), 144.15 ($Ar_{MP}$-$\underline{C}$H=CH-), 144.02 ($\underline{C}_{Ar}$-$CH_2$O-), 141.42 ($\underline{C}_{\underline{Ar,MP}}$-OOC-), 133.88 (>C=$\underline{C}$(-$CH_3$)-), 133.16 ($\underline{C}_{\underline{Ar,MP}}$-CH=CH-), 123.21 ($C_{Ar,MP}$(6)-H), 122.99 ($\underline{C}_{Ar}$-$CH_2$-CH=), 122.05 (>$\underline{C}$=C(-$CH_3$)-), 121.10 ($C_{Ar,MP}$(5)-H), 117.90 ($Ar_{MP}$-CH=$\underline{C}$H-), 116.74 ($\underline{C}_{Ar}$-$CH_3$), 111.15 ($C_{Ar,MP}$(3)-H), 106.35 ($\underline{C}_{Ar}$-COO-), 70.05 (Ar-$\underline{C}H_2$O-), 61.01 (Ar-O$\underline{C}H_3$), 55.84 ($Ar_{MP}$-O$\underline{C}H_3$), 51.76 (-COO$\underline{C}H_3$ (MP)), 34.53 (-$\underline{C}H_2$-COO-Ar_{MP}), 32.72 (-$\underline{C}H_2$-CH_2-), 22.62 (Ar-$\underline{C}H_2$-), 16.16 (=C(-$\underline{C}H_3$)-), 11.58 (Ar-$\underline{C}H_3$). $R_{T,HPLC}$ = 15.660 min, purity: 100.00%; HRMS (*m/z*) calculated for $C_{28}H_{30}O_9$ [M — H]$^-$: 509.1811; found: 509.1908.

Eugenol mycophenolate (**MP8**):

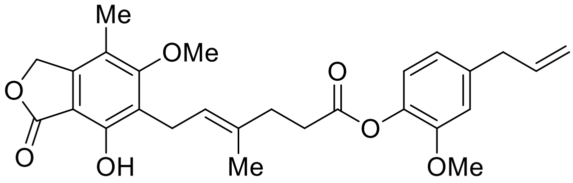

Eluent: dichloromethane. Obtained as a dense white solid with a 30% yield. R_F = 0.30 (dichloromethane). ^{1}H NMR ($CDCl_3$) δ 7.71 (s, 1H, Ar-OH), 6.85 (d, *J* = 8.0 Hz, 1H, $C_{Ar,MP}$-H), 6.78 (s, 1H, $C_{Ar,MP}$-H), 6.71 (t, *J* = 8.6 Hz, 1H, $C_{Ar,MP}$-H), 5.97 (ddt, *J* = 16.8, 10.1, 6.7 Hz, 1H, -CH=C< (MP)), 5.35 (t, *J* = 6.8 Hz, 1H, >C=CH-), 5.22 (s, 2H, Ar-CH_2O-), 5.11 (dd, *J* = 13.3, 6.3 Hz, 2H, >C=CH_2 (MP)), 3.80 (t, *J* = 6.6 Hz, 6H, Ar-OCH_3 and Ar_{MP}-OCH_3), 3.44 (d, *J* = 6.9 Hz, 2H, Ar-CH_2-), 3.38 (d, *J* = 6.7 Hz, 2H, Ar_{MP}-CH_2-), 2.73 – 2.64 (m, 2H, -CH_2-), 2.46 (t, *J* = 7.7 Hz, 2H, -CH_2-), 2.17 (s, 3H, Ar-CH_3), 1.88 (s, 3H, -CH_3). ^{13}C NMR ($CDCl_3$) δ 172.92 (-$\underline{C}$OO-Ar_{MP}), 171.52 (-$\underline{C}$OO-), 163.67 ($\underline{C}_{Ar}$-OMe), 153.60 ($\underline{C}_{Ar}$-OH), 150.82 ($\underline{C}_{\underline{Ar,MP}}$-OMe), 144.00 ($\underline{C}_{Ar}$-$CH_2$O-), 138.83 ($\underline{C}_{\underline{Ar,MP}}$-OOC-), 137.90 ($\underline{C}_{\underline{Ar,MP}}$-$CH_2$-CH=$CH_2$), 137.04 ($Ar_{MP}$-$CH_2$-$\underline{C}$H=$CH_2$), 134.04 (>C=$\underline{C}$(-$CH_3$)-), 122.90 ($\underline{C}_{Ar}$-$CH_2$-CH=), 122.46 ($C_{Ar,MP}$(6)-H), 122.10 (>$\underline{C}$=C(-$CH_3$)-), 120.52 ($C_{Ar,MP}$(5)-H), 116.74 ($\underline{C}_{Ar}$-$CH_3$), 116.12 ($Ar_{MP}$-$CH_2$-CH=$\underline{C}H_2$), 112.63 ($C_{Ar,MP}$(3)-H), 106.34 ($\underline{C}_{Ar}$-COO-), 70.05 (Ar-$\underline{C}H_2$O-), 61.01 (Ar-O$\underline{C}H_3$), 55.75 ($Ar_{MP}$-O$\underline{C}H_3$), 40.07 ($Ar_{MP}$-$\underline{C}H_2$-CH=$CH_2$), 34.61 (-$\underline{C}H_2$-COO-$Ar_{MP}$), 32.76 (-$\underline{C}H_2$-$CH_2$-), 22.62 (Ar-$\underline{C}H_2$-), 16.17 (=C(-$\underline{C}H_3$)-), 11.58 (Ar-$\underline{C}H_3$). $R_{T,HPLC}$ = 16.843 min, purity: 100.00%; HRMS (*m/z*) calculated for $C_{27}H_{30}O_7$ [M — H]$^-$: 465.1913; found: 465.1994.

Isoeugenol mycophenolate (**MP9**):

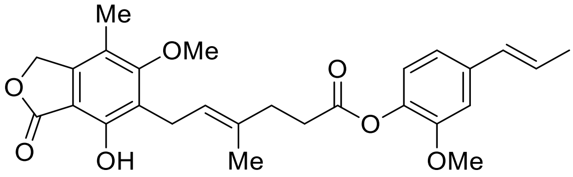

Eluent: dichloromethane. Obtained as a transparent oil with an 18% yield. R_F = 0.37 (dichloromethane). ^{1}H NMR ($CDCl_3$) δ 7.68 (s, 1H, Ar-OH), 6.88 (d, *J* = 6.0 Hz, 1H, $C_{Ar,MP}$(3)-H), 6.85 – 6.80 (m, 2H, $C_{Ar,MP}$(5 or 6)-H), 6.40 – 6.30 (m, 1H, Ar_{MP}-CH=C<), 6.17 (dq, *J* = 13.3, 6.6 Hz, 1H, >C=CH- (MP)), 5.33 (t, *J* = 6.8 Hz, 1H, >C=CH-), 5.20 (s, 2H, Ar-CH_2O-), 3.78 (d, *J* = 17.8 Hz, 6H, Ar-OCH_3 and Ar_{MP}-OCH_3), 3.42 (d, *J* = 6.9 Hz, 2H, Ar-CH_2-), 2.67 (dd, *J* = 14.9, 7.6 Hz, 2H, -CH_2-), 2.44 (t, *J* = 7.7 Hz, 2H, -CH_2-), 2.14 (s, 3H, Ar-CH_3), 1.91 – 1.83 (m, 6H, -CH_3 and -CH_3 (MP)). ^{13}C NMR ($CDCl_3$) δ 172.92 (-$\underline{C}$OO-Ar_{MP}), 171.46 (-$\underline{C}$OO-), 163.67 ($\underline{C}_{Ar}$-OMe), 153.59 ($\underline{C}_{Ar}$-OH), 150.93 ($\underline{C}_{Ar,MP}$-OMe), 144.00 ($\underline{C}_{Ar}$-CH_2O-), 138.51 ($\underline{C}_{Ar,MP}$-OOC-), 136.88 ($\underline{C}_{Ar,MP}$-CH=CH-CH_3), 134.01 (>C=$\underline{C}$(-CH_3)-), 130.41 (Ar_{MP}-$\underline{C}$H=CH-CH_3), 126.00 (Ar_{MP}-CH=$\underline{C}$H-CH_3), 122.92 ($\underline{C}_{Ar}$-CH_2-CH=), 122.60 ($C_{Ar,MP}$(6)-H), 122.09 (>$\underline{C}$=C(-CH_3)-), 118.22 ($C_{Ar,MP}$(5)-H), 116.73 ($\underline{C}_{Ar}$-CH_3), 109.50 ($C_{Ar,MP}$(3)-H), 106.33 ($\underline{C}_{Ar}$-COO-), 70.05 (Ar-$\underline{C}H_2$O-), 61.01 (Ar-O$\underline{C}H_3$), 55.73 (Ar_{MP}-O$\underline{C}H_3$), 34.61 (-$\underline{C}H_2$-COO-Ar_{MP}), 32.76 (-$\underline{C}H_2$-CH_2-), 22.62 (Ar-$\underline{C}H_2$-), 18.43 (Ar_{MP}-CH=CH-$\underline{C}H_3$), 16.16 (=C(-$\underline{C}H_3$)-), 11.58 (Ar-$\underline{C}H_3$). $R_{T,HPLC}$ = 17.097 min, purity: 99.60%; HRMS (*m/z*) calculated for $C_{27}H_{30}O_7$ [M — H]$^-$: 465.1913; found: 465.2001.

Sesamol mycophenolate (**P1**):

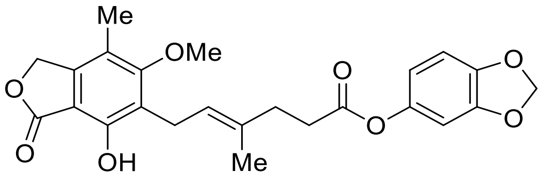

Eluent: dichloromethane. Obtained as a white precipitate with a 40% yield. R_F = 0.31 (dichloromethane). ^{1}H NMR ($CDCl_3$) δ 7.71 (s, 1H, Ar-OH), 6.73 (d, *J* = 8.3 Hz, 1H, $C_{Ar,P}$(3)-H), 6.47 – 6.39 (m, 2H, $C_{Ar,P}$(3)-H), 5.98 (s, 2H, -O-CH_2-O- (P)), 5.35 (t, *J* = 6.8 Hz, 1H, >C=CH-), 5.22 (s, 2H, Ar-CH_2O-), 3.78 (s, 3H, Ar-OCH_3), 3.44 (d, *J* = 6.9 Hz, 2H, Ar-CH_2-), 2.64 (t, *J* = 7.4 Hz, 2H, -CH_2-), 2.45 (t, *J* = 7.4 Hz, 2H, -CH_2-), 2.17 (s, 3H, Ar-CH_3), 1.88 (s, 3H, -CH_3). ^{13}C NMR ($CDCl_3$) δ 172.89 (-$\underline{C}$OO-Ar_P), 172.08 (-$\underline{C}$OO-), 163.62 ($\underline{C}_{Ar}$-OMe), 153.54 ($\underline{C}_{Ar}$-OH), 147.82 ($\underline{C}_{Ar,P}$-OOC-), 145.19 ($C_{Ar,P}$(4)-H), 144.95 ($C_{Ar,P}$(3)-H), 144.09 ($\underline{C}_{Ar}$-CH_2O-), 133.75 (>C=$\underline{C}$(-CH_3)-), 123.17 ($\underline{C}_{Ar}$-CH_2-CH=), 121.97 (>$\underline{C}$=C(-CH_3)-), 116.74 ($\underline{C}_{Ar}$-CH_3), 113.79 ($C_{Ar,P}$(6)-H), 107.83 ($C_{Ar,P}$(5)-H), 106.37 ($\underline{C}_{Ar}$-COO-), 103.68 ($C_{Ar,P}$(2)-H), 101.63 (-O-$\underline{C}H_2$-O- (P)), 70.05 (Ar-$\underline{C}H_2$O-), 61.00 (Ar-O$\underline{C}H_3$), 34.66 (-$\underline{C}H_2$-COO-Ar_P), 32.90 (-$\underline{C}H_2$-CH_2-), 22.60 (Ar-$\underline{C}H_2$-), 16.16 (=C(-$\underline{C}H_3$)-), 11.57 (Ar-$\underline{C}H_3$). $R_{T,HPLC}$ = 14.063 min, purity: 99.30%; HRMS (*m/z*) calculated for $C_{24}H_{24}O_8$ [M — H]$^-$: 439.1393; found: 439.1473.

Thymol mycophenolate (**P2**):

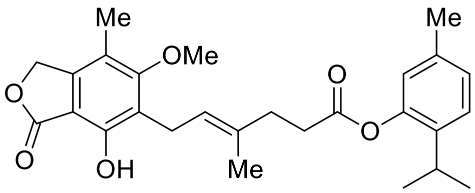

Eluent: toluene:chloroform, 3:1 (v/v). Obtained as a transparent oil with a 30% yield. R_F = 0.33 (dichloromethane). ^{1}H NMR ($CDCl_3$) δ 7.69 (s, 1H, Ar-OH), 7.17 (d, *J* = 7.9 Hz, 1H, $C_{Ar,P}$(3)-H), 7.01 (t, *J* = 8.3 Hz, 1H, $C_{Ar,P}$(3)-H), 6.73 (s, 1H, $C_{Ar,P}$(3)-H), 5.34 (dd, *J* = 7.0, 6.0 Hz, 1H, >C=CH-), 5.20 (s, 2H, Ar-CH_2O-), 3.77 (s, 3H, Ar-OCH_3), 3.43 (d, *J* = 6.9 Hz, 2H, Ar-CH_2-), 2.93 (hept, *J* = 6.9 Hz, 1H, -CH< (P)), 2.72 – 2.63 (m, 2H, -CH_2-), 2.46 (t, *J* = 7.7 Hz, 2H, -CH_2-), 2.28 (s, 3H, Ar_P-CH_3), 2.15 (s, 3H, Ar-CH_3), 1.87 (s, 3H, -CH_3), 1.16 (d, *J* = 6.9 Hz, 6H, >C(CH_3)$_2$ (P)). ^{13}C NMR ($CDCl_3$) δ 172.91 (-$\underline{C}$OO-Ar_P), 172.04 (-$\underline{C}$OO-), 163.67 ($\underline{C}_{Ar}$-OMe), 153.62 ($\underline{C}_{Ar}$-OH), 147.85 ($\underline{C_{Ar,P}}$-OOC-), 144.03 ($\underline{C}_{Ar}$-CH_2O-), 136.95 ($C_{Ar,P}$(2)-H), 136.45 ($C_{Ar,P}$(5)-H), 133.95 (>C=$\underline{C}$(-CH_3)-), 127.02 ($C_{Ar,P}$(3)-H), 126.32 ($C_{Ar,P}$(4)-H), 122.93 ($\underline{C}_{Ar}$-CH_2-CH=), 122.66 ($C_{Ar,P}$(6)-H), 122.01 (>$\underline{C}$=C(-CH_3)-), 116.73 ($\underline{C}_{Ar}$-CH_3), 106.37 ($\underline{C}_{Ar}$-COO-), 70.05 (Ar-$\underline{C}H_2$O-), 61.01 (Ar-O$\underline{C}H_3$), 34.63 (-$\underline{C}H_2$-COO-Ar_P), 33.01 (-$\underline{C}H_2$-CH_2-), 27.01 (Ar_P-$\underline{C}$H(CH_3)$_2$), 23.00 (2C, Ar_P-CH($\underline{C}H_3$)$_2$), 22.63 (Ar-$\underline{C}H_2$-), 20.79 (Ar_P-$\underline{C}H_3$), 16.22 (=C(-$\underline{C}H_3$)-), 11.58 (Ar-$\underline{C}H_3$). $R_{T,HPLC}$ = 18.736 min, purity: 100.00%; HRMS (*m/z*) calculated for $C_{27}H_{32}O_6$ [M — H]$^-$: 451.2121; found: 451.2208.

Bakuchiol mycophenolate (**P3**):

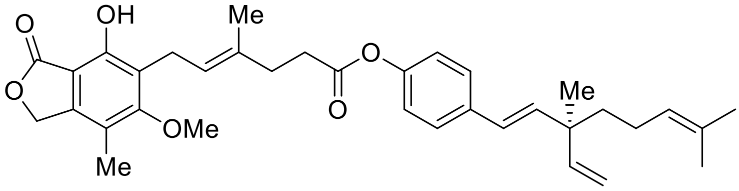

Eluent: toluene:chloroform, 1:1 (v/v). Obtained as a yellow oil with a 33% yield. R_F = 0.50 (dichloromethane). ^{1}H NMR ($CDCl_3$) δ 7.69 (s, 1H, Ar-OH), 7.29 (d, *J* = 8.6 Hz, 2H, $C_{Ar,P}$(3 and 5)-H), 6.91 (d, *J* = 8.6 Hz, 2H, $C_{Ar,P}$(2 and 6)-H), 6.28 (d, *J* = 16.2 Hz, 1H, Ar_P-C(1')H=), 6.14 (d, *J* = 16.2 Hz, 1H, >C(1')=C(2')H-), 5.87 (dd, *J* = 17.5, 10.7 Hz, 1H, >C(3')-C$\underline{H}$=), 5.34 (dd, *J* = 7.0, 5.9 Hz, 1H, >C=CH-), 5.19 (s, 2H, Ar-CH_2O-), 5.10 (t, *J* = 7.1 Hz, 1H, -C(6')H=), 5.03 (ddd, *J* = 18.7, 14.1, 1.2 Hz, 2H, >C(3')-CH=C$\underline{H_2}$), 3.75 (s, 3H, Ar-OCH_3), 3.42 (d, *J* = 6.9 Hz, 2H, Ar-CH_2-), 2.64 (t, *J* = 7.6 Hz, 2H, -CH_2-), 2.43 (t, *J* = 7.5 Hz, 2H, -CH_2-), 2.14 (s, 3H, Ar-CH_3), 1.94 (dd, *J* = 16.4, 7.6 Hz, 2H, -C(5')H_2-), 1.86 (s, 3H, -CH_3), 1.67 (s, 3H, =C(7')-CH_3), 1.57 (s, 3H, =C(7')-CH_3), 1.49 (ddd, *J* = 21.7, 9.9, 6.2 Hz, 2H, -C(4')H_2-), 1.20 (s, 3H, >C(3')-CH_3). ^{13}C NMR ($CDCl_3$) δ 172.90 (-$\underline{C}$OO-Ar_P), 171.81 (-$\underline{C}$OO-), 163.63 ($\underline{C}_{Ar}$-OMe), 153.59 ($\underline{C}_{Ar}$-OH), 149.54 ($\underline{C}_{Ar,P}$-OOC-), 145.62 (C(3')-$\underline{C}$H=CH_2), 144.01 ($\underline{C}_{Ar}$-CH_2O-), 138.11 ($\underline{C}_{Ar,P}$-CH=CH-R), 135.50 ($C_{Ar,P}$-$\underline{C}$H=CH-R), 133.81 (>C=$\underline{C}$(-CH_3)-), 131.38 (C(7')), 126.82 (2C, $C_{Ar,P}$(3 and 5)-H), 126.23 ($C_{Ar,P}$-CH=$\underline{C}$H-R), 124.66 (C(6')), 123.11 ($\underline{C}_{Ar}$-CH_2-CH=), 122.01 (>$\underline{C}$=C(-CH_3)-), 121.45 (2C, $C_{Ar,P}$(2 and 6)-H), 116.72 ($\underline{C}_{Ar}$-CH_3), 112.12 (C(3')-CH=$\underline{C}H_2$), 106.34 ($\underline{C}_{Ar}$-COO-), 70.03 (Ar-$\underline{C}H_2$O-), 61.00 (Ar-O$\underline{C}H_3$), 42.63 (C(4')), 41.18 (C(3')), 34.65 (-$\underline{C}H_2$-COO-Ar_P), 33.08 (-$\underline{C}H_2$-CH_2-), 25.70 (C(3')-$\underline{C}H_3$), 23.24 (C(7')-($\underline{C}H_3$)$_2$), 23.20 (C(5')), 22.62 (Ar-$\underline{C}H_2$-), 17.65 (C(7')-($\underline{C}H_3$)$_2$), 16.17 (=C(-$\underline{C}H_3$)-), 11.57 (Ar-$\underline{C}H_3$). $R_{T,HPLC}$ = 22.579 min, purity: 97.20%; HRMS (*m/z*) calculated for $C_{35}H_{42}O_6$ [M — H]$^-$: 557.2903; found: 557.3008.

(Methyl *o*-coumarate) mycophenolate (**P4**):

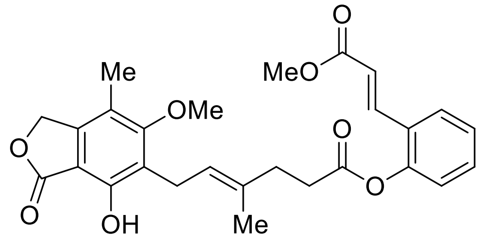

Eluent: dichloromethane. Obtained as a low-melting opal-white solid with a 24% yield. R_F = 0.25 (dichloromethane). ^{1}H NMR ($CDCl_3$) δ 7.74 (d, *J* = 16.1 Hz, 1H, Ar-CH=C< (P)), 7.70 (s, 1H, Ar-OH), 7.63 (d, *J* = 7.8 Hz, 1H, $C_{Ar,P}$(3)-H), 7.36 (t, *J* = 7.7 Hz, 1H, $C_{Ar,P}$(5)-H), 7.26 (t, *J* = 7.6 Hz, 1H, $C_{Ar,P}$(4)-H), 7.04 (d, *J* = 8.1 Hz, 1H, $C_{Ar,P}$(6)-H), 6.44 (d, *J* = 16.1 Hz, 1H, >C=CH- (P)), 5.37 (t, *J* = 6.5 Hz, 1H, >C=CH-), 5.22 (s, 2H, Ar-CH_2O-), 3.80 (d, *J* = 14.5 Hz, 6H, -$COOCH_3$ (P) and Ar-OCH_3), 3.45 (d, *J* = 6.9 Hz, 2H, Ar-CH_2-), 2.76 (t, *J* = 7.7 Hz, 2H, -CH_2-), 2.50 (t, *J* = 7.6 Hz, 2H, -CH_2-), 2.17 (s, 3H, Ar-CH_3), 1.90 (s, 3H, -CH_3). ^{13}C NMR ($CDCl_3$) δ 172.88 (-$\underline{C}$OO-Ar_P), 171.48 (-$\underline{C}$OO-), 167.08 (-$\underline{C}OOCH_3$ (P)), 163.63 ($\underline{C}_{Ar}$-OMe), 153.59 ($\underline{C}_{Ar}$-OH), 149.28 ($\underline{C_{Ar,P}}$-OOC-), 144.02 ($\underline{C}_{Ar}$-CH_2O-), 138.14 (Ar_P-$\underline{C}$H=CH-), 133.65 (>C=$\underline{C}$(-CH_3)-), 131.00 ($C_{Ar,P}$(5)-H), 127.46 ($C_{Ar,P}$(3)-H), 126.99 ($\underline{C_{Ar,P}}$-CH=CH-), 126.19 ($C_{Ar,P}$(4)-H), 123.23 ($\underline{C}_{Ar}$-CH_2-CH=), 123.05 ($C_{Ar,P}$(6)-H), 121.99 (>$\underline{C}$=C(-CH_3)-), 119.74 (Ar_P-$\underline{C}$H=CH-), 116.72 ($\underline{C}_{Ar}$-CH_3), 106.35 ($\underline{C}_{Ar}$-COO-), 70.03 (Ar-$\underline{C}H_2$O-), 61.00 (Ar-O$\underline{C}H_3$), 51.78 (-COO$\underline{C}H_3$ (P)), 34.54 (-$\underline{C}H_2$-COO-Ar_P), 32.92 (-$\underline{C}H_2$-CH_2-), 22.60 (Ar-$\underline{C}H_2$-), 16.18 (=C(-$\underline{C}H_3$)-), 11.57 (Ar-$\underline{C}H_3$). $R_{T,HPLC}$ = 15.151 min, purity: 98.50%; HRMS (*m/z*) calculated for $C_{27}H_{28}O_8$ [M — H]$^-$: 479.1706; found: 479.1799.

4-Hydroxybenzaldehyde mycophenolate (**P5**):

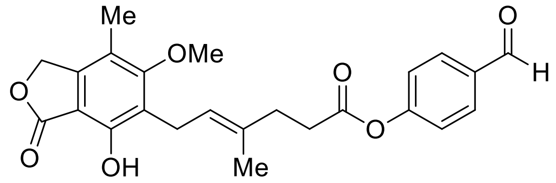

Eluent: petroleum ether:ethyl acetate, 3:1 (v/v). Obtained as a yellow oil with a 42% yield. R_F = 0.34 (dichloromethane). ^{1}H NMR ($CDCl_3$) δ 9.97 (s, 1H, Ar-CHO (P)), 7.84 (d, *J* = 8.5 Hz, 2H, $C_{Ar,P}$(3 and 5)-H), 7.72 (brs, 1H, Ar-OH), 7.16 (d, *J* = 8.5 Hz, 2H, $C_{Ar,P}$(2 and 6)-H), 5.35 (t, *J* = 6.7 Hz, 1H, >C=CH-), 5.20 (s, 2H, Ar-CH_2O-), 3.75 (s, 3H, Ar-OCH_3), 3.42 (d, *J* = 6.9 Hz, 2H, Ar-CH_2-), 2.69 (t, *J* = 7.5 Hz, 2H, -CH_2-), 2.45 (t, *J* = 7.5 Hz, 2H, -CH_2-), 2.14 (s, 3H, Ar-CH_3), 1.87 (s, 3H, -CH_3). ^{13}C NMR ($CDCl_3$) δ 190.93 (Ar_P-$\underline{C}$HO), 172.88 (-$\underline{C}$OO-Ar_P), 171.07 (-$\underline{C}$OO-), 163.59 ($\underline{C}_{Ar}$-OMe), 155.38 ($\underline{C_{Ar,P}}$-OOC-), 153.57 ($\underline{C}_{Ar}$-OH), 144.09 ($\underline{C}_{Ar}$-CH_2O-), 133.81 (>C=$\underline{C}$(-CH_3)-), 133.58 ($\underline{C_{Ar,P}}$-CHO), 131.06 (2C, $C_{Ar,P}$(3 and 5)-H), 123.30 ($\underline{C}_{Ar}$-CH_2-CH=), 122.28 (2C, $C_{Ar,P}$(2 and 6)-H), 121.90 (>$\underline{C}$=C(-CH_3)-), 116.77 ($\underline{C}_{Ar}$-CH_3), 106.37 ($\underline{C}_{Ar}$-COO-), 70.06 (Ar-$\underline{C}H_2$O-), 60.99 (Ar-O$\underline{C}H_3$), 34.57 (-$\underline{C}H_2$-COO-Ar_P), 33.04 (-$\underline{C}H_2$-CH_2-), 22.63 (Ar-$\underline{C}H_2$-), 16.13 (=C(-$\underline{C}H_3$)-), 11.58 (Ar-$\underline{C}H_3$). $R_{T,HPLC}$ = 13.015 min, purity: 97.80%; HRMS (*m/z*) calculated for $C_{24}H_{24}O_7$ [M — H]$^-$: 423.1444; found: 423.1524.

Dehydroframbinone mycophenolate (**P6**):

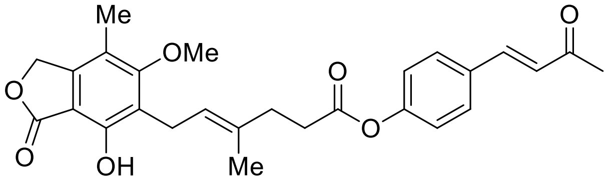

Eluent: petroleum ether:ethyl acetate, 3:1 (v/v). Obtained as a yellowish powder with a 55% yield. R_F = 0.19 (dichloromethane). ^{1}H NMR ($CDCl_3$) δ 7.68 (s, 1H, Ar-OH), 7.53 – 7.44 (m, 3H, $C_{Ar,P}$(3 and 5)-H and Ar–CH=C< (P)), 7.07 – 7.02 (m, 2H, $C_{Ar,P}$(2 and 6)-H), 6.66 (d, *J* = 16.2 Hz, 1H, –CH=C< (P)), 5.33 (td, *J* = 6.9, 1.2 Hz, 1H, >C=CH-), 5.19 (s, 2H, Ar-CH_2O-), 3.76 (s, 3H, Ar-OCH_3), 3.42 (d, *J* = 6.9 Hz, 2H, Ar-CH_2-), 2.66 (t, *J* = 7.5 Hz, 2H, -CH_2-), 2.44 (t, *J* = 7.5 Hz, 2H, -CH_2-), 2.38 (s, 3H, -(C=O)-CH_3 (P)), 2.14 (s, 3H, Ar-CH_3), 1.86 (s, 3H, -CH_3). ^{13}C NMR ($CDCl_3$) δ 198.26 (-CH=CH-($\underline{C}$=O)-CH_3 (P)), 172.90 (-$\underline{C}$OO-Ar_P), 171.44 (-$\underline{C}$OO-), 163.61 ($\underline{C}_{Ar}$-OMe), 153.57 ($\underline{C}_{Ar}$-OH), 152.29 ($\underline{C}_{Ar,P}$-OOC-), 144.03 ($\underline{C}_{Ar}$-CH_2O-), 142.26 ($C_{Ar,P}$-$\underline{C}$H=CH-), 133.69 (>C=$\underline{C}$(-CH_3)-), 131.95 ($\underline{C}_{Ar,P}$-CH=CH-), 129.27 (2C, $\underline{C}_{Ar,P}$(3 and 5)-H), 127.11 ($C_{Ar,P}$-CH=$\underline{C}$H-), 123.19 ($\underline{C}_{Ar}$-CH_2-CH=), 122.16 (2C, $\underline{C}_{Ar,P}$(2 and 6)-H), 121.96 (>$\underline{C}$=C(-CH_3)-), 116.75 ($\underline{C}_{Ar}$-CH_3), 106.35 ($\underline{C}_{Ar}$-COO-), 70.05 (Ar-$\underline{C}H_2$O-), 61.00 (Ar-O$\underline{C}H_3$), 34.56 (-$\underline{C}H_2$-COO-Ar_P), 33.05 (-$\underline{C}H_2$-CH_2-), 27.63 (=CH-(C=O)-$\underline{C}H_3$ (P)), 22.62 (Ar-$\underline{C}H_2$-), 16.17 (=C(-$\underline{C}H_3$)-), 11.59 (Ar-$\underline{C}H_3$). $R_{T,HPLC}$ = 14.021 min, purity: 99.80%; HRMS (*m/z*) calculated for $C_{27}H_{28}O_7$ [M — H]$^-$: 463.1757; found: 463.1759.

Raspberry ketone mycophenolate (**P7**):

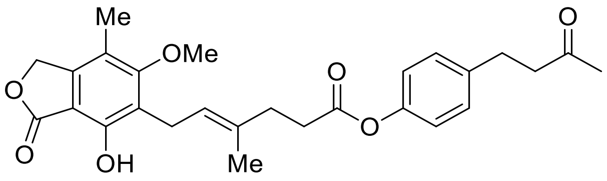

Eluent: toluene:chloroform, 1:1 (v/v). Obtained as a dense yellowish solid with a 53% yield. R_F = 0.28 (dichloromethane). ^{1}H NMR ($CDCl_3$) δ 7.68 (s, 1H, Ar-OH), 7.12 (d, *J* = 8.4 Hz, 2H, $C_{Ar,P}$(3 and 5)-H), 6.88 (d, *J* = 8.4 Hz, 2H, $C_{Ar,P}$(2 and 6)-H), 5.33 (t, *J* = 6.6 Hz, 1H, >C=CH-), 5.20 (s, 2H, Ar-CH_2O-), 3.75 (s, 3H, Ar-OCH_3), 3.42 (d, *J* = 6.9 Hz, 2H, Ar-CH_2-), 2.86 (t, *J* = 7.5 Hz, 2H, Ar-CH_2- (P)), 2.74 (t, *J* = 7.5 Hz, 2H, -CH_2-CO- (P)), 2.63 (t, *J* = 7.5 Hz, 2H, -CH_2-COO-), 2.43 (t, *J* = 7.5 Hz, 2H, -CH_2-), 2.14 (d, *J* = 2.5 Hz, 6H, Ar-CH_3 and -(C=O)-CH_3 (P)), 1.86 (s, 3H, -CH_3). ^{13}C NMR ($CDCl_3$) δ 207.76 (-CH_2-($\underline{C}$=O)-CH_3 (P)), 172.90 (-$\underline{C}$OO-), 171.91 (-$\underline{C}$OO-Ar_P), 163.64 ($\underline{C}_{Ar}$-OMe), 153.58 ($\underline{C}_{Ar}$-OH), 148.91 ($\underline{C}_{Ar,P}$-OOC-), 144.03 ($\underline{C}_{Ar}$-CH_2O-), 138.44 ($\underline{C}_{Ar,P}$-CH_2-CH_2-), 133.82 (>C=$\underline{C}$(-CH_3)-), 129.16 (2C, $C_{Ar,P}$(3 and 5)-H), 123.08 (>$\underline{C}$=C(-CH_3)-), 122.02 ($\underline{C}_{Ar}$-CH_2-CH=), 121.45 (2C, $C_{Ar,P}$(2 and 6)-H), 116.76 ($\underline{C}_{Ar}$-CH_3), 106.34 ($\underline{C}_{Ar}$-COO-), 70.06 (Ar-$\underline{C}H_2$O-), 61.00 (Ar-O$\underline{C}H_3$), 45.05 (-$\underline{C}H_2$-(C=O)-CH_3 (P)), 34.62 (-$\underline{C}H_2$-CH_2-), 33.04 (-$\underline{C}H_2$-COO-Ar_P), 30.10 (-CH_2-(C=O)-$\underline{C}H_3$ (P)), 28.97 (Ar_P-$\underline{C}H_2$-CH_2-(C=O)-), 22.61 (Ar-$\underline{C}H_2$-), 16.17 (=C(-$\underline{C}H_3$)-), 11.58 (Ar-$\underline{C}H_3$). $R_{T,HPLC}$ = 14.148 min, purity: 98.90%; HRMS (*m/z*) calculated for $C_{27}H_{30}O_7$ [M — H]$^-$: 465.1913; found: 465.2003.

Biological evaluation — AsPC-1 cell line

Cell culture

AsPC-1 pancreatic cancer cells (purchased from the American Type Culture Collection, ATCC, Manassas, VA, USA) were maintained in RPMI 1640 medium (Sigma-Aldrich, St Louis, MO, USA), supplemented with heat-inactivated 10% fetal bovine serum (FBS; Biowest, Riverside, MO, USA), 100 µg/mL streptomycin, and 100 units/mL penicillin. Cells were incubated in a humidified atmosphere of 5% CO_2 at 37°C. All experiments were performed on cells in a logarithmic growth phase.

Cell viability assay

To investigate the effect of the tested compounds on cell viability, a colorimetric analysis was performed using MTT (3-(4,5-Dimethyl-2-thiazolyl)-2,5-diphenyl-2*H*-tetrazolium bromide, Sigma-Aldrich, St Louis, MO, USA) based on the reduction of this yellow salt in metabolically active cells to purple formazan crystals. Cells were seeded onto 96-well plates at $2 \cdot 10^3$ cells/well and after 24 h incubation for cell attachment, cells were treated with various concentrations of tested compounds and gemcitabine (positive control) up to 50 µM. After 72 hours of incubation, 20 µL MTT at a concentration of 4 mg/mL was added to each well for 3-4 hours. The culture medium was then aspirated and formazan crystals were dissolved in DMSO. The absorbance of the solutions was measured at 540 nm using an iMark Microplate Absorbance Reader (Bio-Rad, Hercules, CA, USA). The drug concentrations required to inhibit cell growth by 50% (IC_{50}) compared to untreated control cells were determined from curves plotting survival as a function of dose. Results were obtained from at least three independent experiments.

Statistical analysis

Statistical analysis of MTT cytotoxicity assay results was performed using GraphPad Prism 5.0 by two-tailed unpaired Student's t-test. Differences $p < 0.05$ between MPA and its derivatives and gemcitabine were considered statistically significant according to the following criteria: * $p < 0.05$, ** $p < 0.01$, and *** $p < 0.001$.

Biological evaluation — PBMC and T-Jurkat cells

Research materials

The T-Jurkat cell line (Clone E6-1, ATCC TIB-152), derived from a human acute T-cell leukemia, was obtained from the cell bank of the Department of Medical Immunology at the Medical University of Gdańsk. Before experimentation, the cells were thawed and cultured for about 7 days to reach the logarithmic phase of growth. The T-Jurkat cell line was suspended in a complete RPMI medium, which included RPMI-1640, 10% FBS, penicillin (100 U/ml), and streptomycin (100 mg/ml).

Human peripheral blood mononuclear cells (PBMCs) were isolated from buffy coats provided by anonymous, healthy volunteers with an average age of 35±4 years during routine transfusion procedure. These samples were received by the Authors from the Regional Centre for Blood Donation and Treatment in Gdańsk (Poland). The Authors did not work with volunteers and used granted samples solely for PBMCs isolation. PBMCs were suspended in a complete X-VIVO medium, consisting of X-VIVO20, 10% heat-inactivated human AB serum, penicillin (100 U/ml), and streptomycin (100 mg/ml).

MPA derivatives preparation

To achieve the desired concentrations of the tested compounds, the substances were first dissolved in DMSO to reach a final concentration of 0.01 M. The solutions were then diluted in the appropriate additive-free medium (RPMI-1640 for T-Jurkat cells and X-VIVO20 for PBMCs) to obtain the following concentrations: 100, 50, 20, 10, 2, 0.2, and 0.02 μM.

PBMCs isolation

Peripheral blood mononuclear cells were isolated by gradient centrifugation, as described previously [47]. Buffy coats were diluted with phosphate-buffered saline (PBS, pH = 7.4, ThermoFisher Scientific) in a 1:1 ratio, layered at Ficoll-Paque™ PLUS (VWR International), and centrifuged at 800 × g for 20 minutes. The PBMC layer was collected and washed twice with PBS (1st wash: 600 × g for 10 minutes; 2nd wash: 600 × g for 5 minutes). Cell amount and viability were assessed using the Bio-Rad TC20™ automated cell counter combined with trypan blue staining.

XTT dye-based *in vitro* cytotoxicity test

The cytotoxic effects of mycophenolic acid (MPA) derivatives were assessed using an in vitro XTT-based assay (Roche). PBMCs in complete X-VIVO20 medium and T-Jurkat cells in complete RPMI-1640 medium were seeded into 96-well plates at a concentration of $5 \cdot 10^5$ cells/50 μl per well. 50 μl of tested compounds were added to the experimental wells to achieve final concentrations of 50, 25, 10, 5, 1, 0.1, and 0.01 μM. The background control consisted of the medium without cells, while untreated cells served as the positive control. After incubating for 48 hours for T-Jurkat cells and 96 hours for PBMCs in a humidified atmosphere with 5% CO_2 at 37°C, 50 μl of freshly prepared XTT reagent was added to all wells according to the manufacturer's instructions. The plates were then incubated for an additional 24 hours under the same conditions. The conversion of the water-soluble yellow XTT tetrazolium salt to orange formazan was quantified by measuring the optical density (OD) at 450 nm using an Agilent BioTek Epoch Microplate Spectrophotometer (Agilent Technologies), with a reference wavelength of 690 nm. The results were expressed as IC_{50} values for compounds that

inhibited cell viability by more than 50%. Each experiment was conducted in triplicate, and the final concentration of DMSO in the culture did not affect cell viability.

***VPD4*50 dye-based *in vitro* cell proliferation test**

PBMCs and T-Jurkat cells were washed twice with warm PBS (37°C), re-suspended in fresh PBS at a final concentration of $10\text{-}30 \cdot 10^6$ cells/ml, and labelled with 1 µl of VPD450 (BD Biosciences) at 37°C for 15 minutes, with vortexing every 5 minutes to ensure uniform staining [48, 49]. Subsequently, cells were washed with PBS, then with the complete medium (X-VIVO20 for PBMCs and RPMI-1640 for T-Jurkat cells), next re-suspended in fresh complete medium and seeded into 96-well plates at a concentration of $1 \cdot 10^6$ cells per well. PBMCs were additionally stimulated with magnetic beads coated with anti-CD3 and anti-CD28 antibodies (Invitrogen) at a 2:1 cell-to-bead ratio. To the experimental wells, 100 µl of the MPA derivatives were added to achieve final concentrations of 50, 25, 10, 5, 1, 0.1, and 0.01 µM. In parallel, control wells containing stained but unstimulated cells and stained, stimulated cells were prepared at the same concentration. Cell proliferation was analyzed using an LSRFortessa flow cytometer (BD Biosciences) and Kaluza C Flow Cytometry Analysis Software (Beckman Coulter). The data were expressed as EC_{50} values for compounds that inhibited more than 50% of cell proliferation.

Enzyme inhibition in the presence of guanosine source (GMP test)

PBMCs were suspended in a 15 mL centrifuge tube at a concentration of $10\text{-}30 \times 10^6$ cells in 1 mL of PBS, then, 2 µL of 1 mM VPD 450 dye solution was added, incubated for 15 min at 37°C, washed with a 9-fold volume of warm PBS and centrifuged at 400 x g for 5 min. Then, the cells were suspended in 10 mL of X-VIVO medium with 10% FBS and centrifuged again at 400 x g for 5 min. After suspending the cells in the complete medium, the staining level was checked using flow cytometry. The cells thus obtained were stimulated with anti-CD3/anti-CD28 antibodies as described previously (see *VPD4*50 dye-based *in vitro* cell proliferation test). To perform the GMP test, a proper amount of GMP was added to each well to reach its final concentration of 50 µM, and then incubated for 72 hours. Proliferative activity was tested in the presence of various concentrations of compounds (50; 25; 10; 1 and 0.1 µM; MPA and selected amide derivatives) or without their addition. Cell proliferation was assessed using flow cytometry again.

Statistical analysis

Statistical analysis was conducted using GraphPad Prism 9 (GraphPad Software Inc). Differences between MMF, MPA and its new derivatives were assessed using a two-tailed unpaired Student's t-test, with statistical significance defined as $p < 0.05$. EC_{50} and IC_{50} values were determined by fitting data to a four-parameter non-linear regression model in GraphPad Prism 9. Statistical significance was evaluated using the t-test.

NMR: Mycophenolic acid (**MPA**):

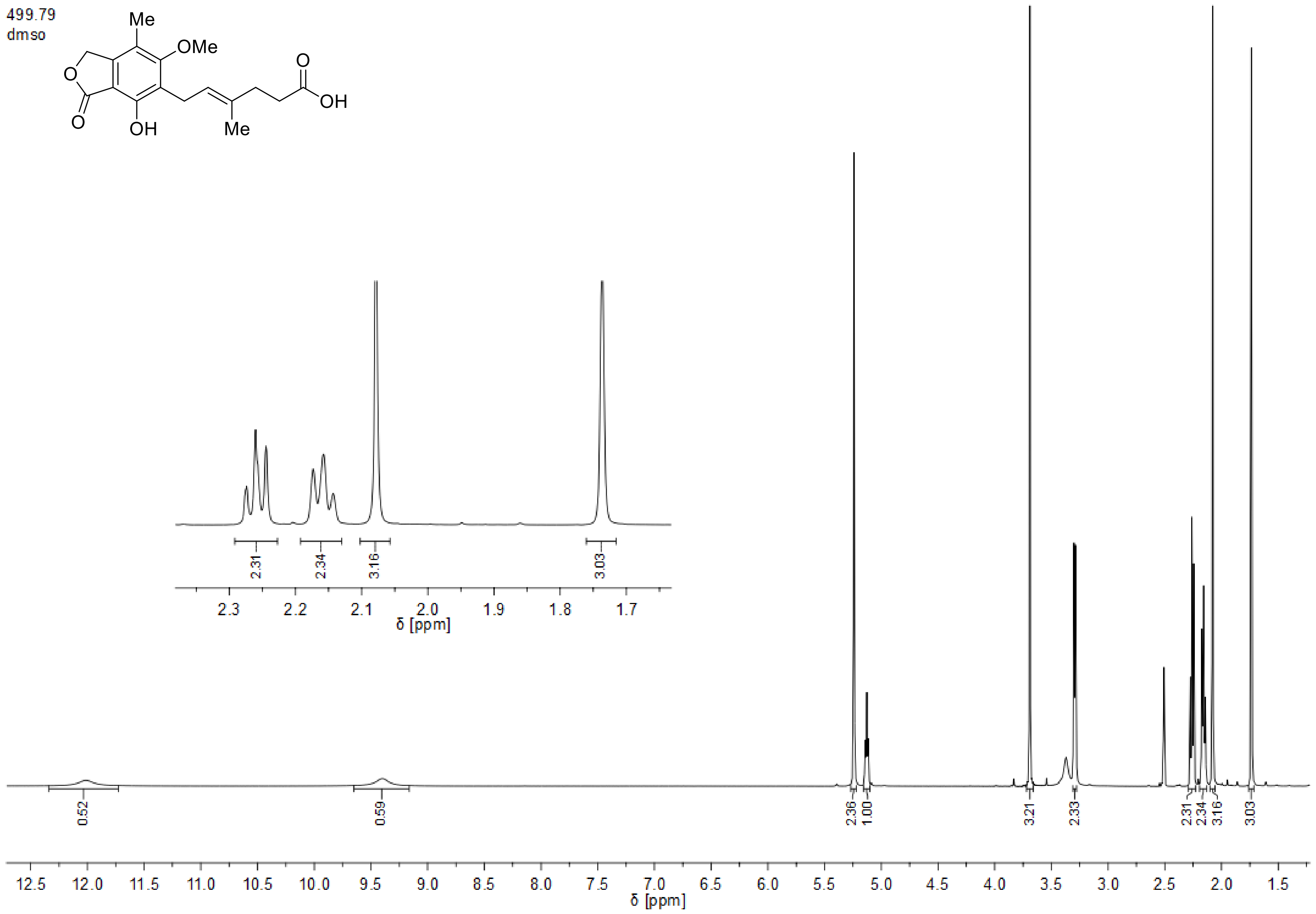

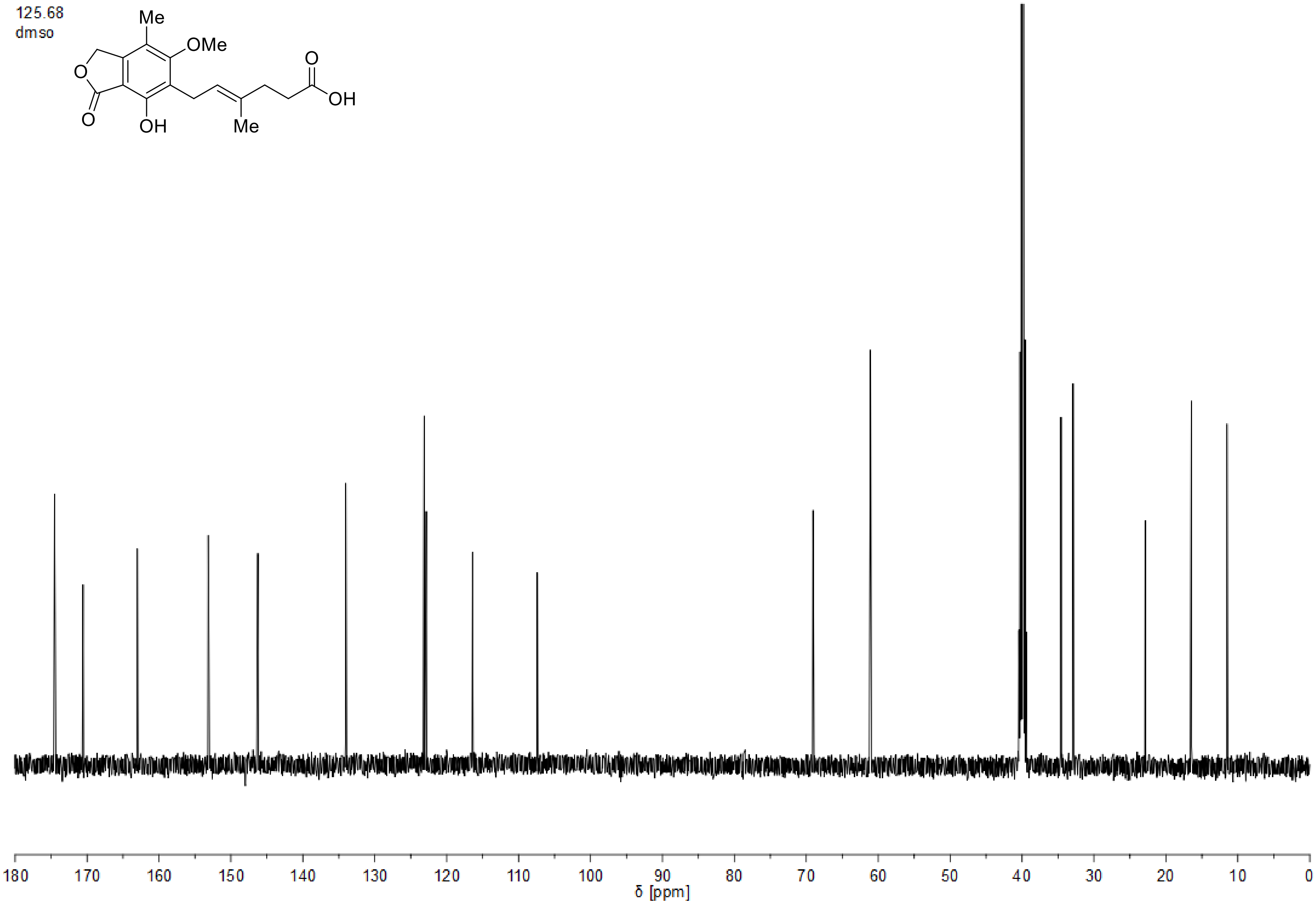
125.68
dmso
Me
OMe
O
O
OH
O
OH
Me
180
170
160
150
140
130
120
110
100
90
80
70
60
50
40
30
20
10
0
δ [ppm]

NMR: Mycophenolate mofetil (**MMF**):

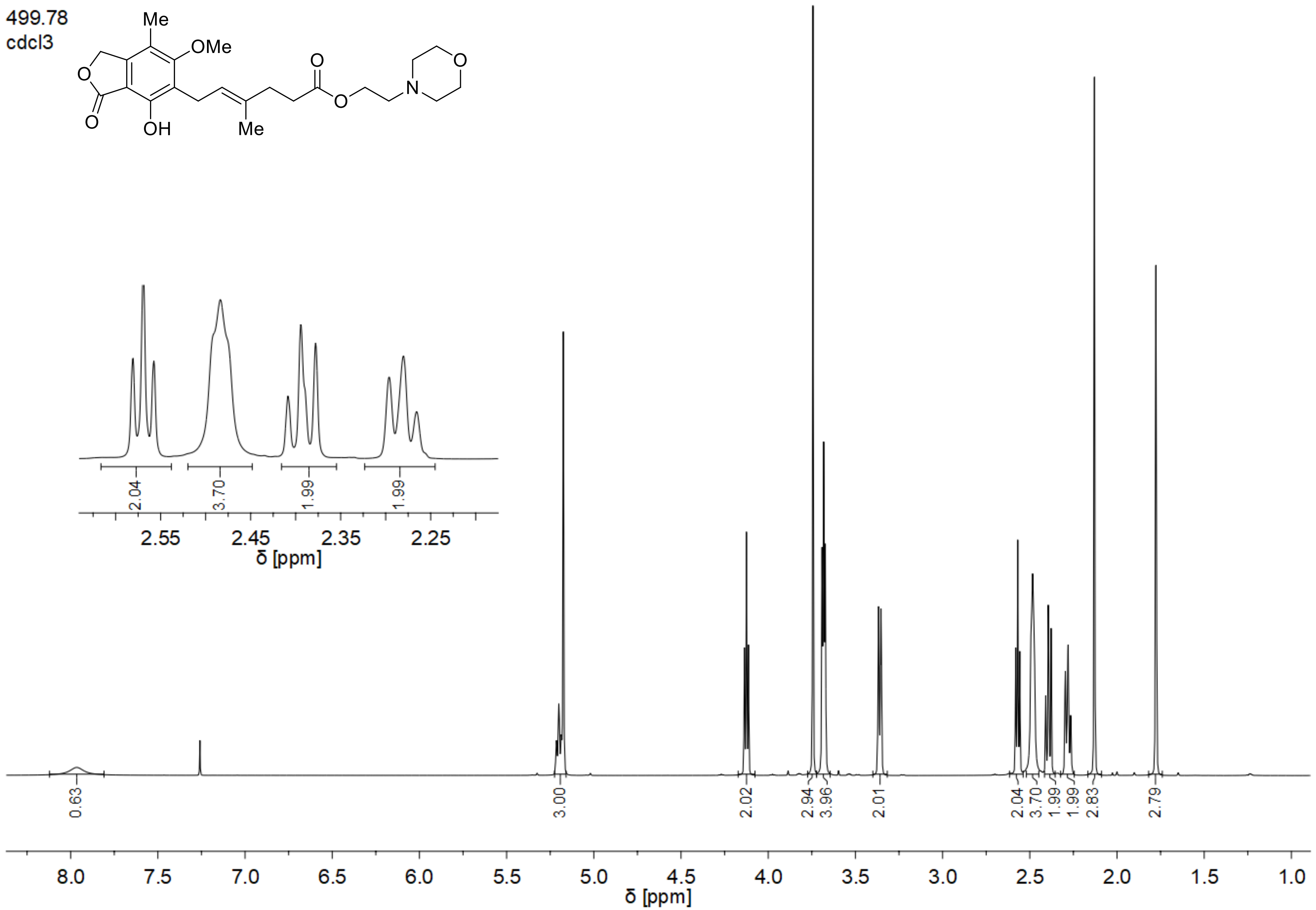

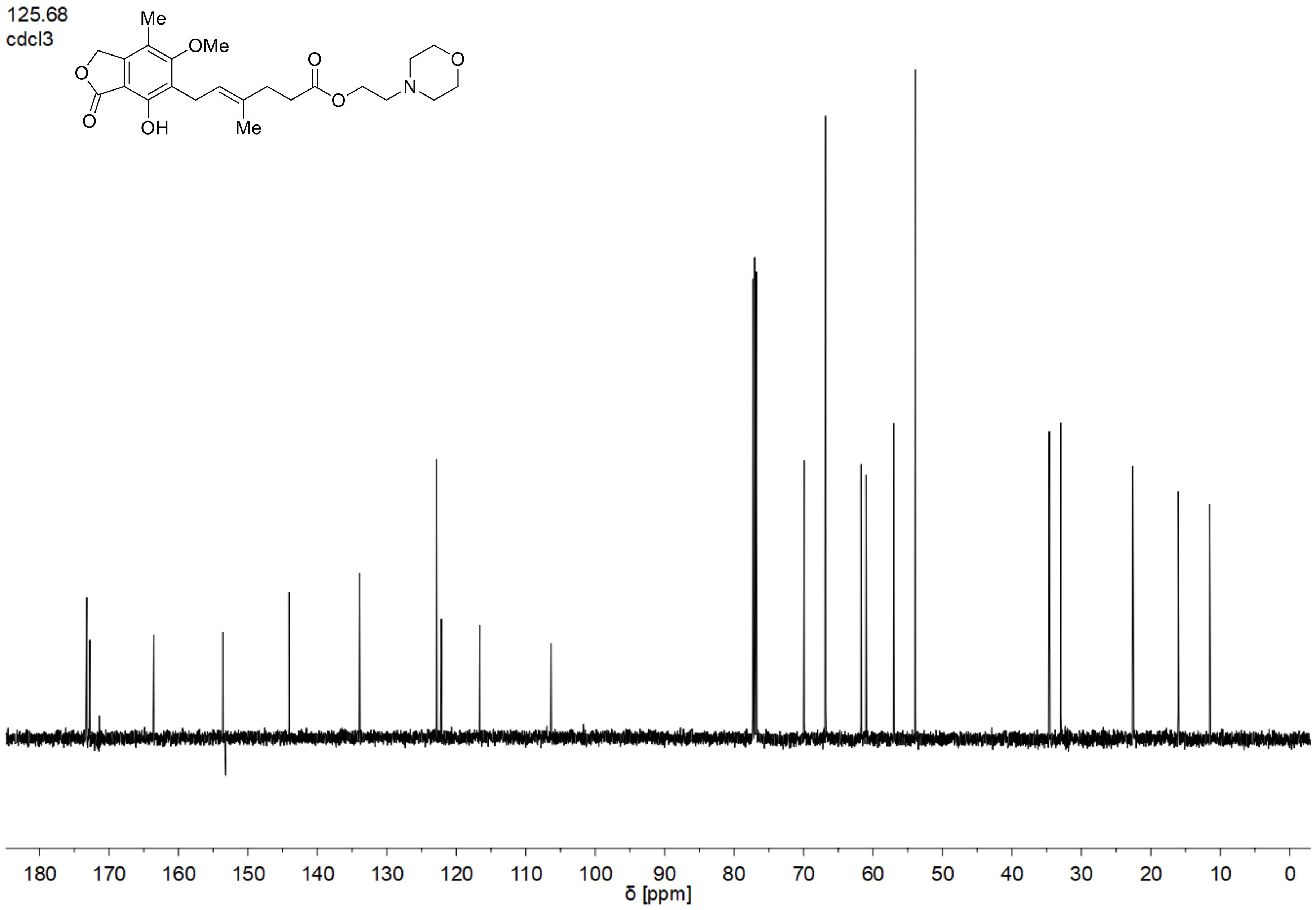
125.68
cdcl3
Me
OMe
O
O
O
O
N
O
OH
Me
O
180 170 160 150 140 130 120 110 100 90 80 70 60 50 40 30 20 10 0
δ [ppm]

NMR: Guaiacol mycophenolate (**MP1**):

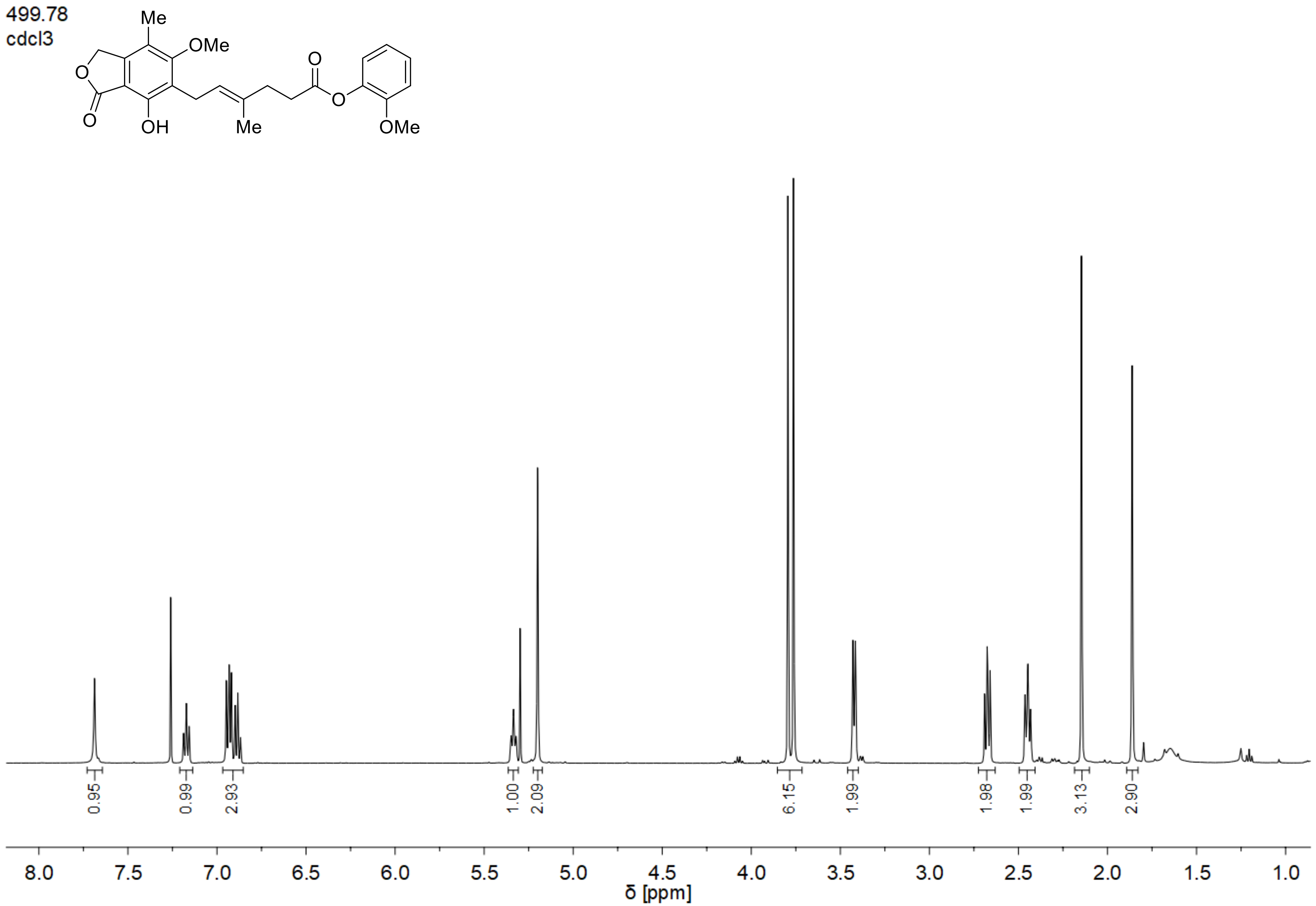

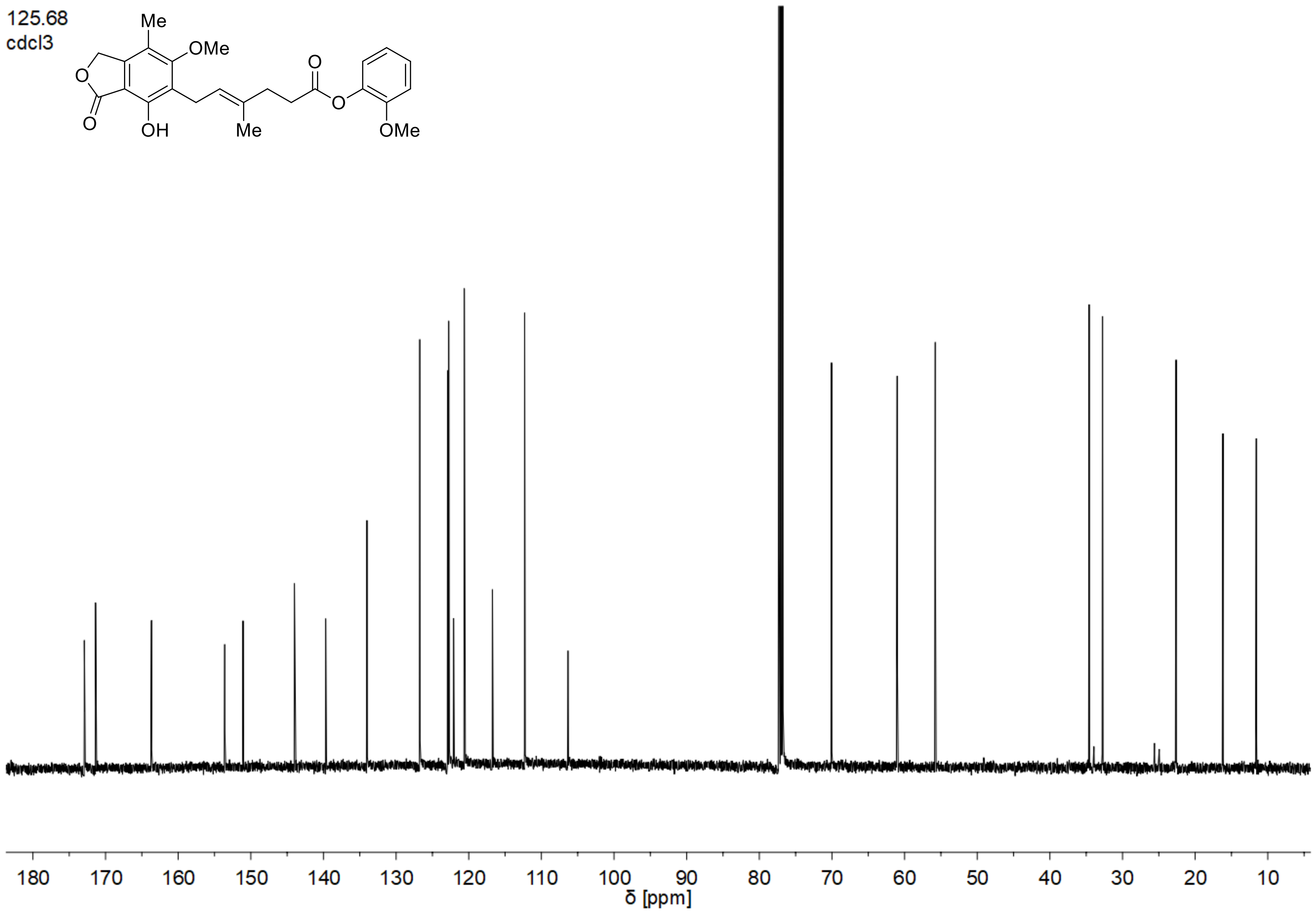
125.68
cdcl3
Me
OMe
O
O
O
OH
Me
O
OMe
180
170
160
150
140
130
120
110
100
90
80
70
60
50
40
30
20
10
δ [ppm]

NMR: Syringol mycophenolate (**MP2**):

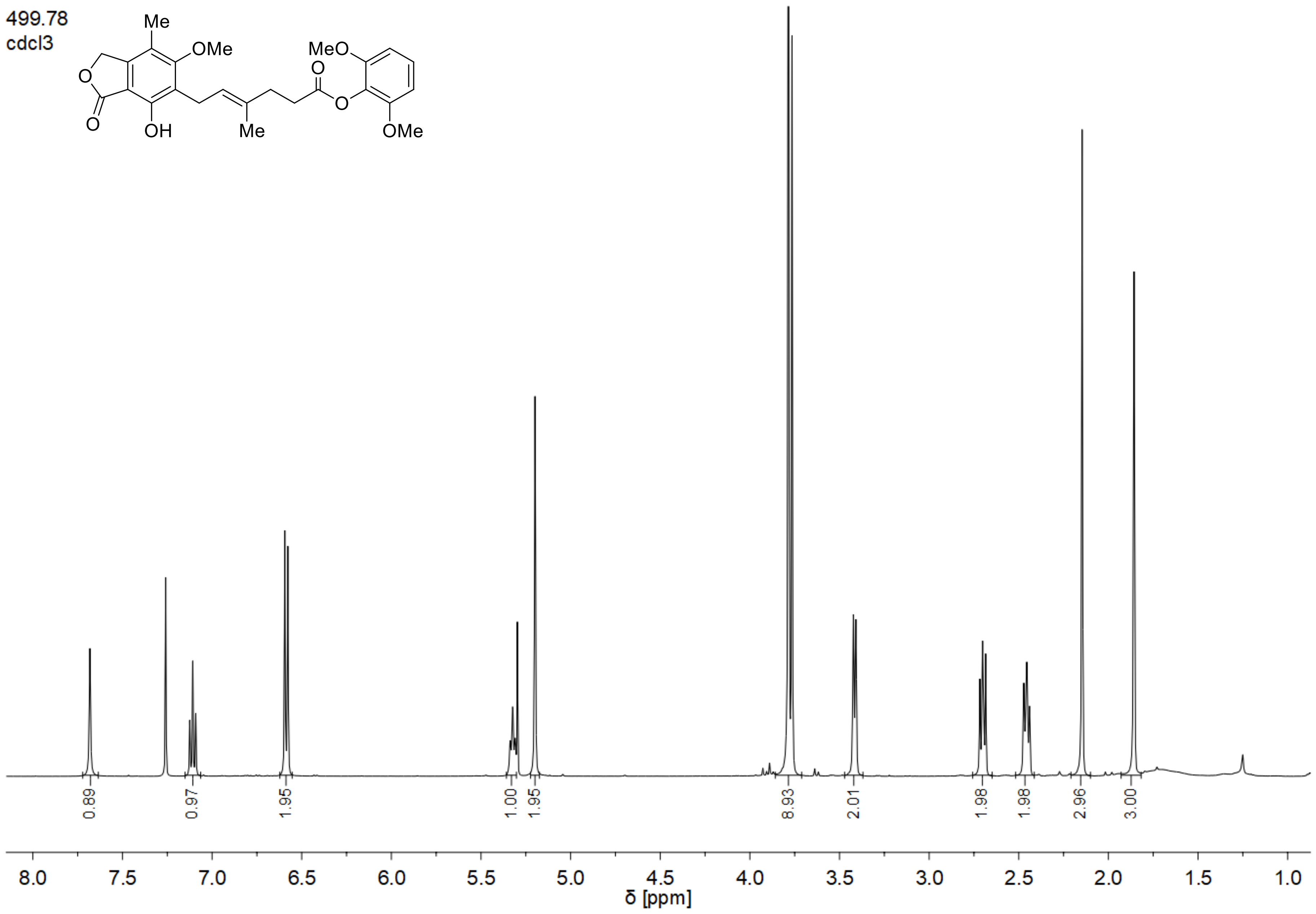

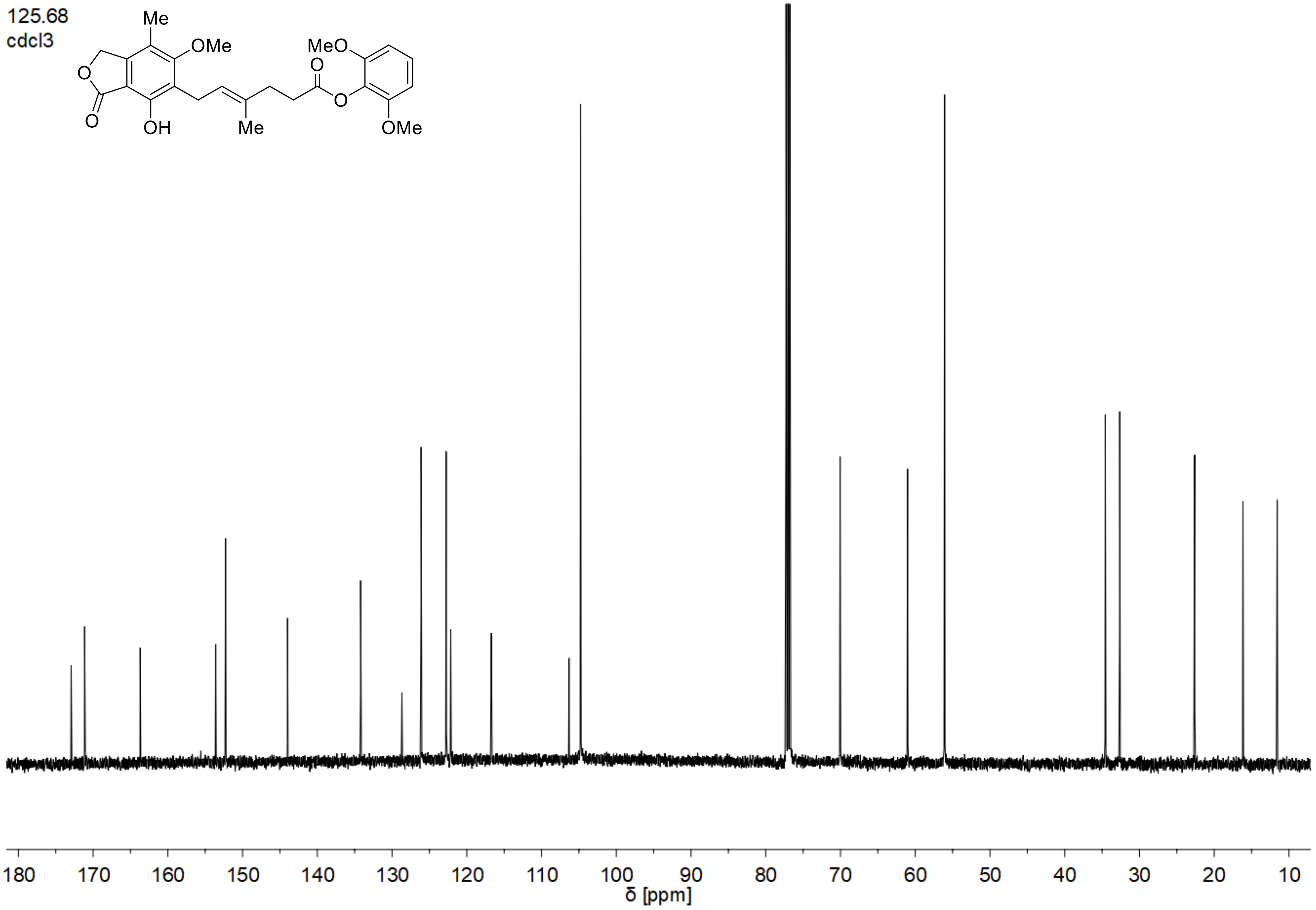

125.68
cdcl3
Me
OMe
MeO
O
O
O
O
OH
Me
O
OMe
180
170
160
150
140
130
120
110
100
90
80
70
60
50
40
30
20
10
δ [ppm]

NMR: Syringaldehyde mycophenolate (**MP3**):

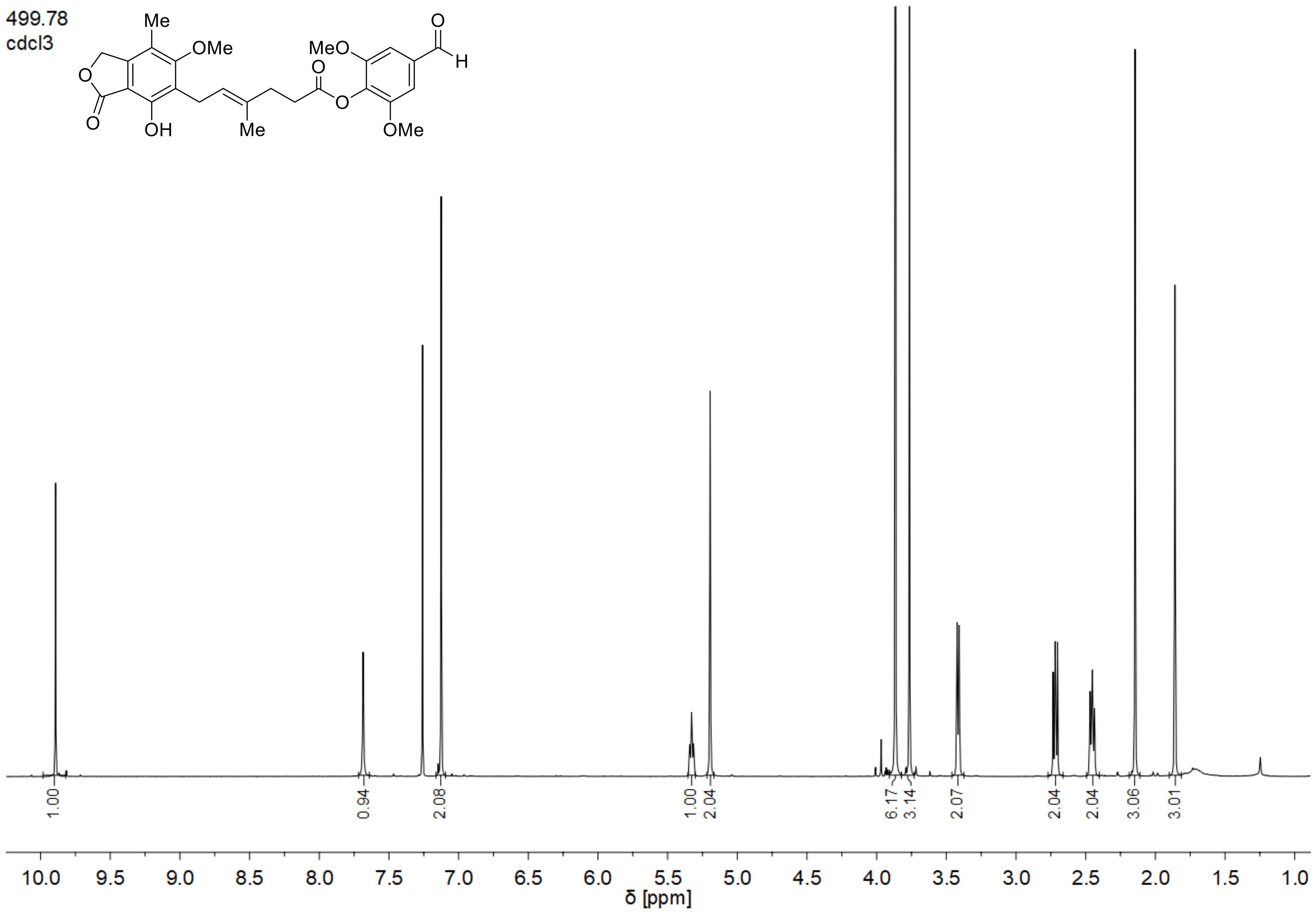

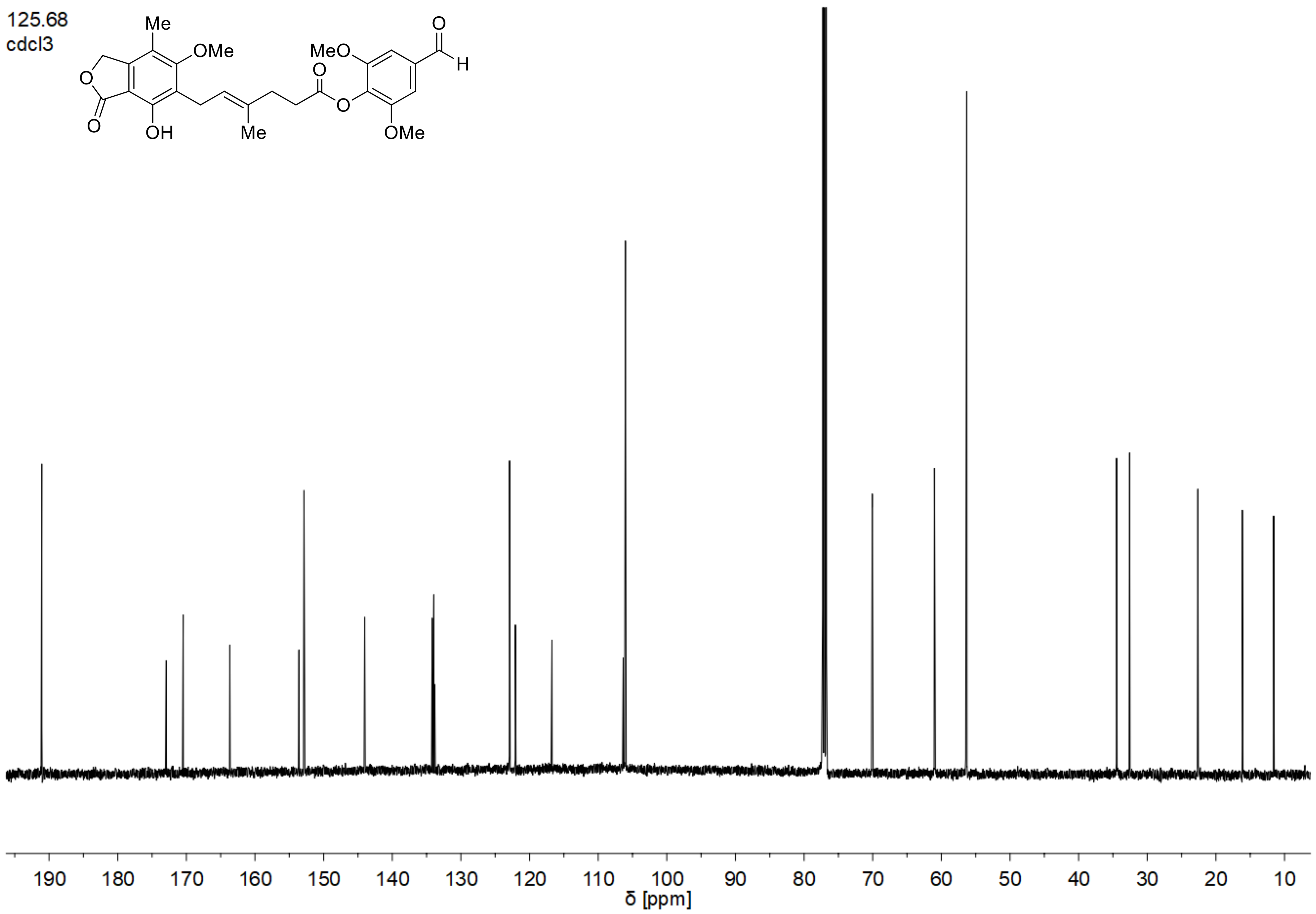
125.68
cdcl3
Me
OMe
MeO
O
H
O
O
O
O
OH
Me
OMe
190 180 170 160 150 140 130 120 110 100 90 80 70 60 50 40 30 20 10
δ [ppm]

NMR: Vanillin mycophenolate (**MP4**):

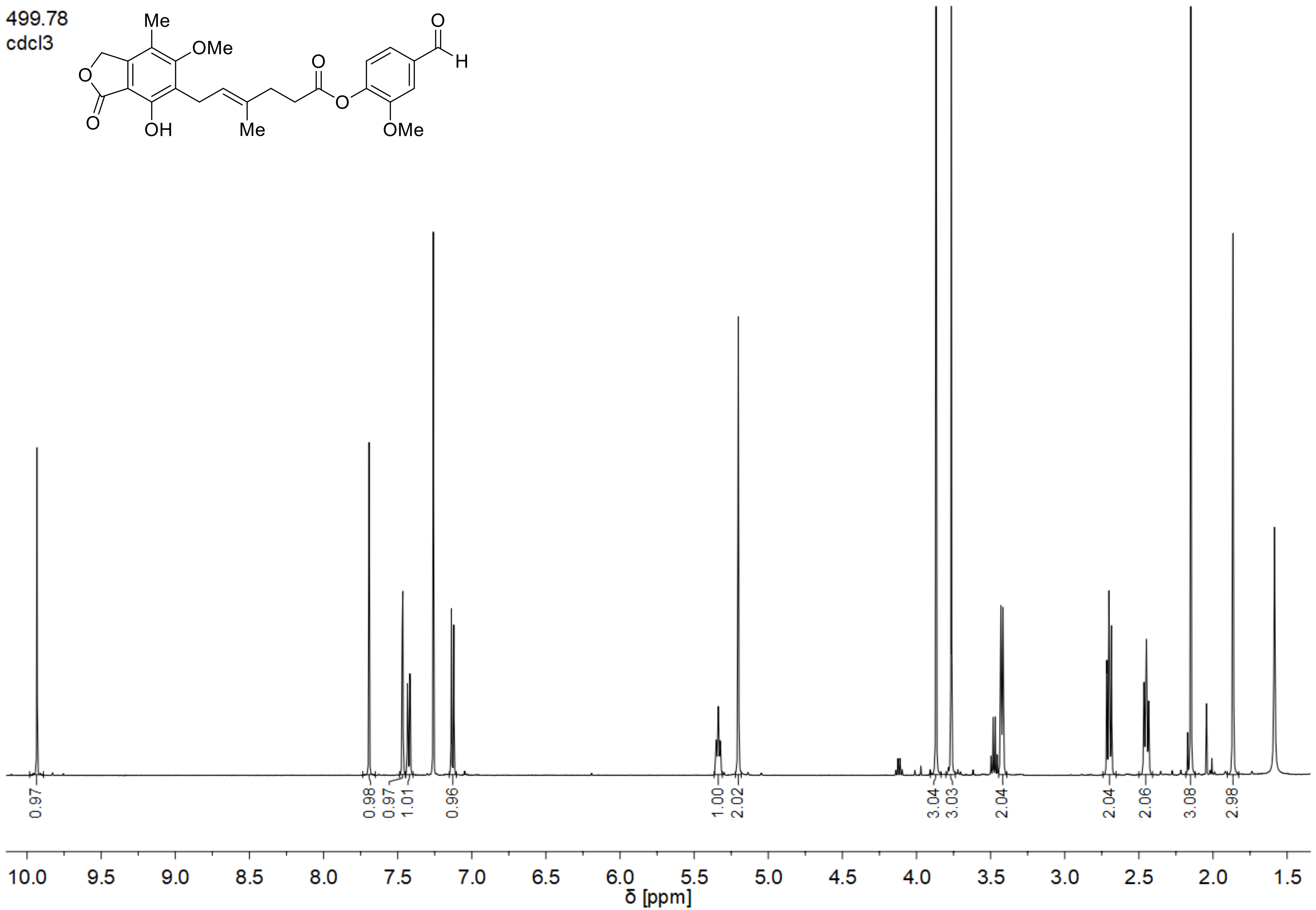

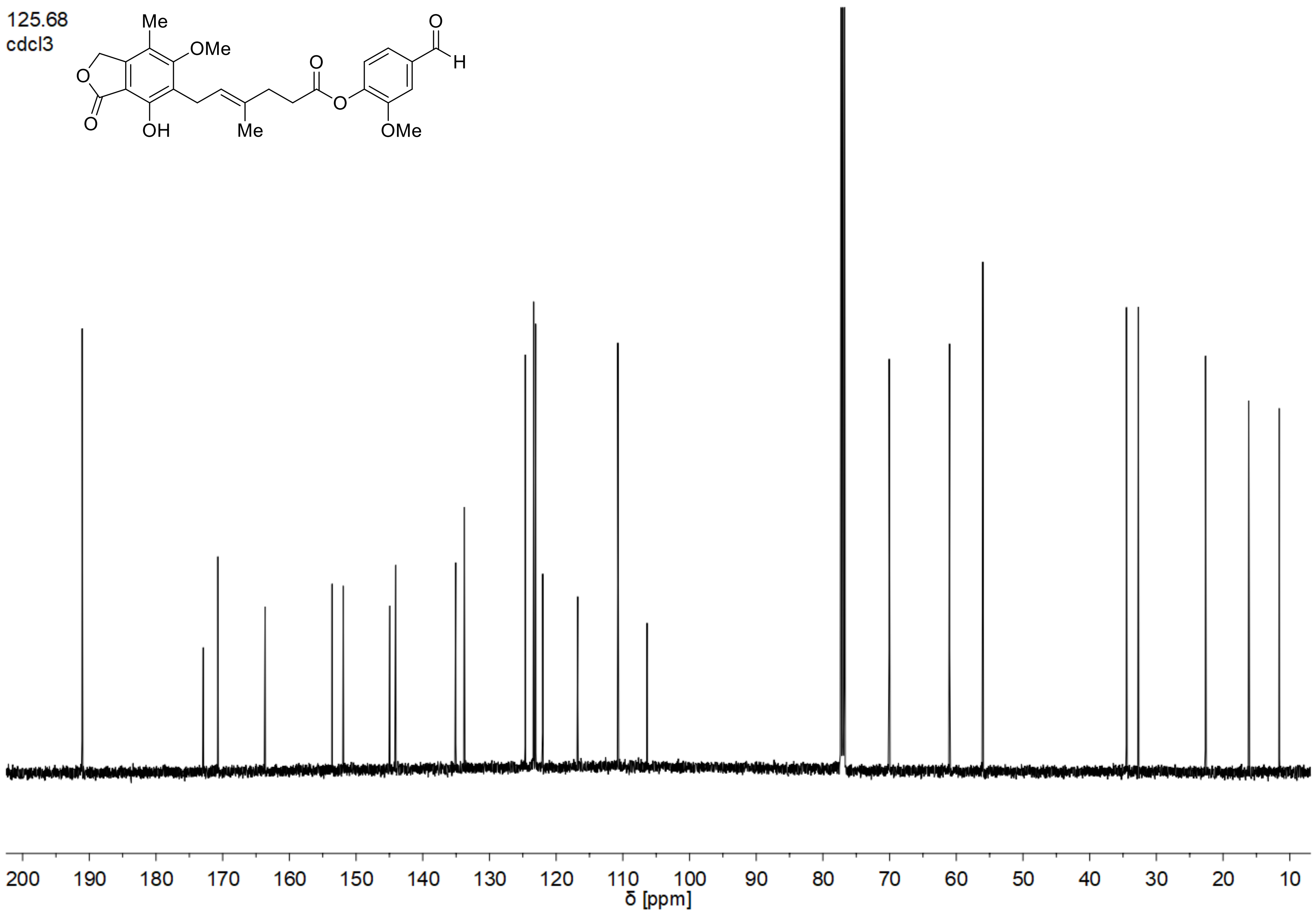
125.68
cdcl3
Me
OMe
O
H
OH
Me
OMe
200 190 180 170 160 150 140 130 120 110 100 90 80 70 60 50 40 30 20 10
δ [ppm]

NMR: Dehydrozingerone mycophenolate (**MP5**):

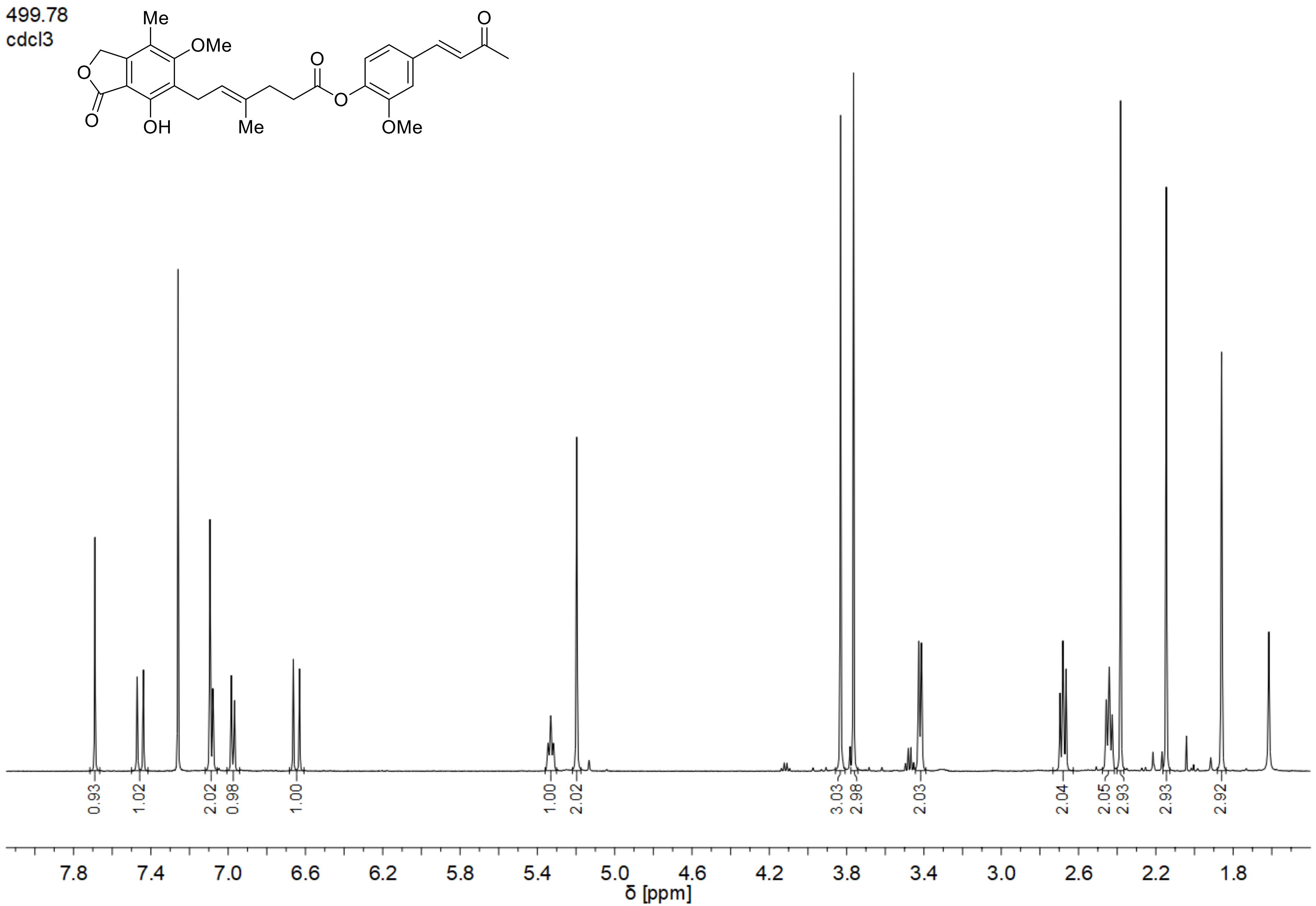

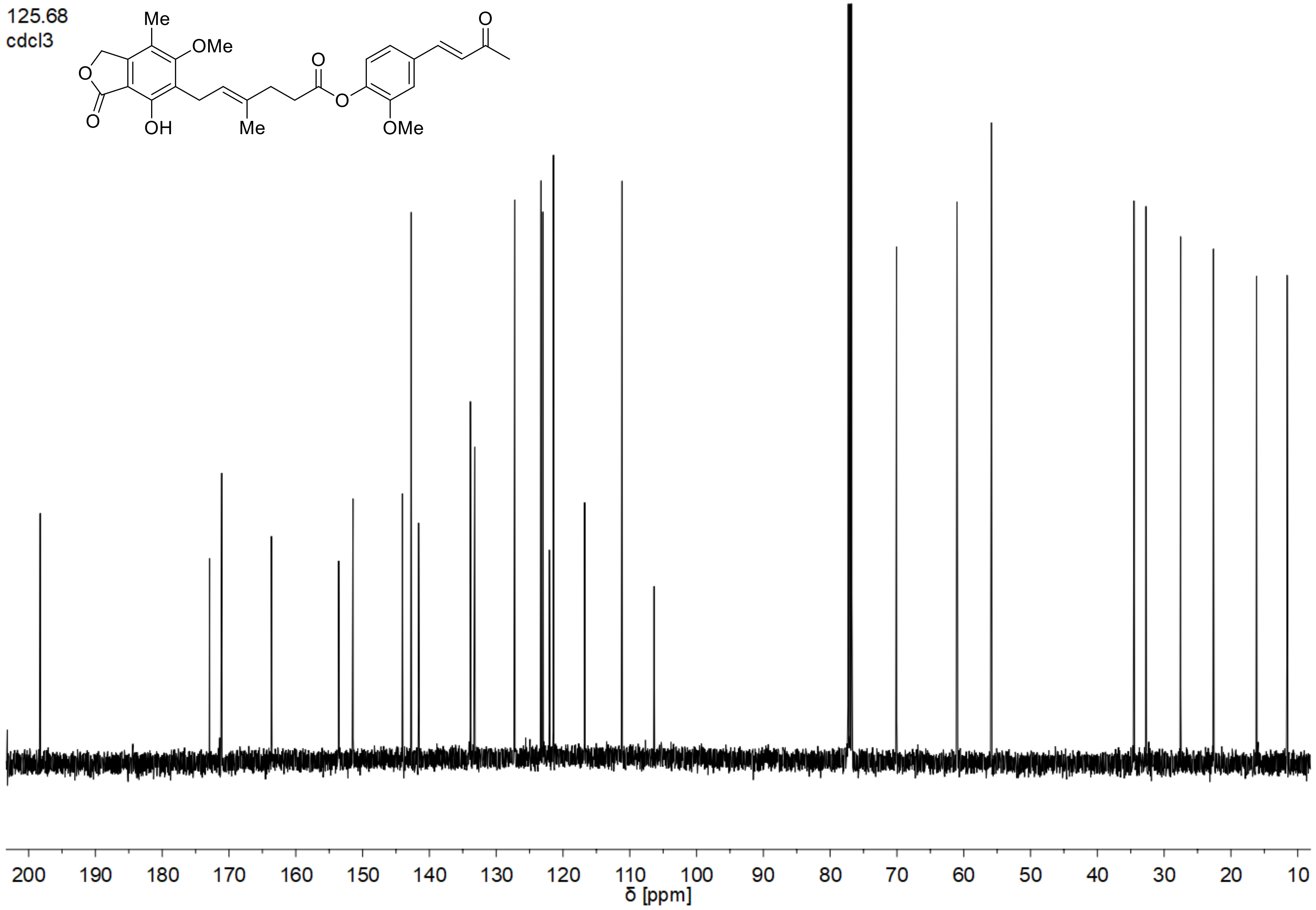
125.68
cdcl3
Me
OMe
O
O
O
O
O
OH
Me
OMe
200 190 180 170 160 150 140 130 120 110 100 90 80 70 60 50 40 30 20 10
δ [ppm]

NMR: Zingerone mycophenolate (**MP6**):

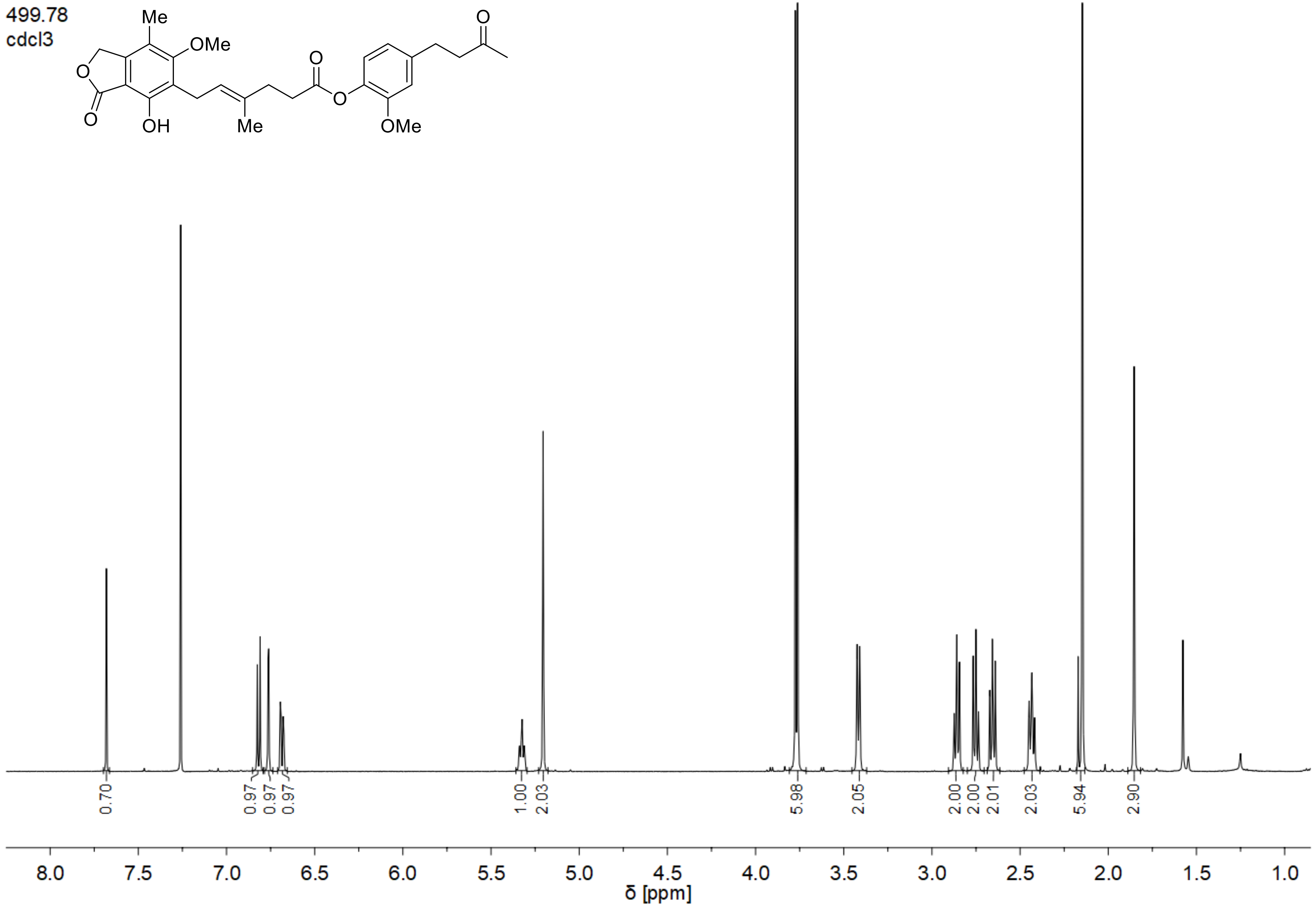

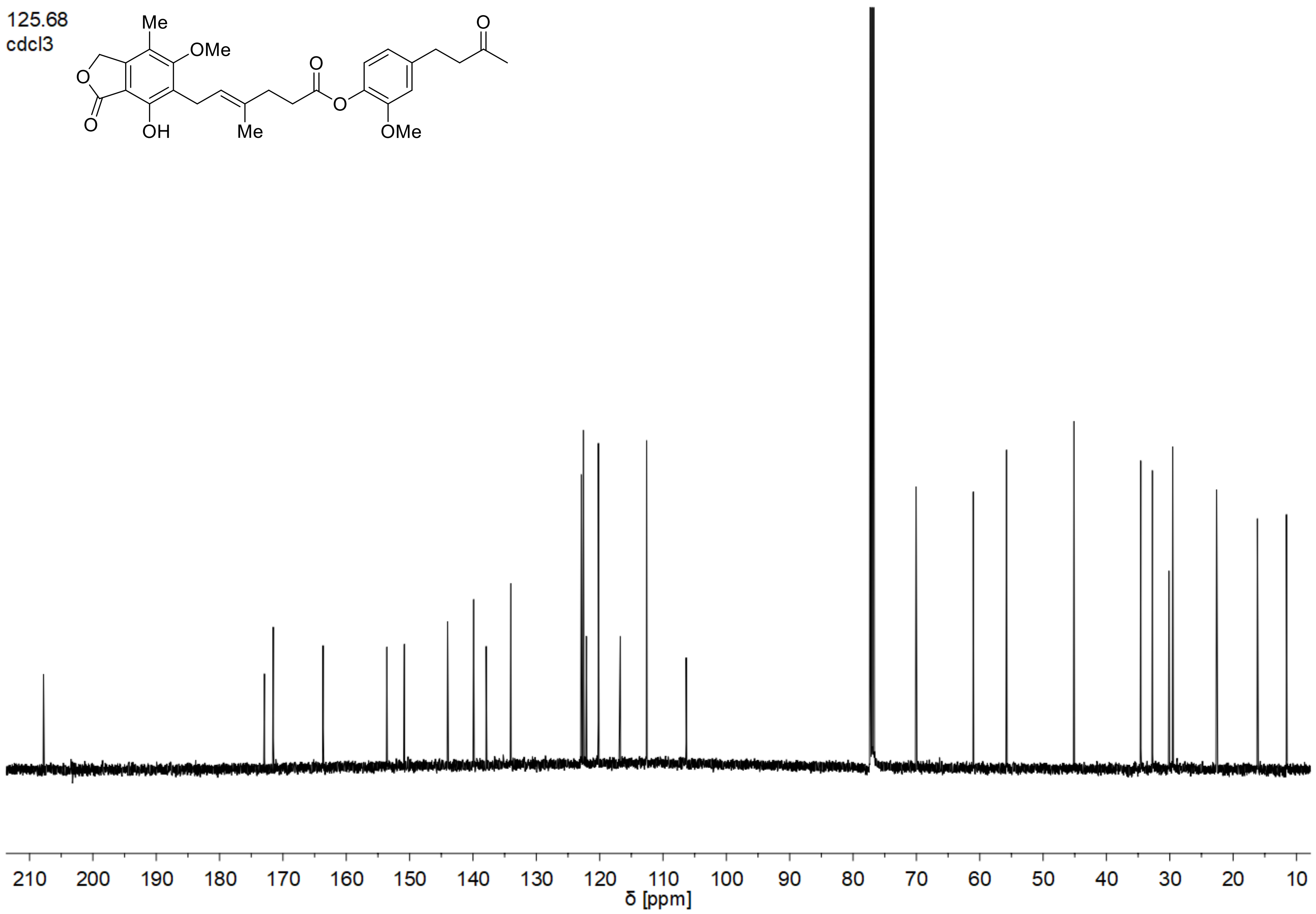

125.68
cdcl3
Me
OMe
O
O
OH
Me
O
O
O
OMe
210 200 190 180 170 160 150 140 130 120 110 100 90 80 70 60 50 40 30 20 10
δ [ppm]

NMR: (Methyl ferulate) mycophenolate (**MP7**):

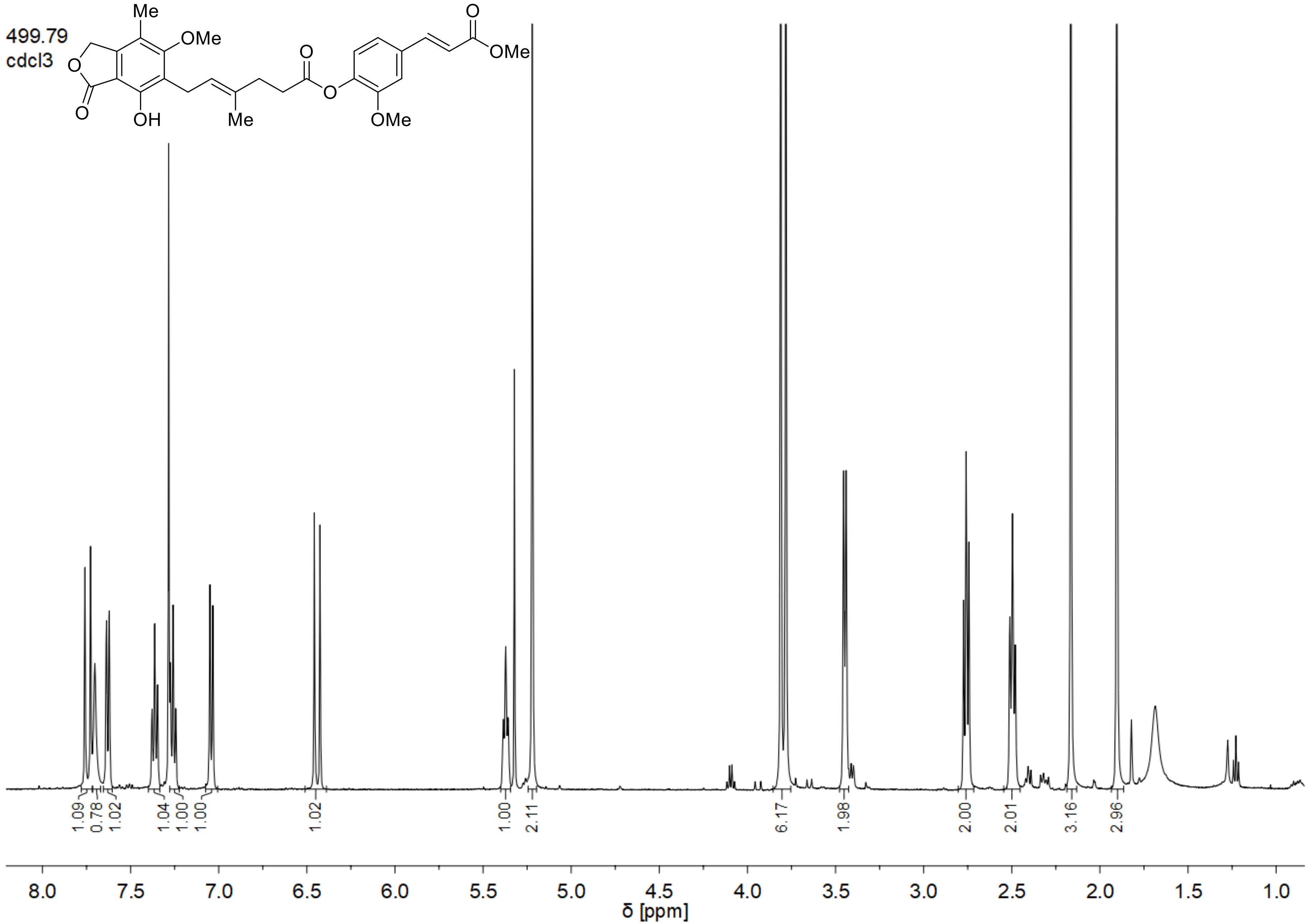

125.68
cdcl3

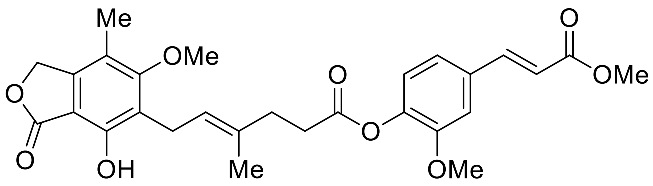
Me
OMe
O
O
O
OH
Me
O
O
OMe
O
OMe

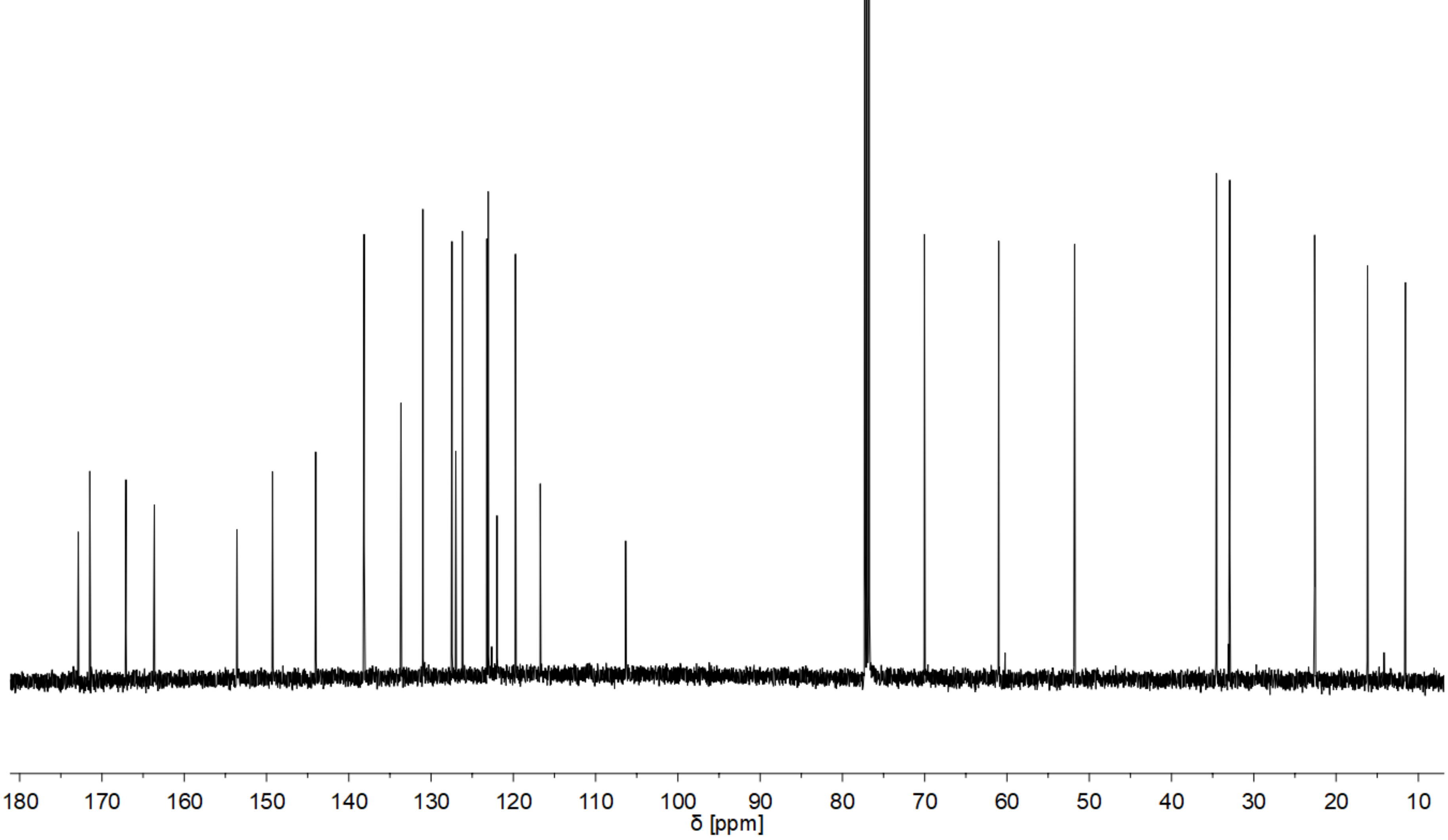
180
170
160
150
140
130
120
110
100
90
80
70
60
50
40
30
20
10
δ [ppm]

NMR: Eugenol mycophenolate (**MP8**):

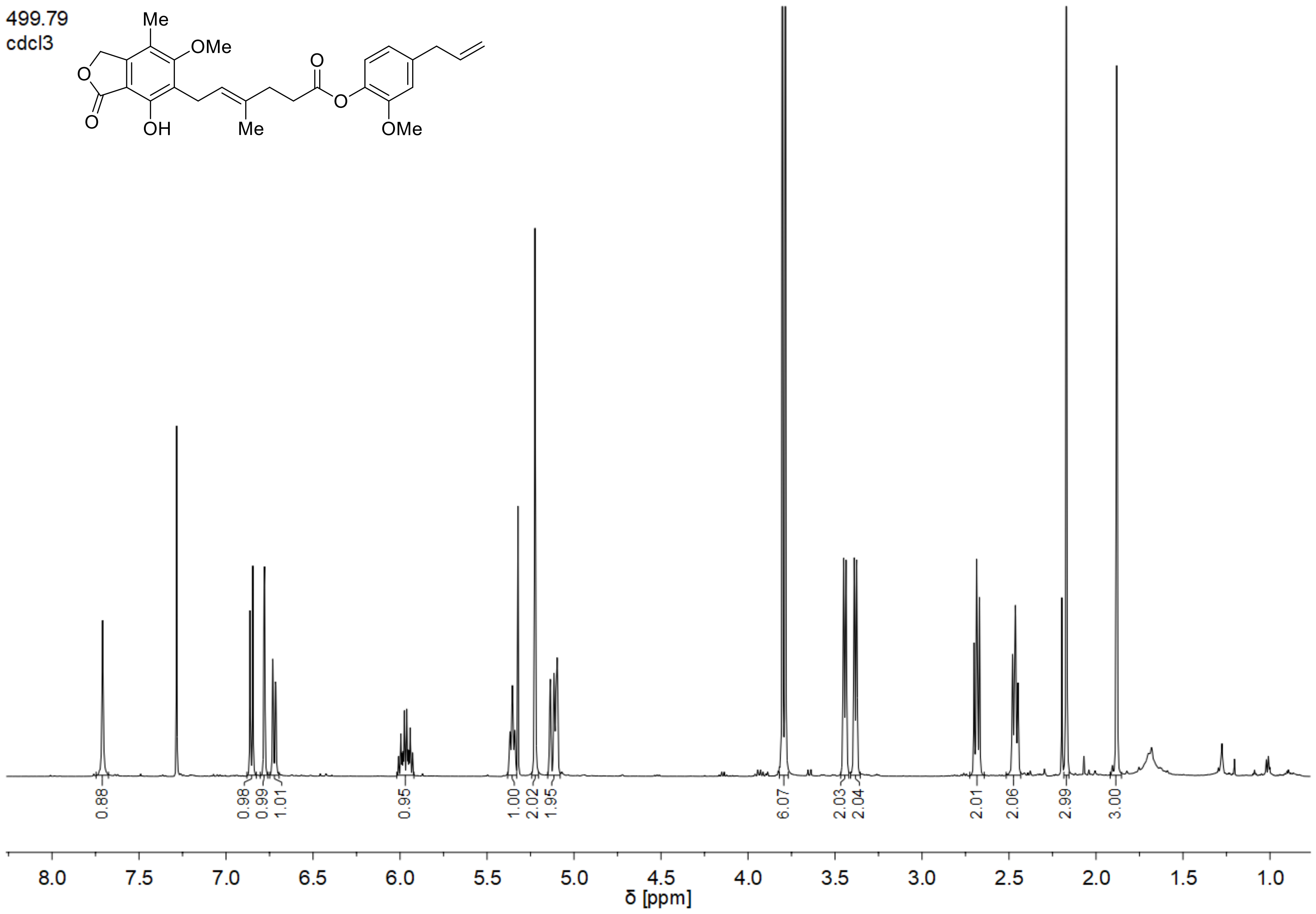

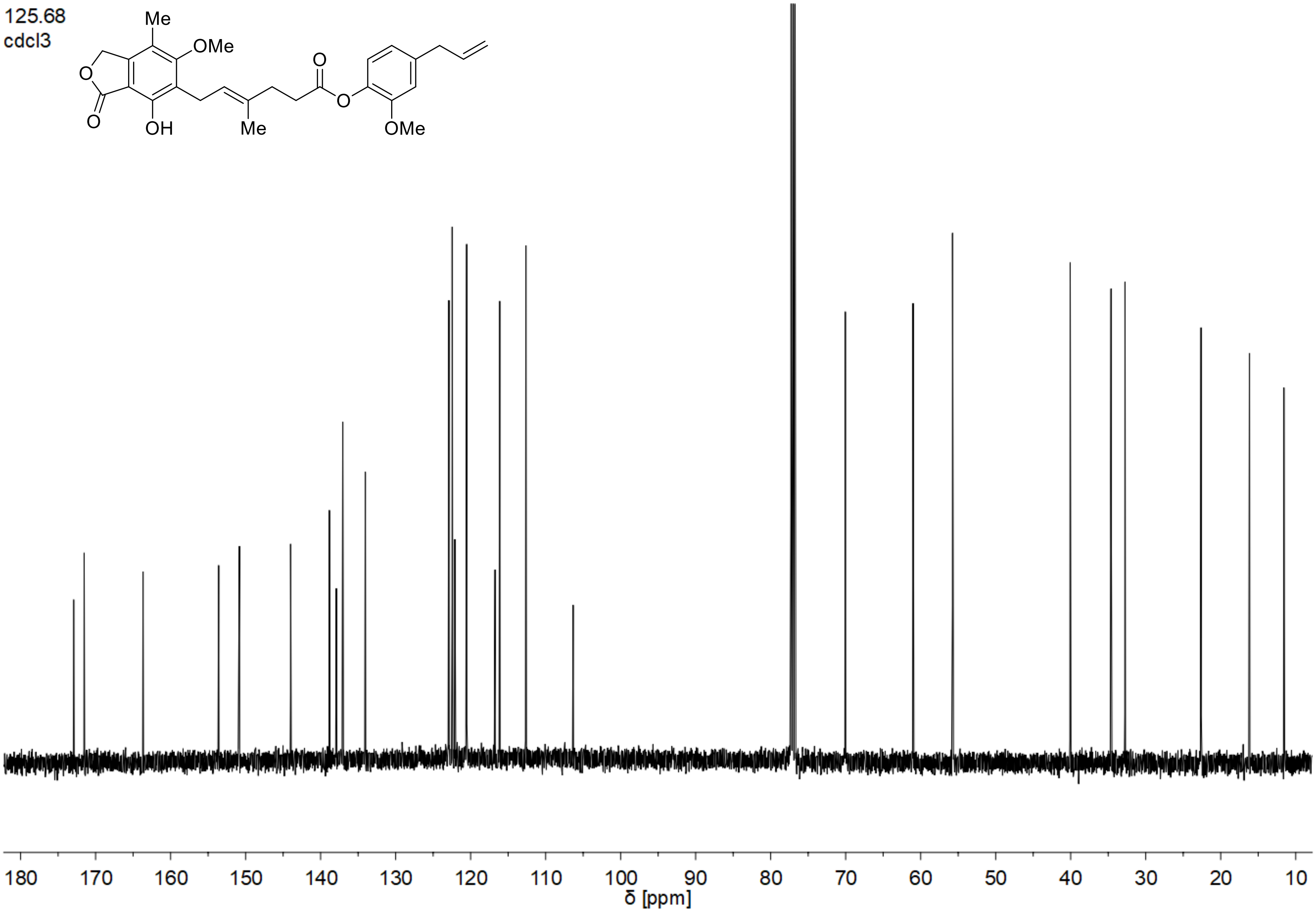
125.68
cdcl3
Me
OMe
O
O
O
OH
Me
O
OMe
180
170
160
150
140
130
120
110
100
90
80
70
60
50
40
30
20
10
δ [ppm]

NMR: Isoeugenol mycophenolate (**MP9**):

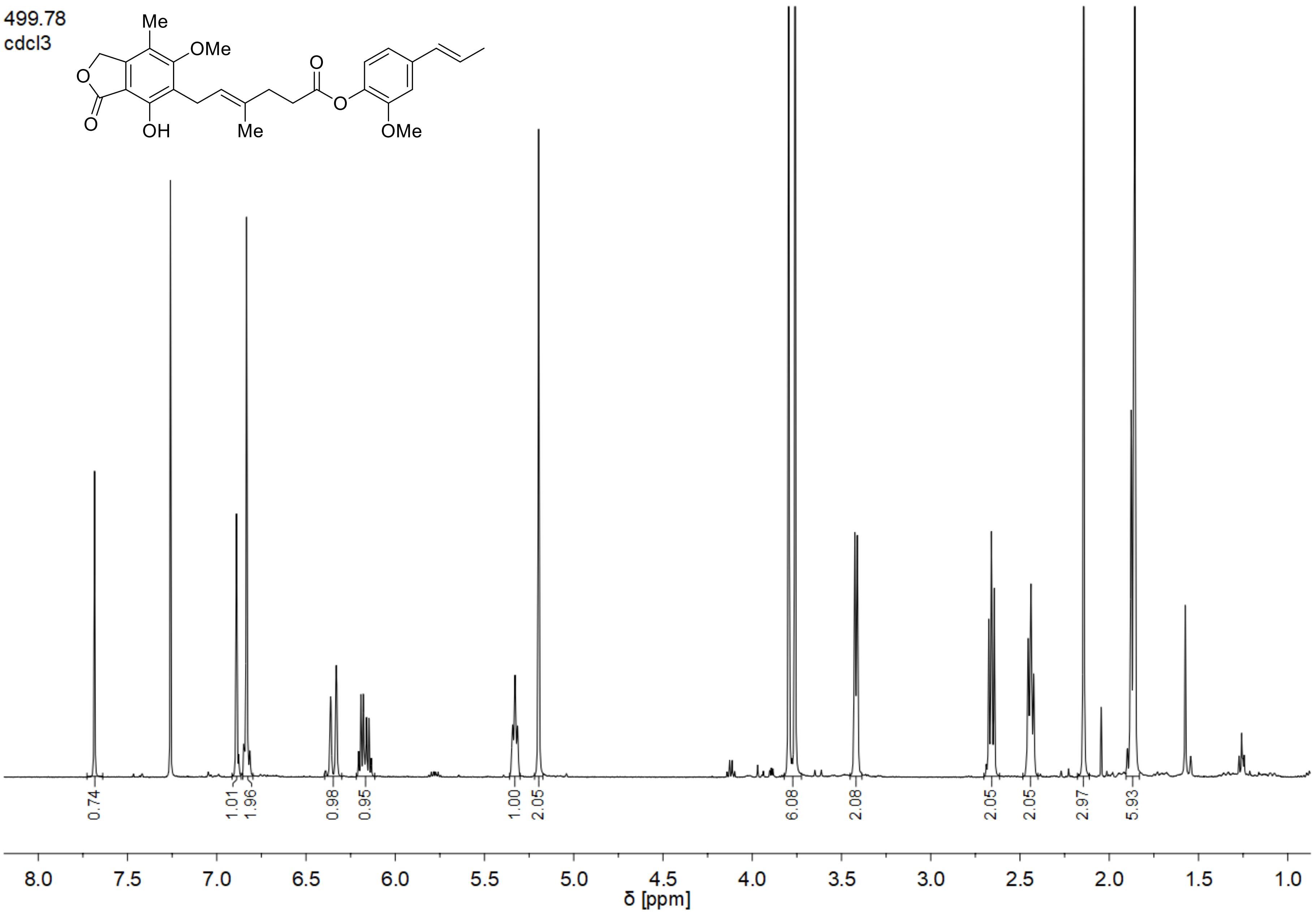

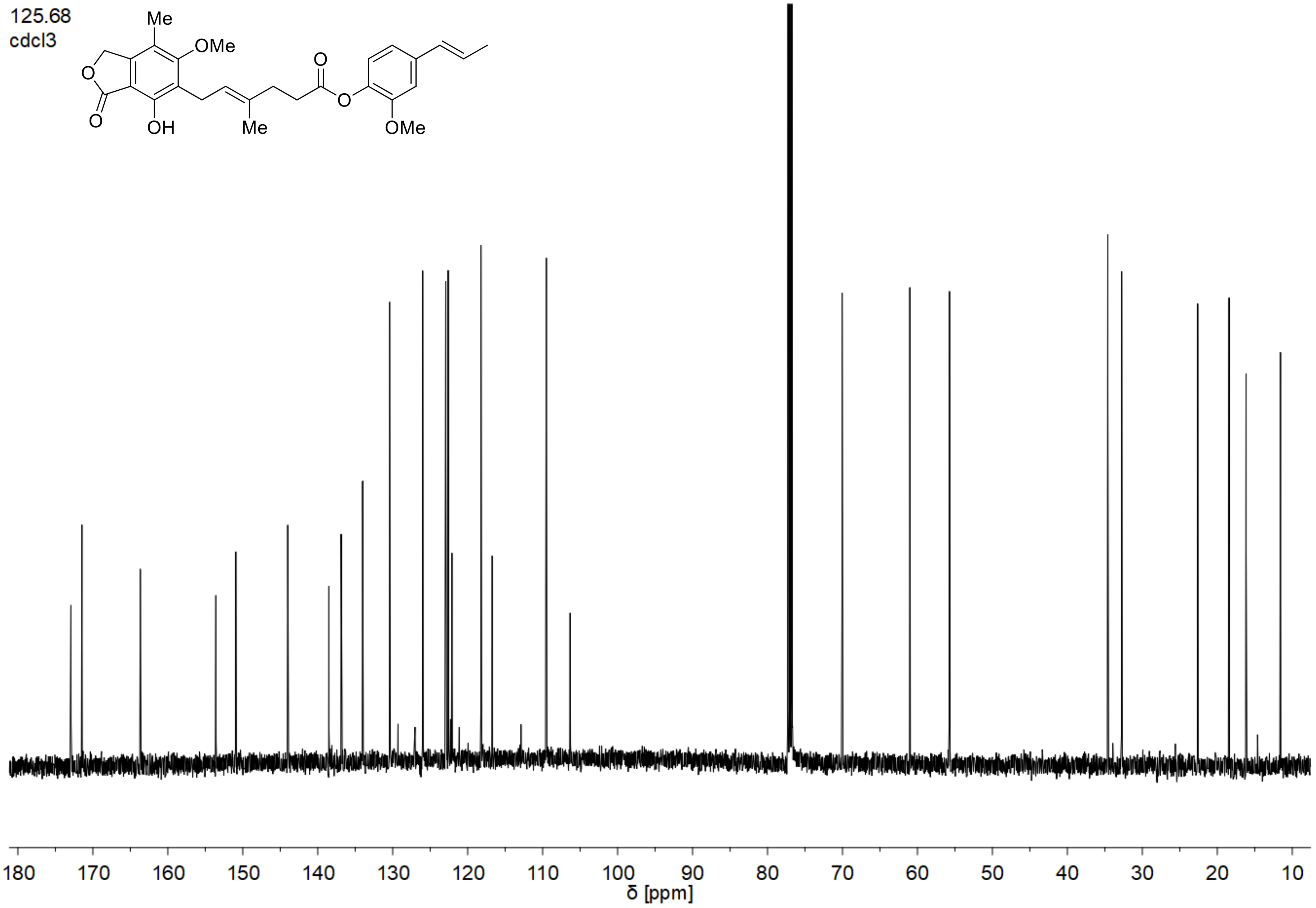
125.68
cdcl3
Me
OMe
O
O
OH
O
Me
O
O
OMe
180 170 160 150 140 130 120 110 100 90 80 70 60 50 40 30 20 10
δ [ppm]

NMR: Sesamol mycophenolate (**P1**):

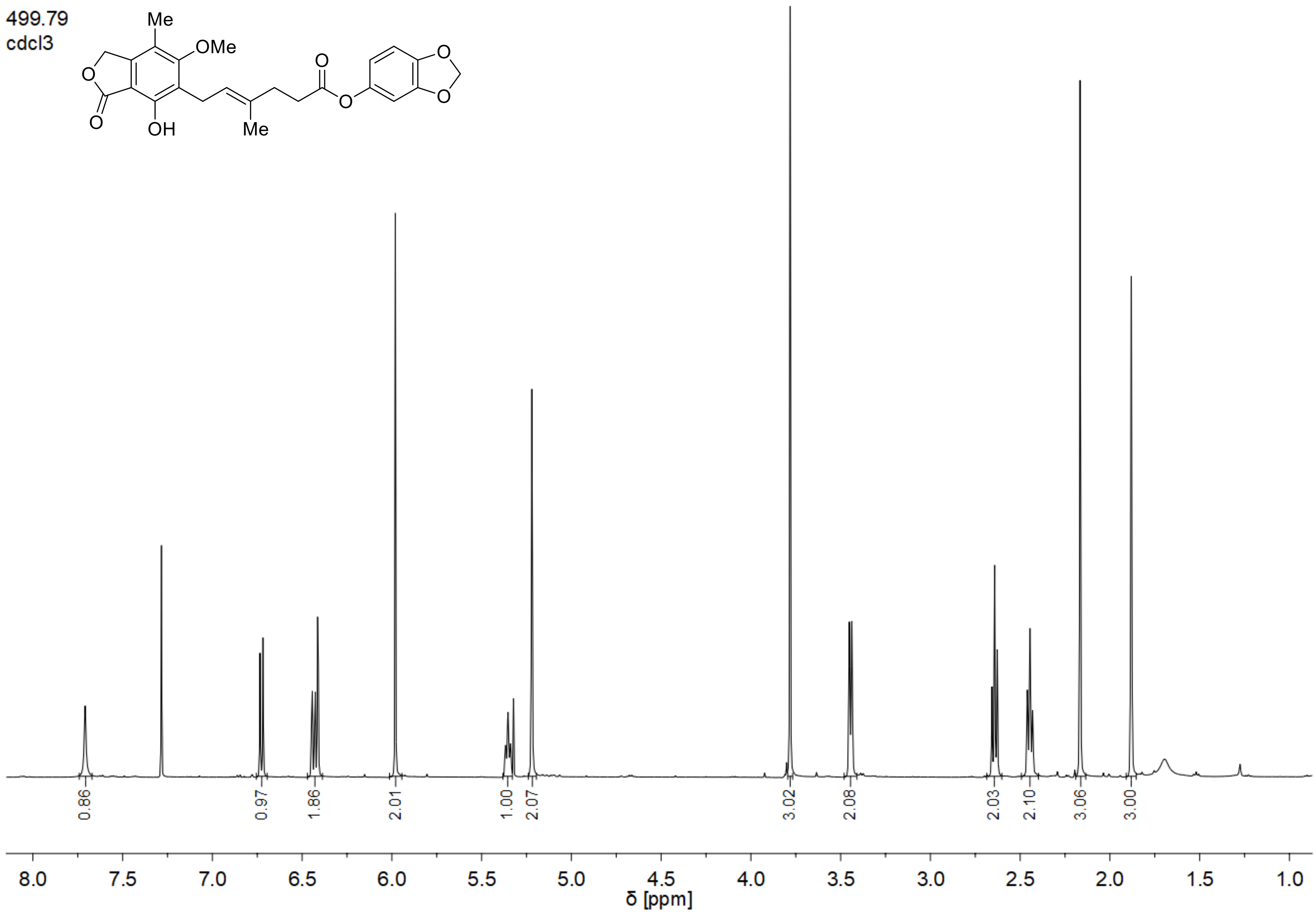

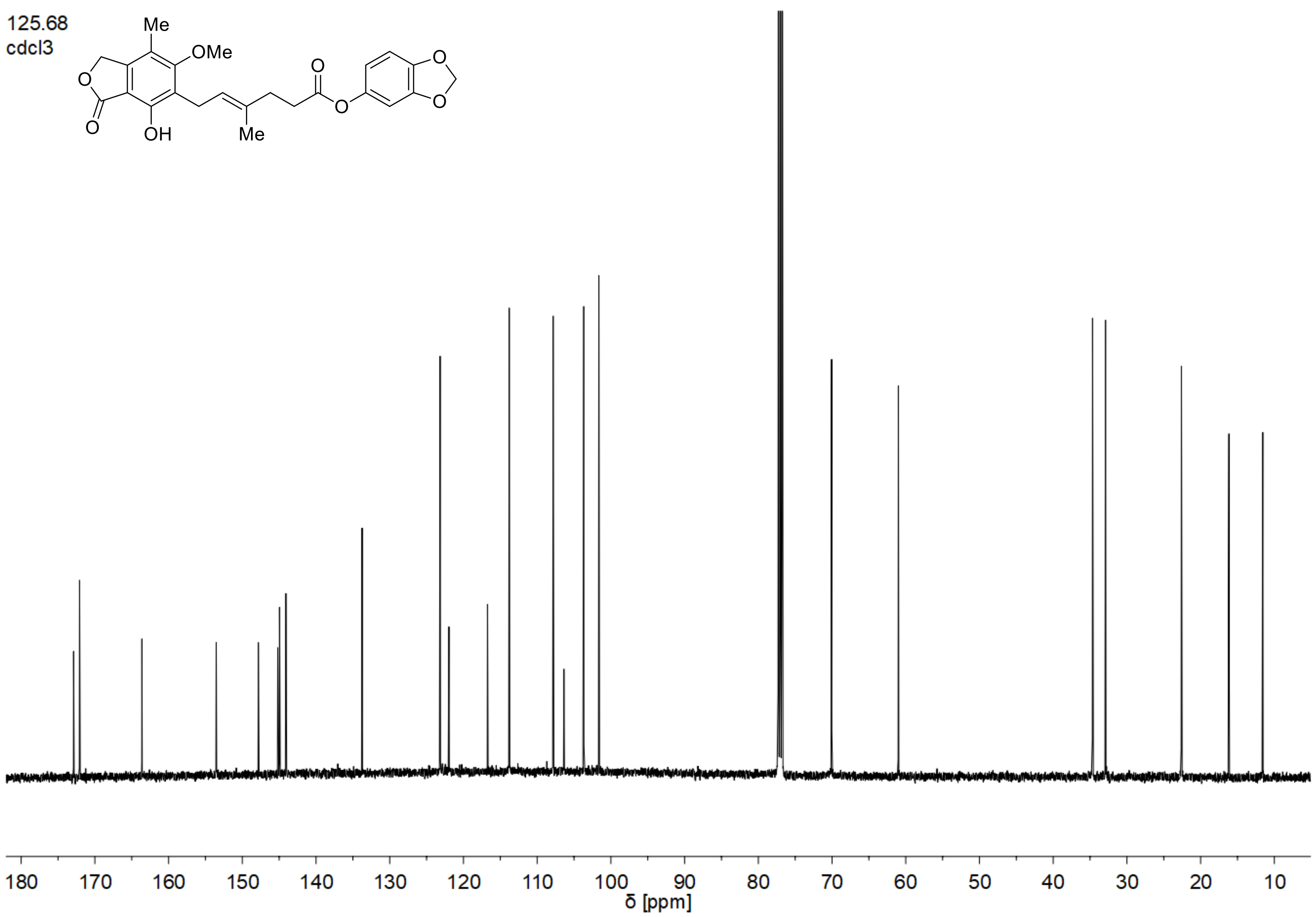

125.68
cdcl3
Me
OMe
O
O
O
O
O
O
OH
Me
δ [ppm]

NMR: Thymol mycophenolate (**P2**):

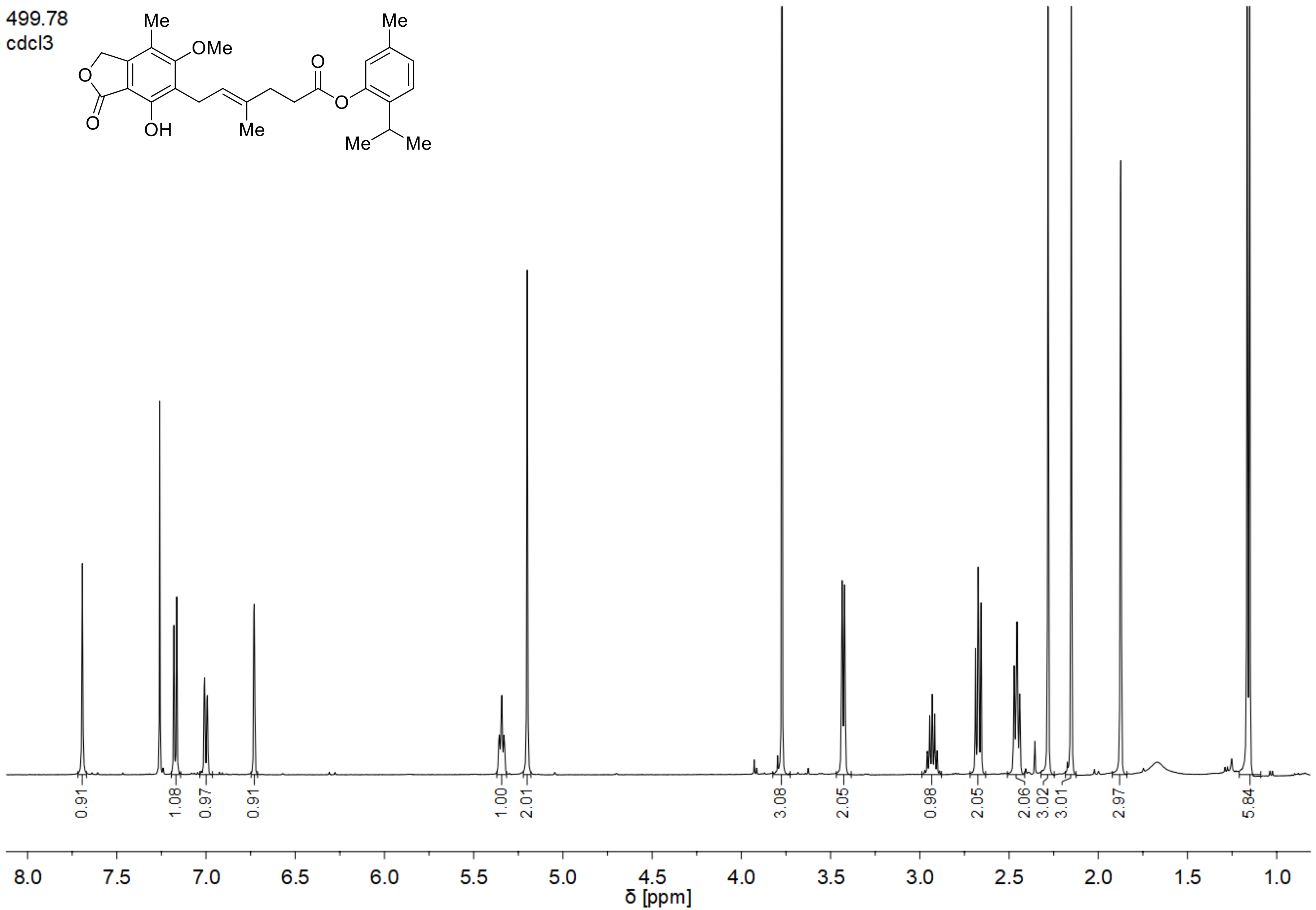

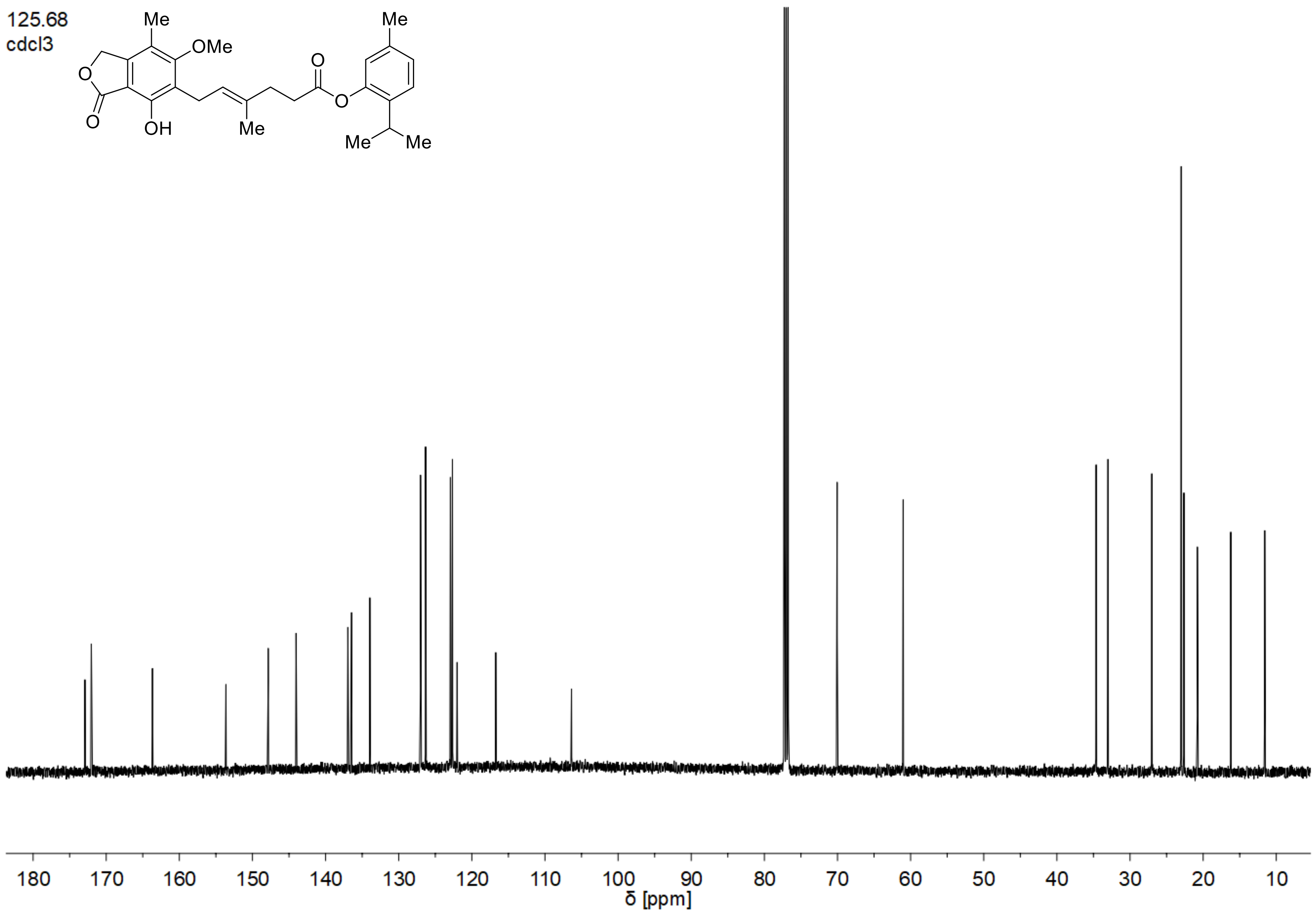
125.68
cdcl3
Me
OMe
O
O
O
OH
Me
O
O
Me
Me
Me
180
170
160
150
140
130
120
110
100
90
80
70
60
50
40
30
20
10
δ [ppm]

NMR: Bakuchiol mycophenolate (**P3**):

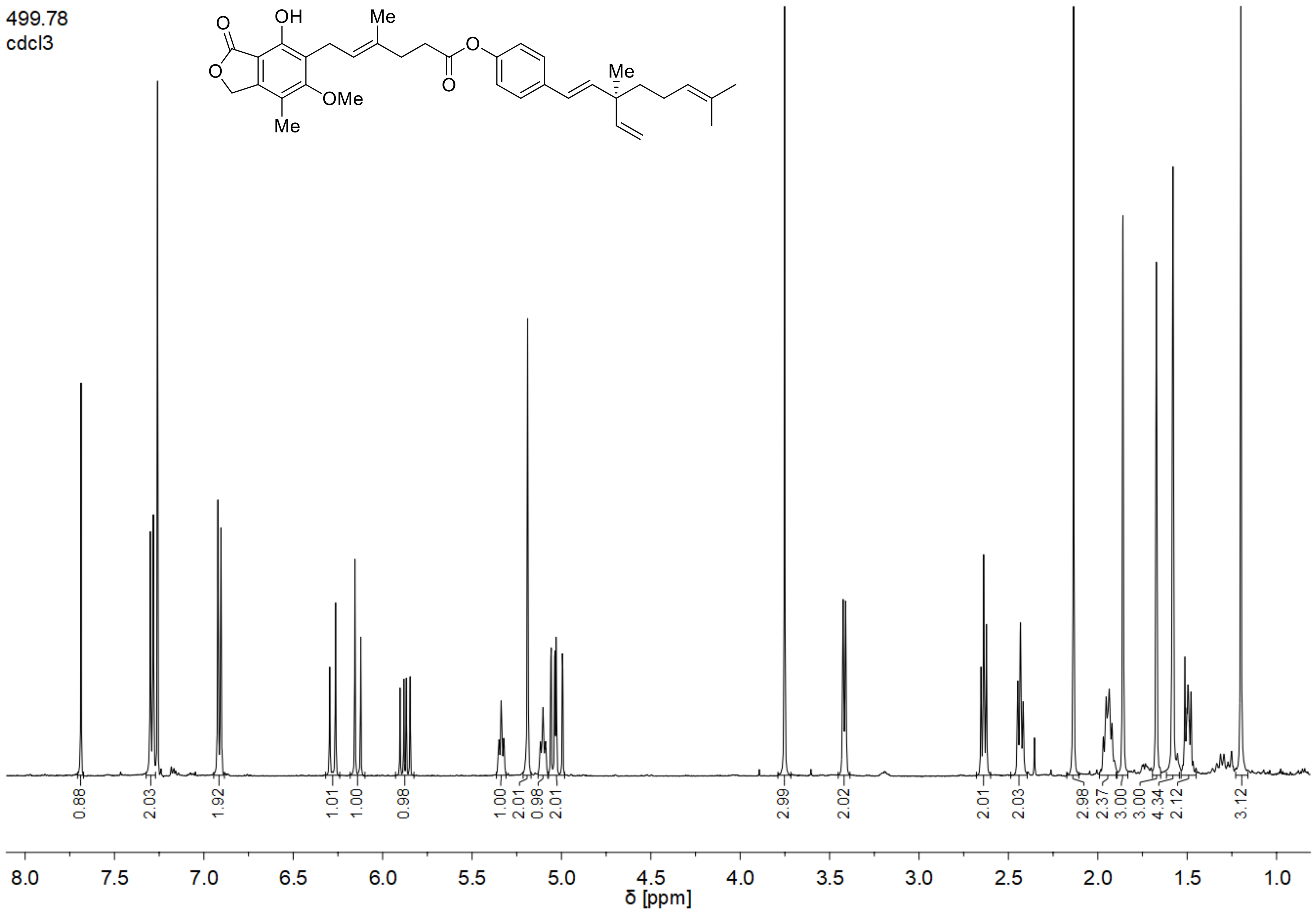

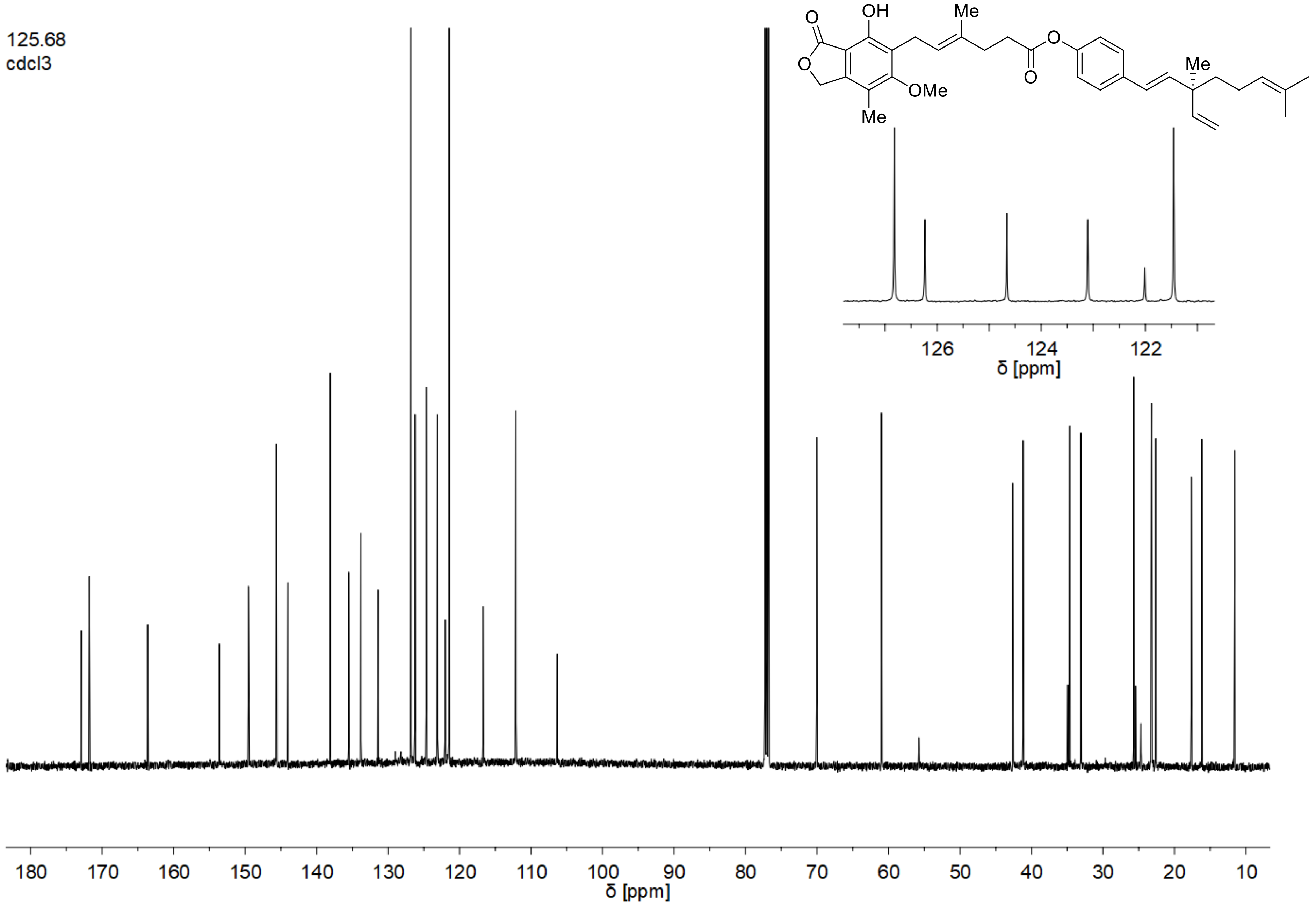
125.68
cdcl3
OH
Me
O
O
OMe
Me
O
O
Me
126
124
122
δ [ppm]
180
170
160
150
140
130
120
110
100
90
80
70
60
50
40
30
20
10
δ [ppm]

NMR: (Methyl *o*-coumarate) mycophenolate (**P4**):

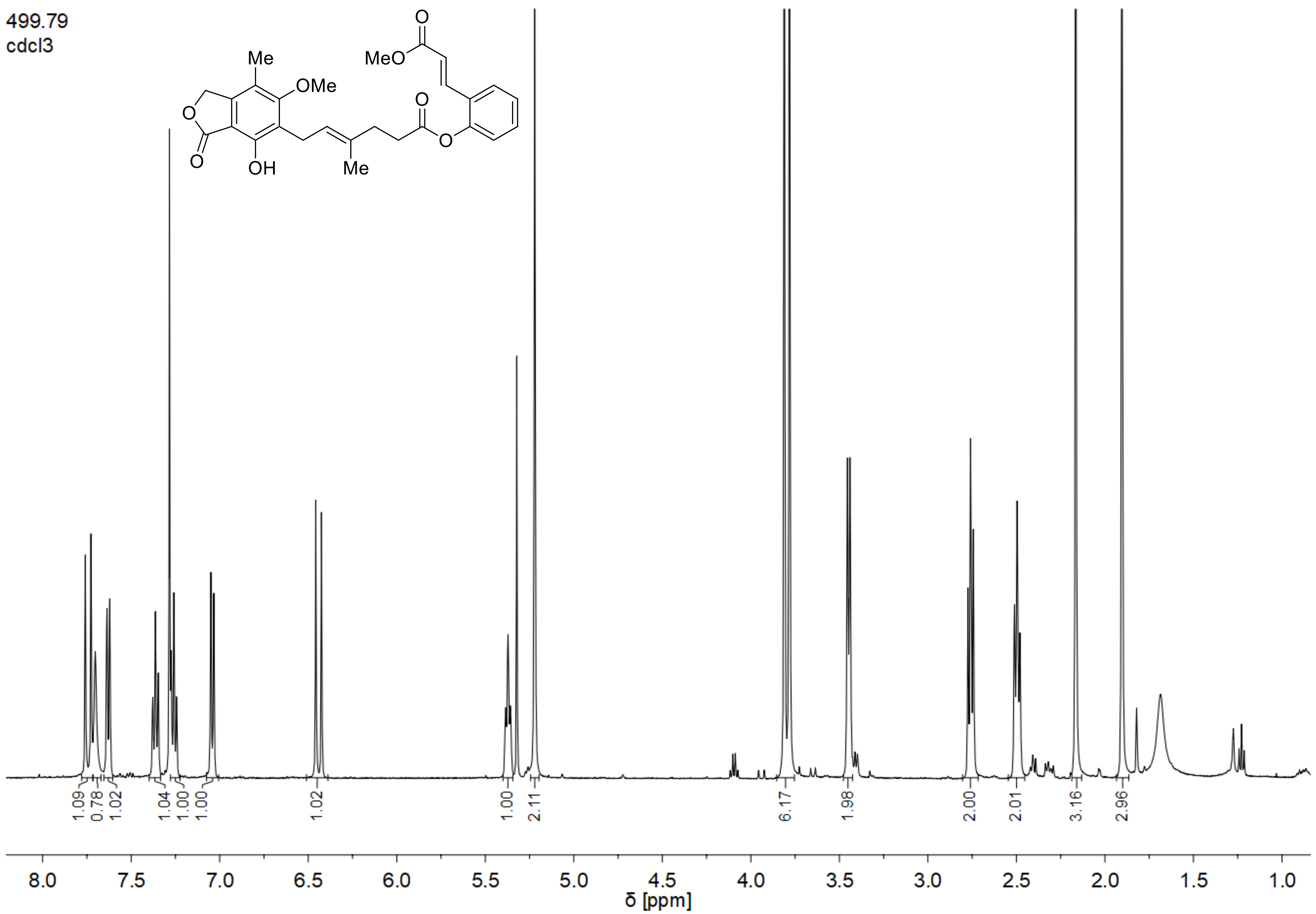

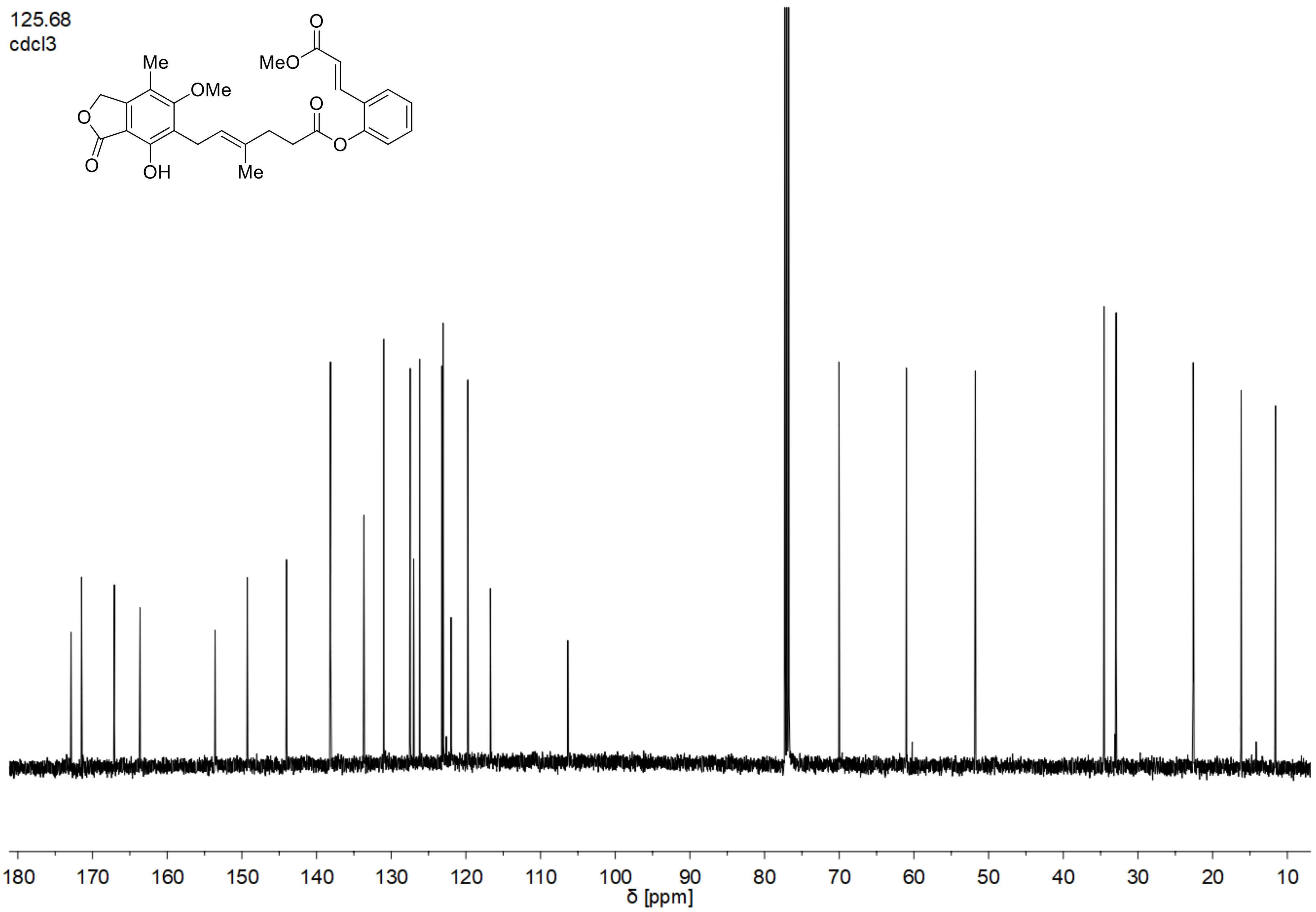
125.68
cdcl3
Me
OMe
O
O
OH
Me
MeO
O
O
O
180 170 160 150 140 130 120 110 100 90 80 70 60 50 40 30 20 10
δ [ppm]

NMR: 4-Hydroxybenzaldehyde mycophenolate (**P5**):

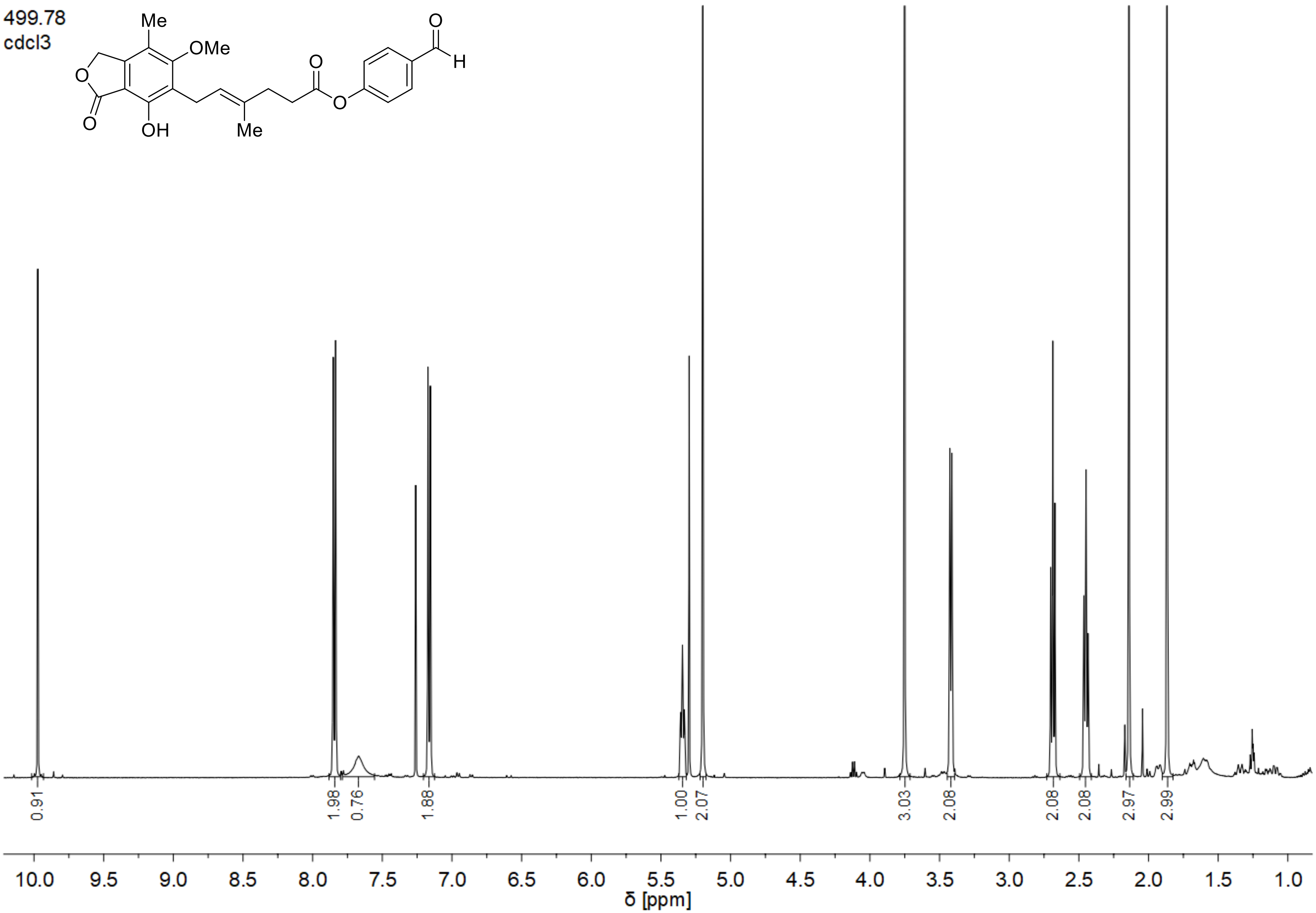

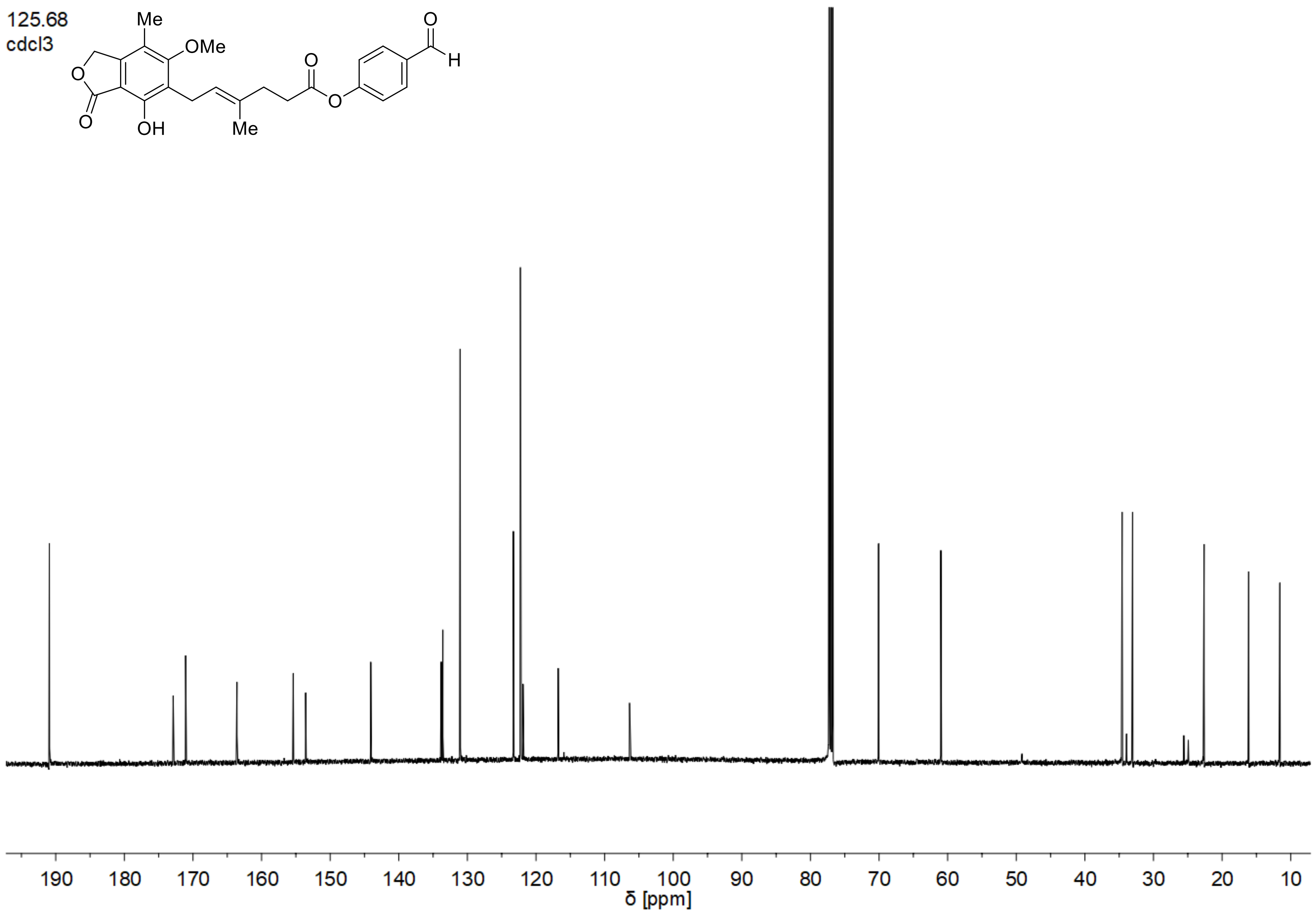
125.68
cdcl3
Me
OMe
O
O
H
O
O
O
OH
Me
190
180
170
160
150
140
130
120
110
100
90
80
70
60
50
40
30
20
10
δ [ppm]

NMR: Dehydroframbinone mycophenolate (**P6**):

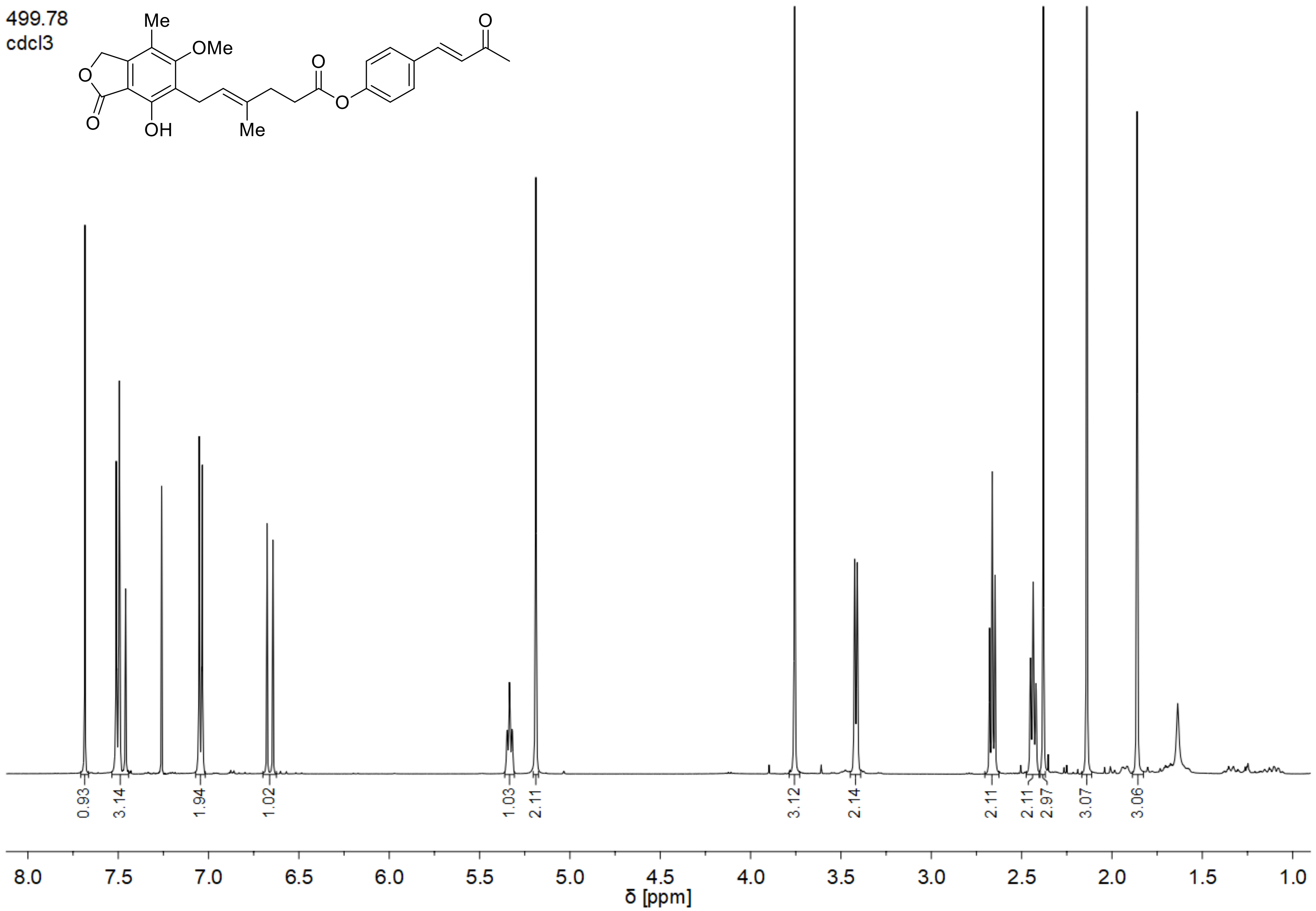

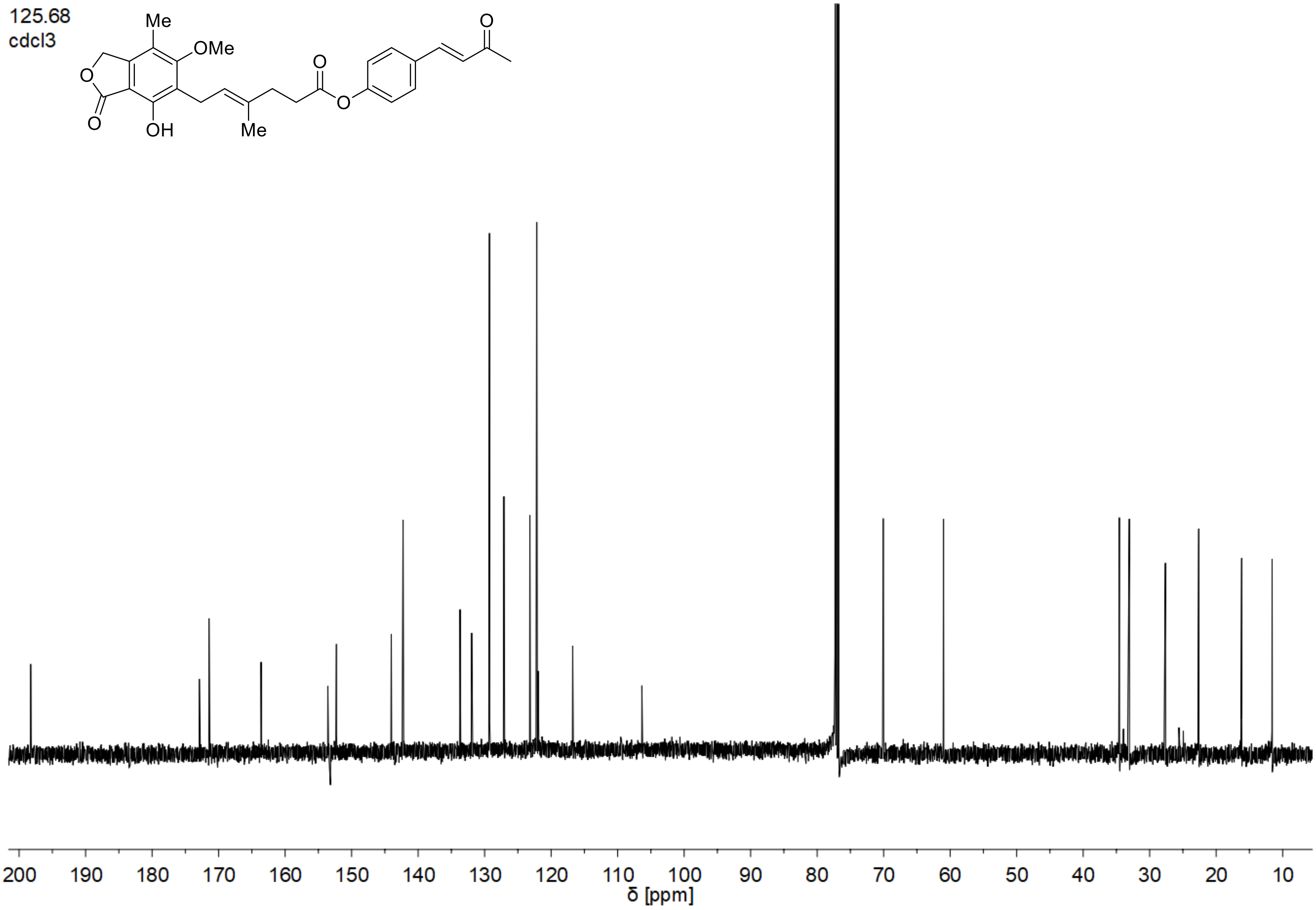
125.68
cdcl3
Me
OMe
O
O
OH
Me
O
O
O
O
200 190 180 170 160 150 140 130 120 110 100 90 80 70 60 50 40 30 20 10
δ [ppm]

NMR: Raspberry ketone mycophenolate (**P7**):

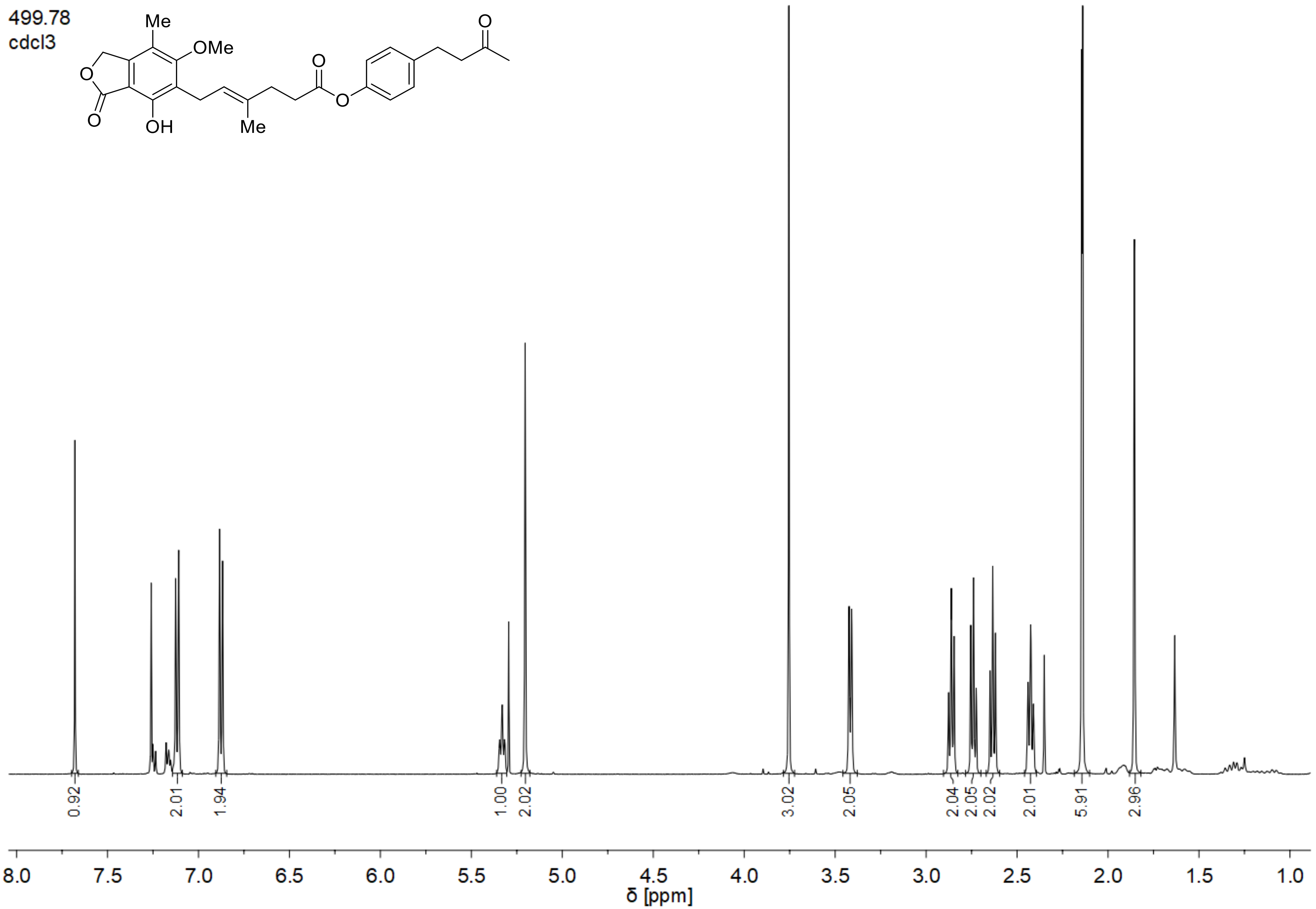

125.68
cdcl3

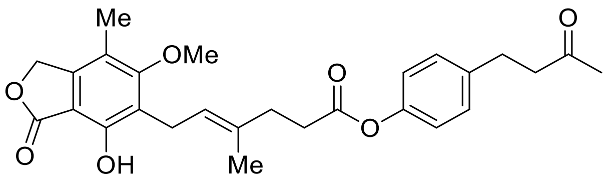

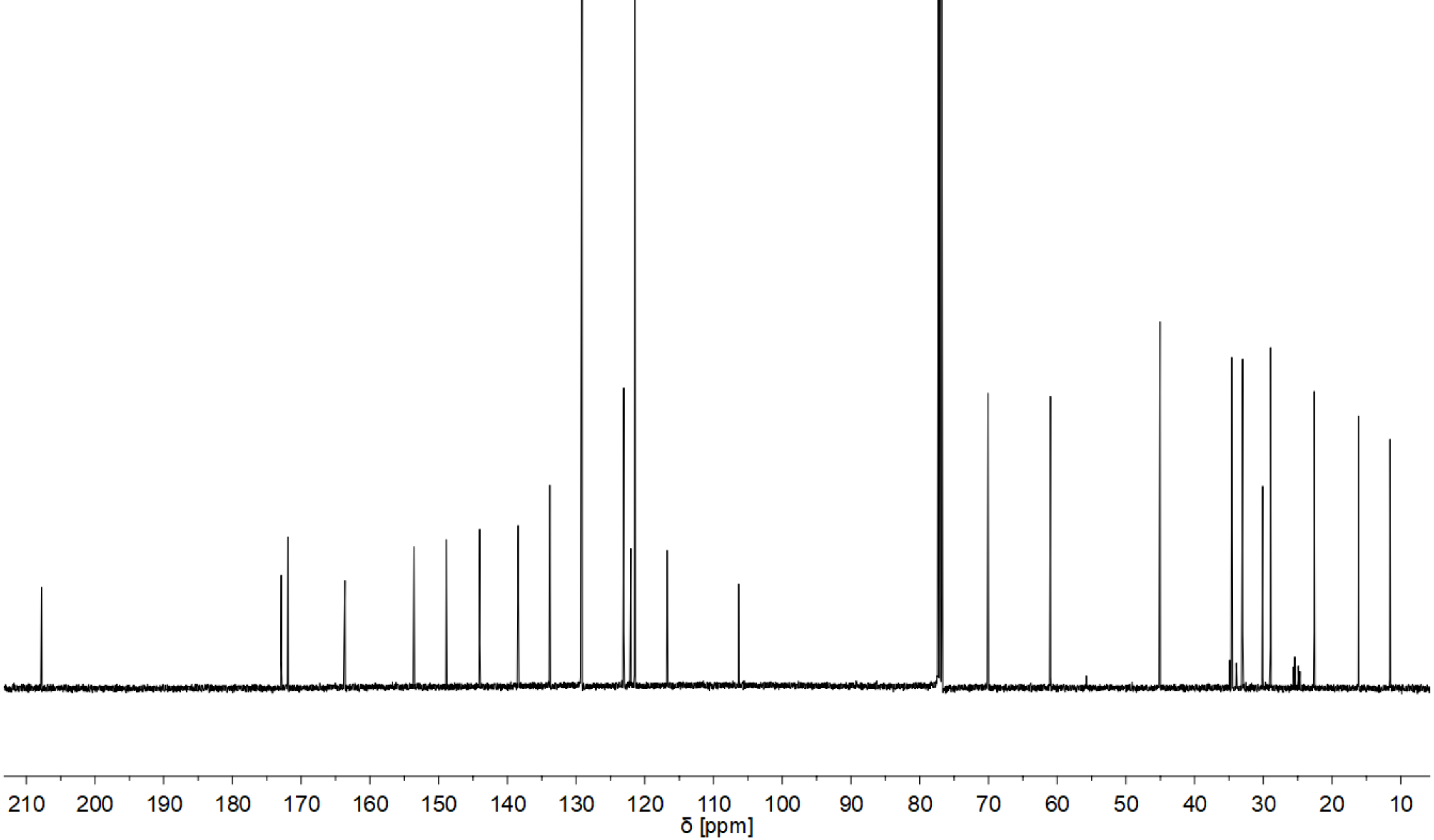
210
200
190
180
170
160
150
140
130
120
110
100
90
80
70
60
50
40
30
20
10
δ [ppm]

HPLC: Mycophenolic acid (**MPA**):

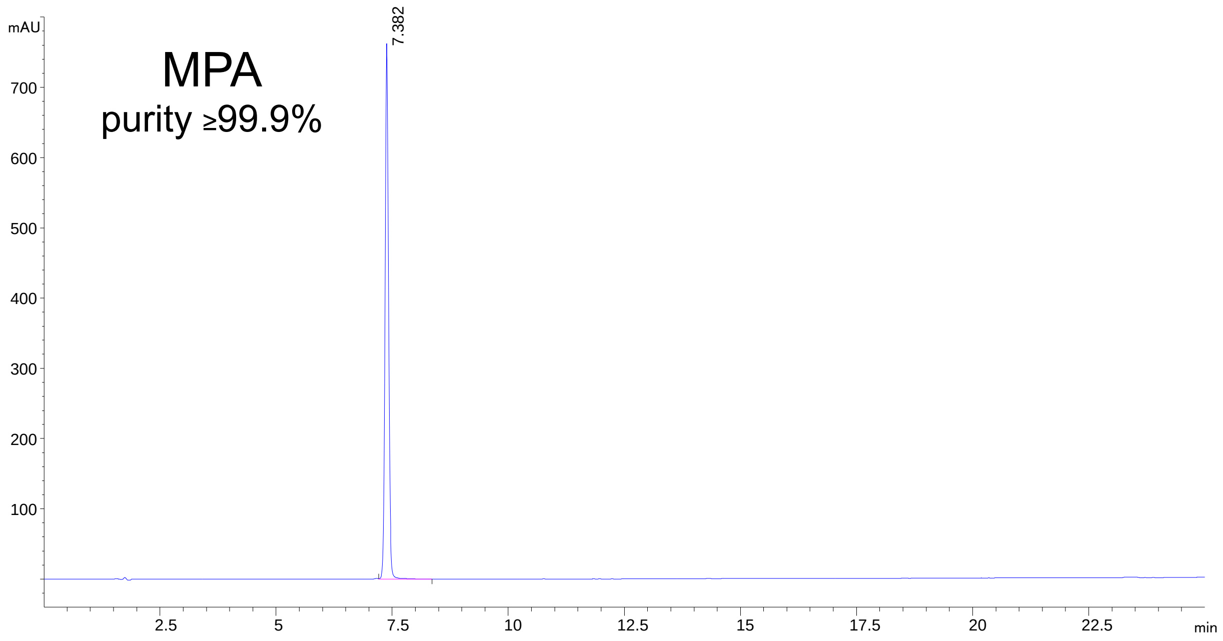

HPLC: Mycophenolate mofetil (**MMF**):

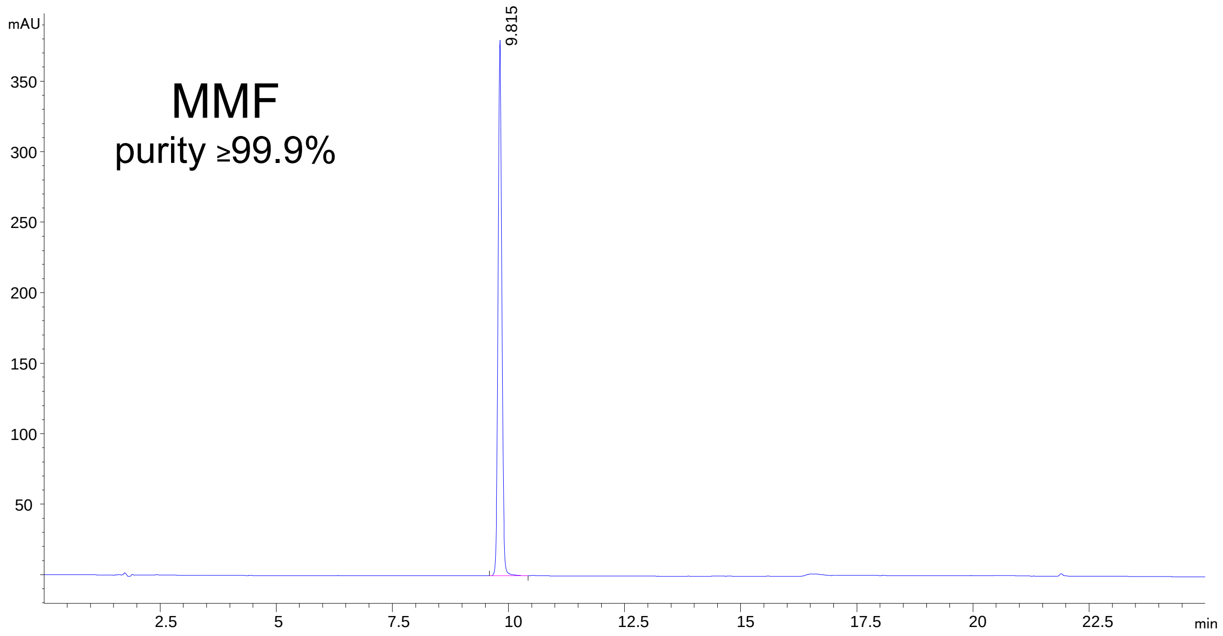

HPLC: Guaiacol mycophenolate (**MP1**):

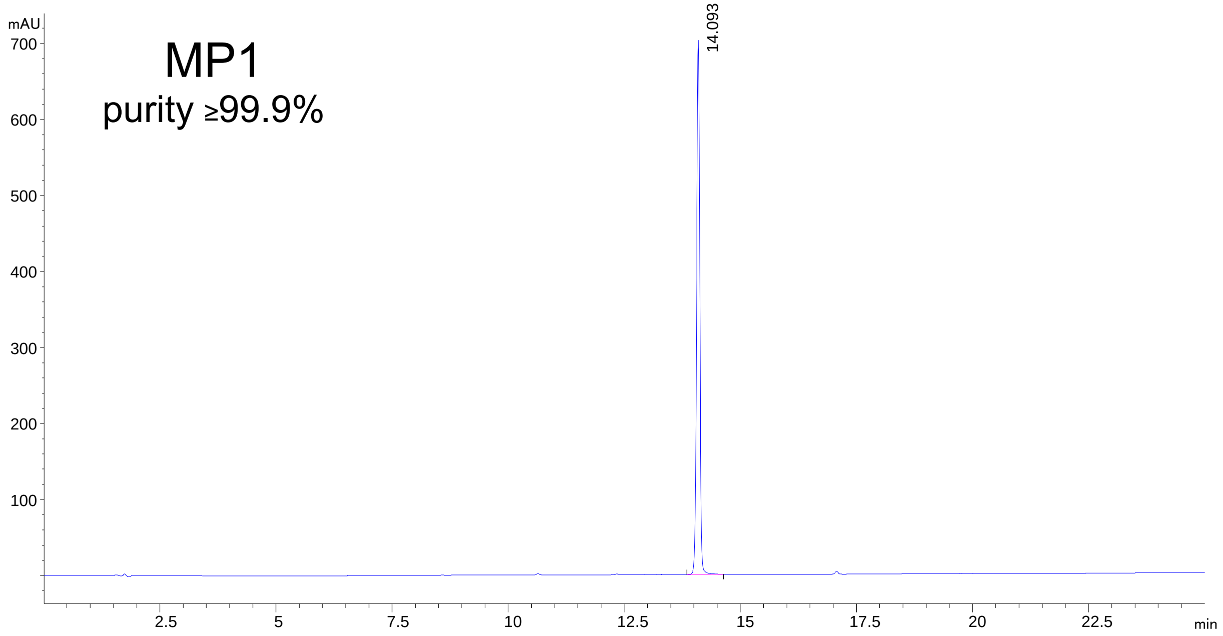

HPLC: Syringol mycophenolate (**MP2**):

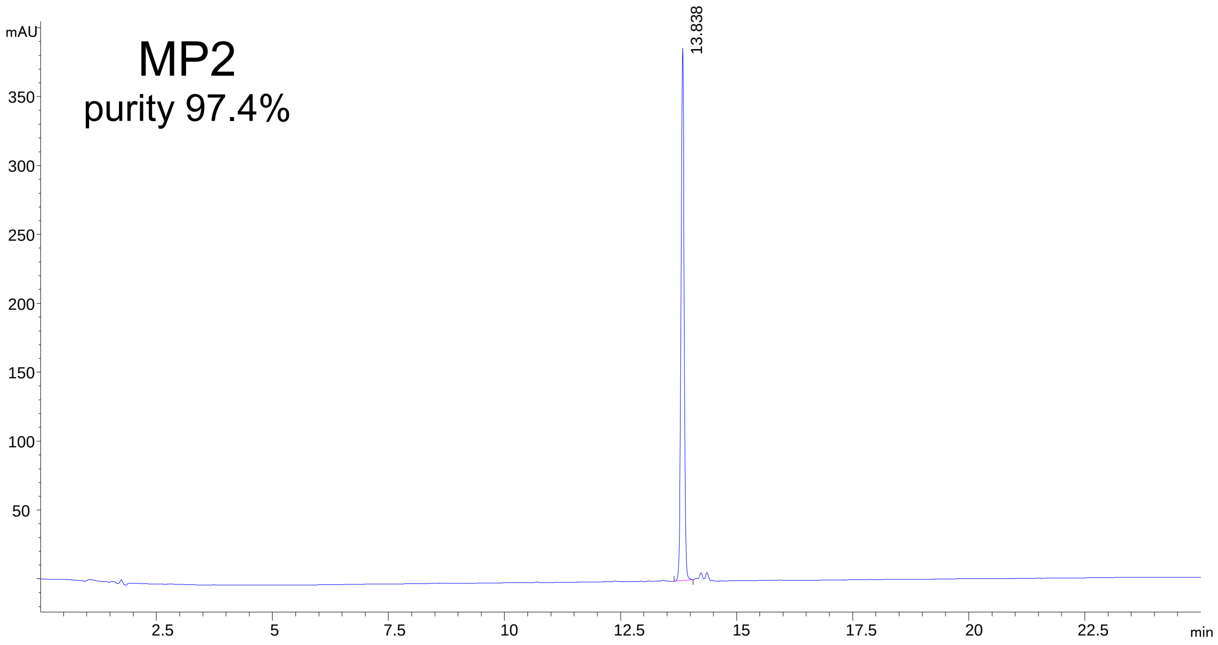

HPLC: Syringaldehyde mycophenolate (**MP3**):

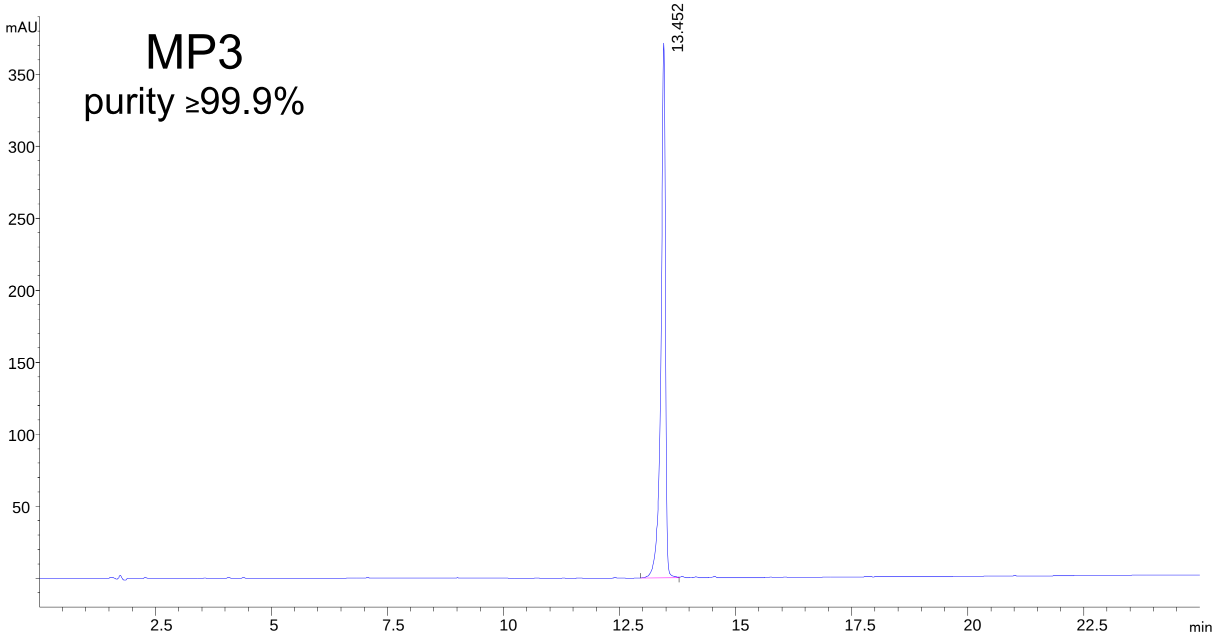

HPLC: Vanillin mycophenolate (**MP4**):

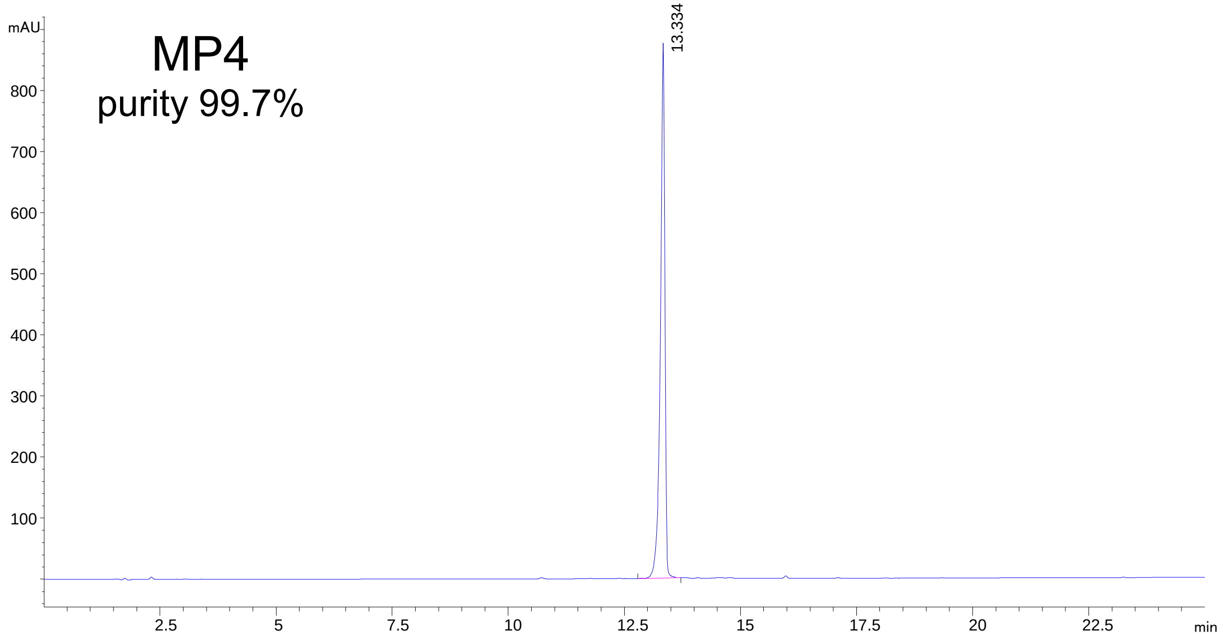

HPLC: Dehydrozingerone mycophenolate (**MP5**):

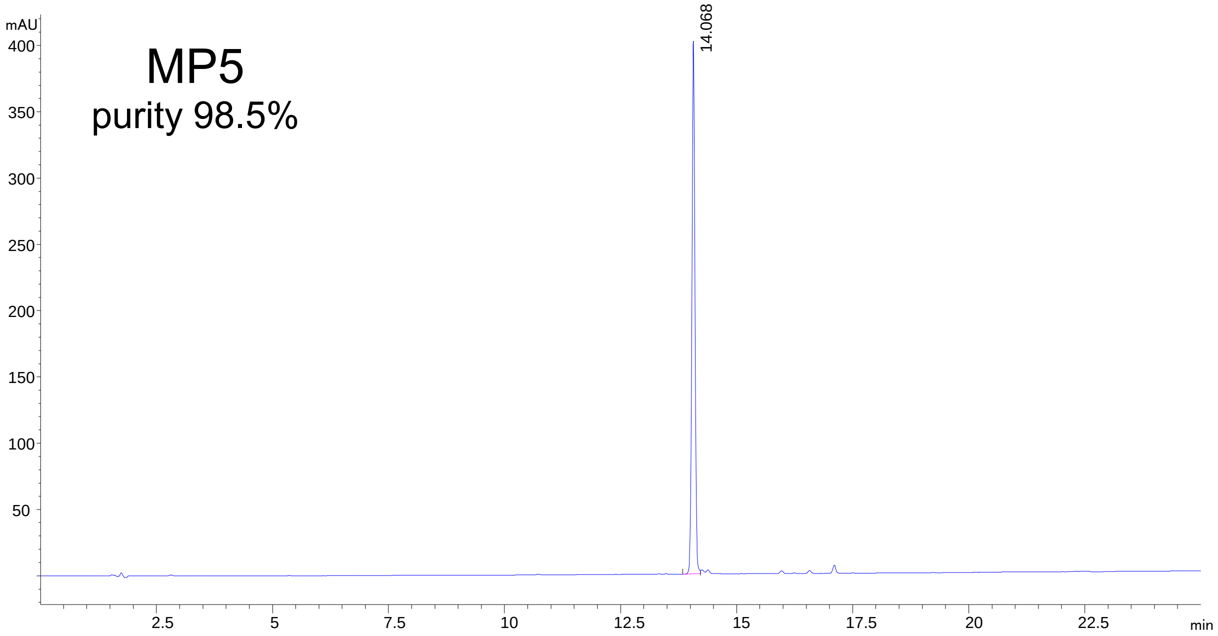

HPLC: Zingerone mycophenolate (**MP6**):

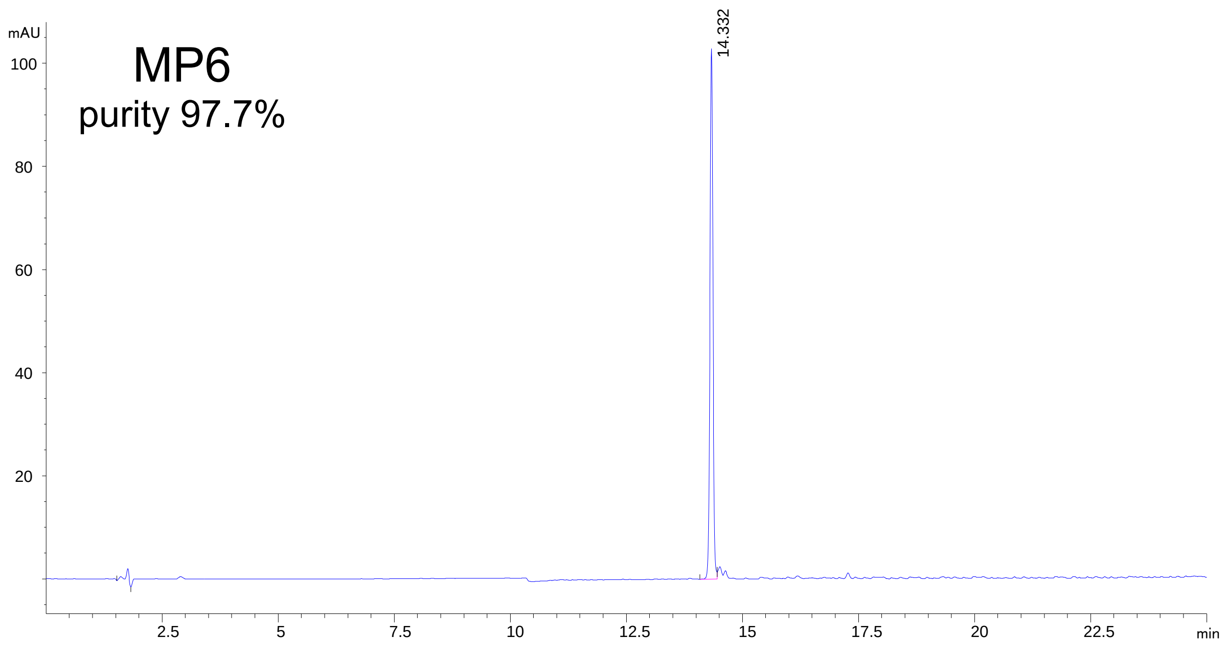

HPLC: (Methyl ferulate) mycophenolate (**MP7**):

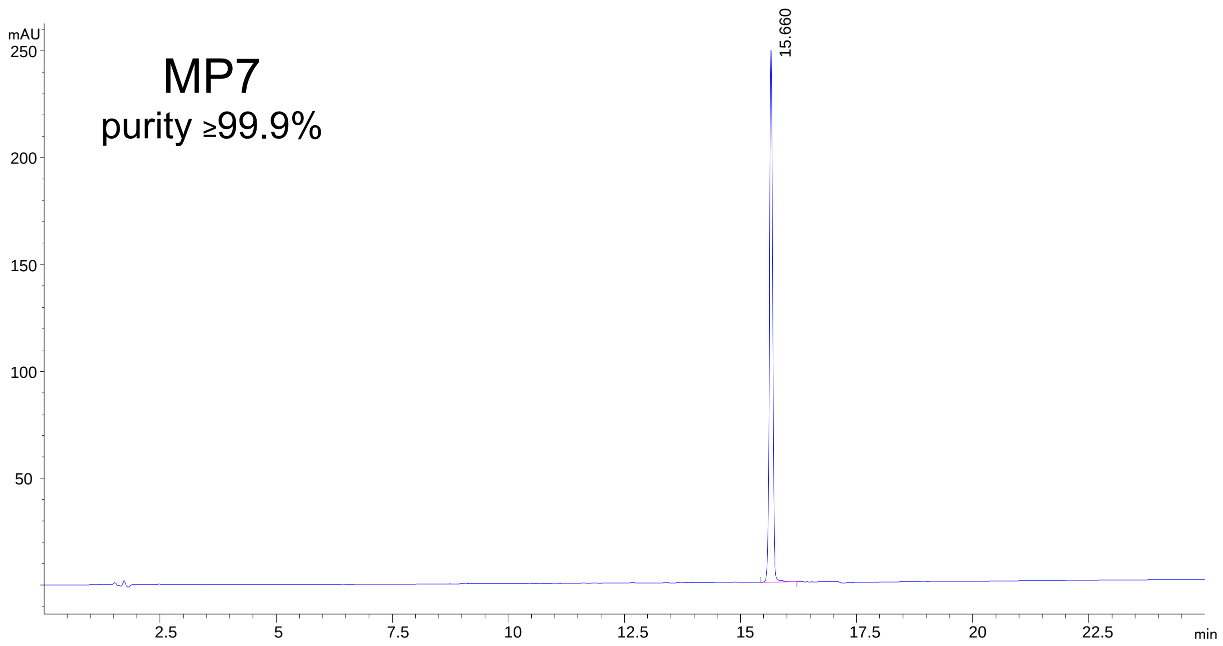

HPLC:Eugenol mycophenolate (**MP8**):

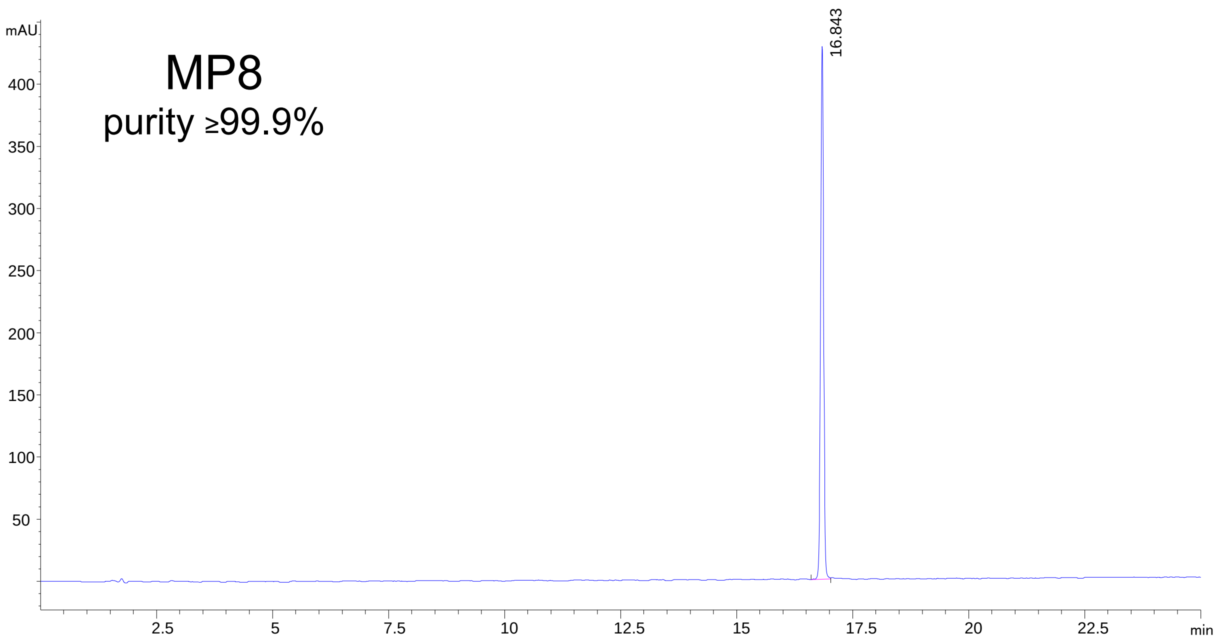

HPLC: Isoeugenol mycophenolate (**MP9**):

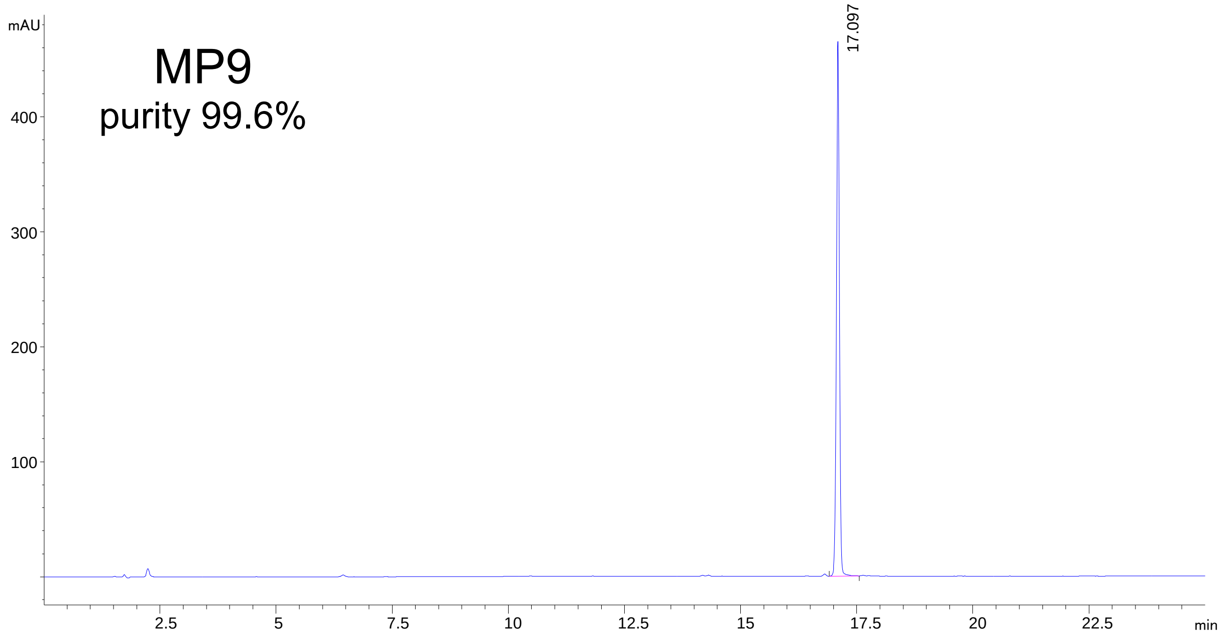

HPLC: Sesamol mycophenolate (**P1**):

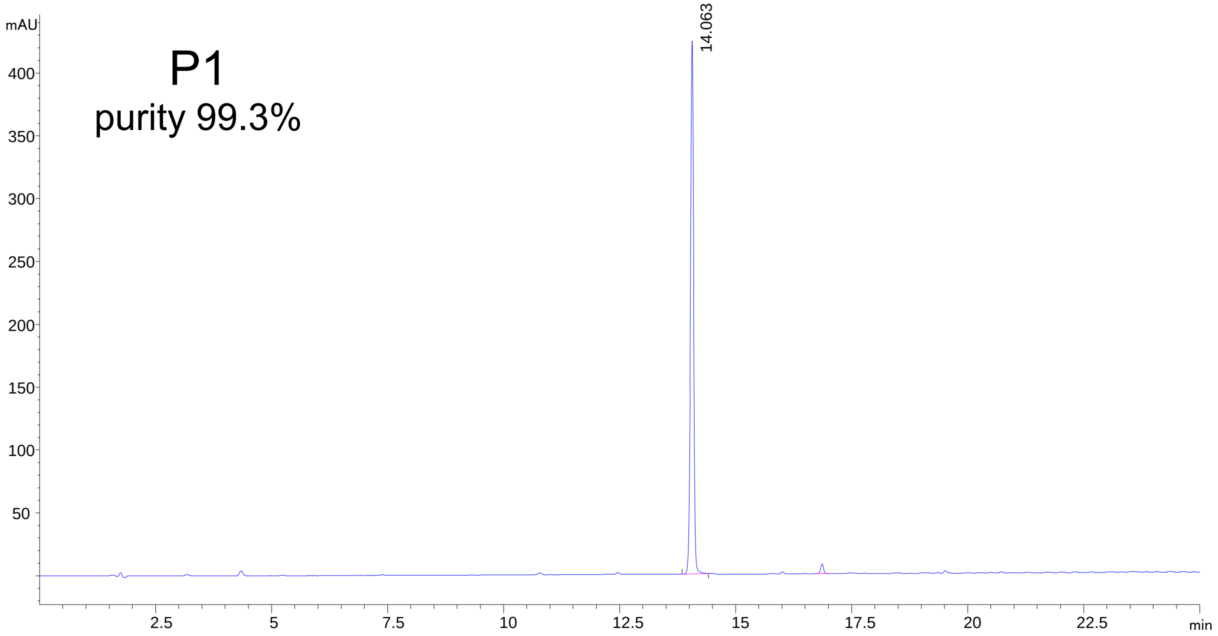

HPLC: Thymol mycophenolate (**P2**):

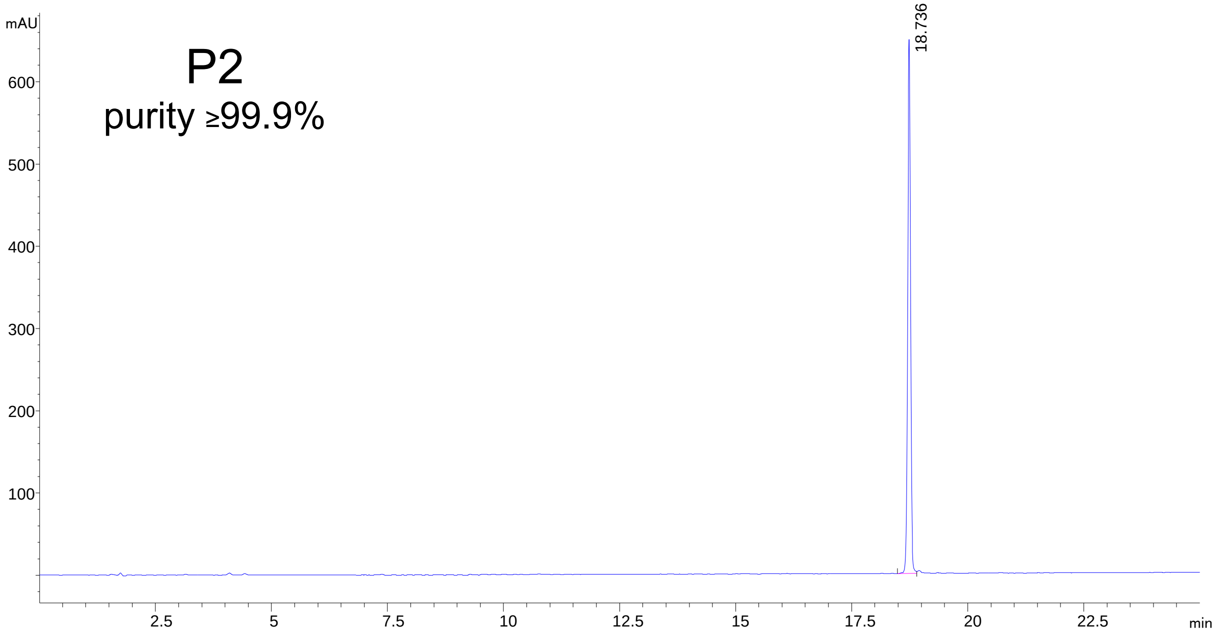

HPLC: Bakuchiol mycophenolate (**P3**):

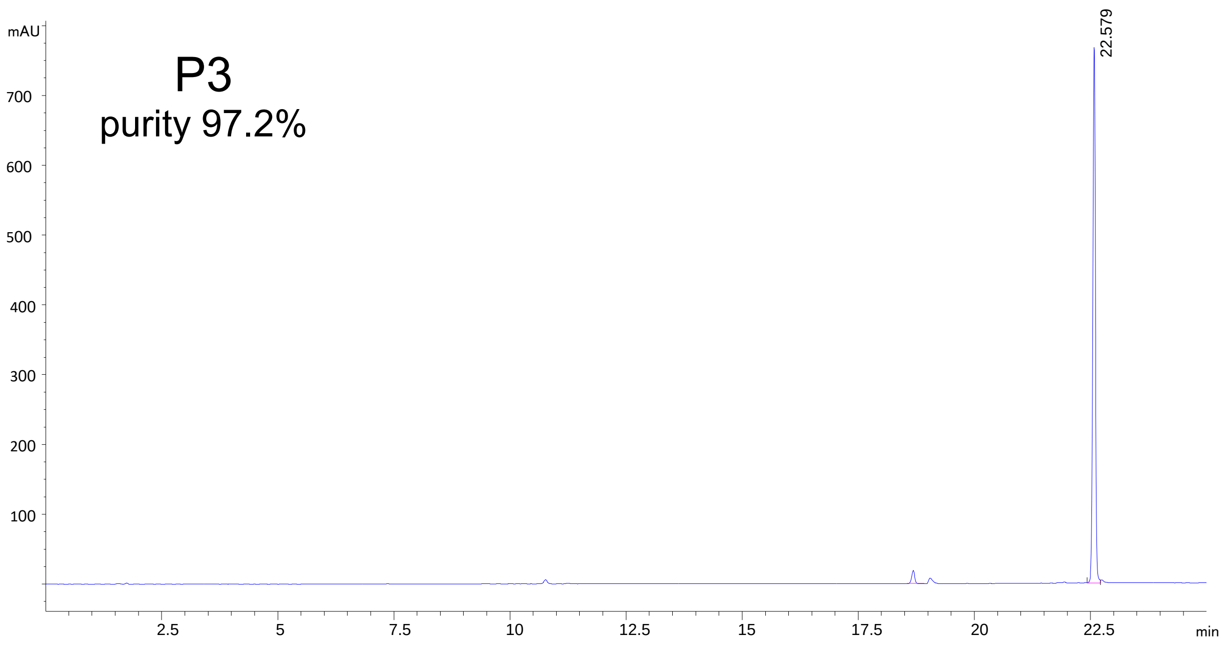

HPLC: (Methyl *o*-coumarate) mycophenolate (**P4**):

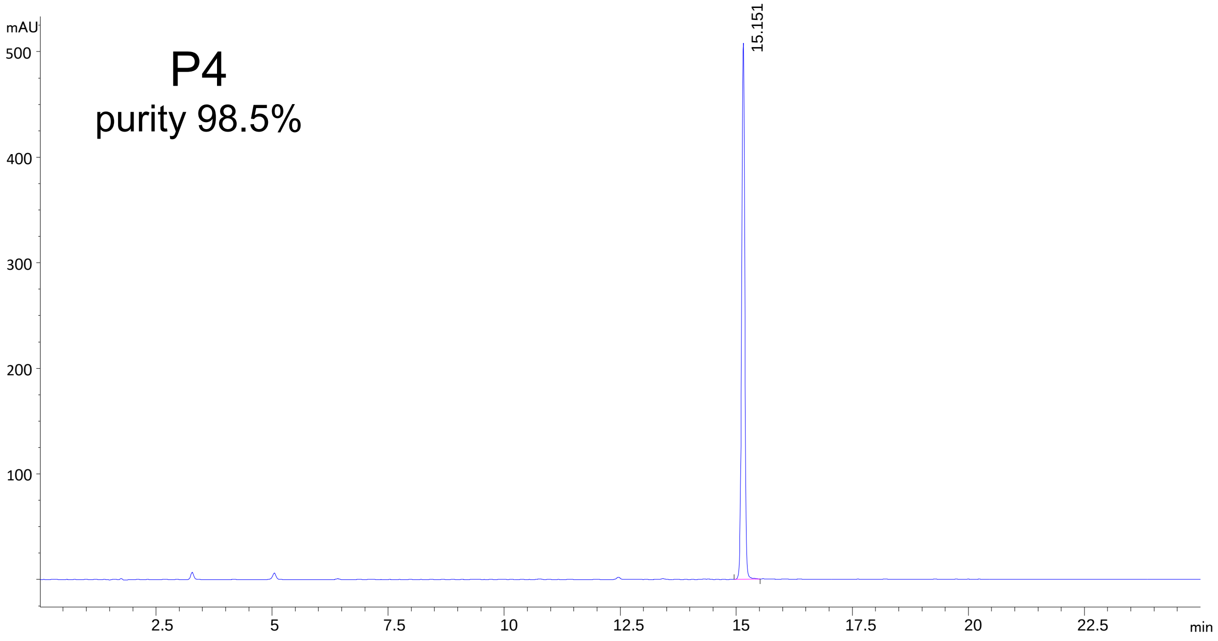

HPLC: 4-Hydroxybenzaldehyde mycophenolate (**P5**):

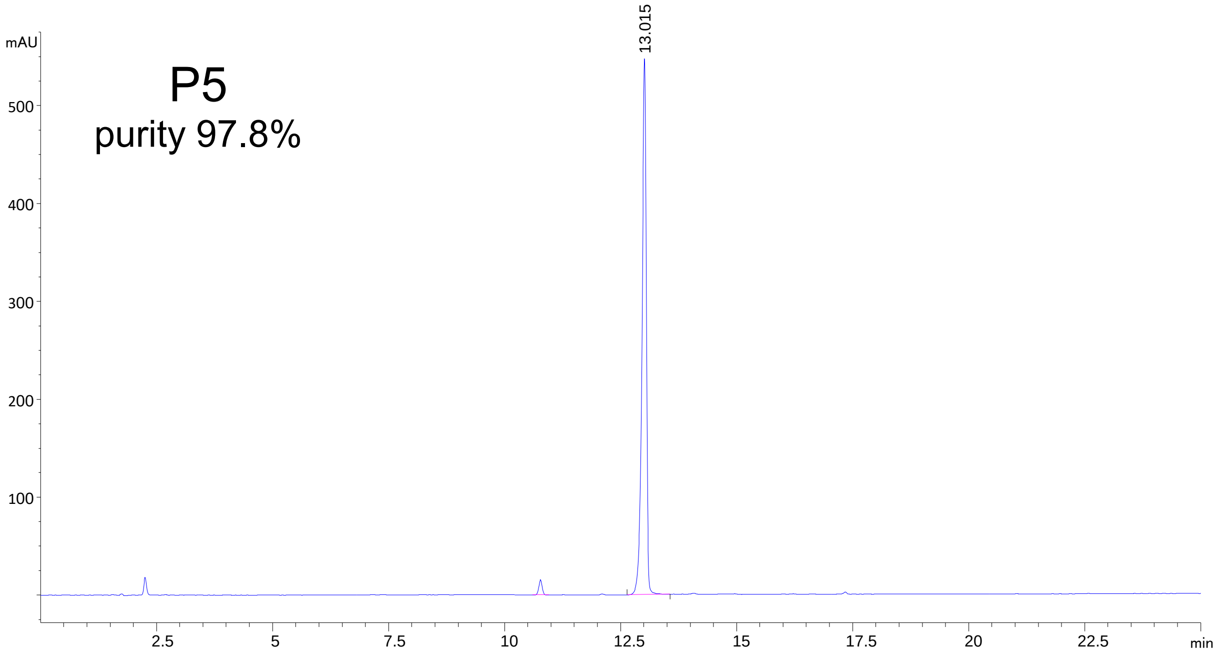

HPLC: Dehydroframbinone mycophenolate (**P6**):

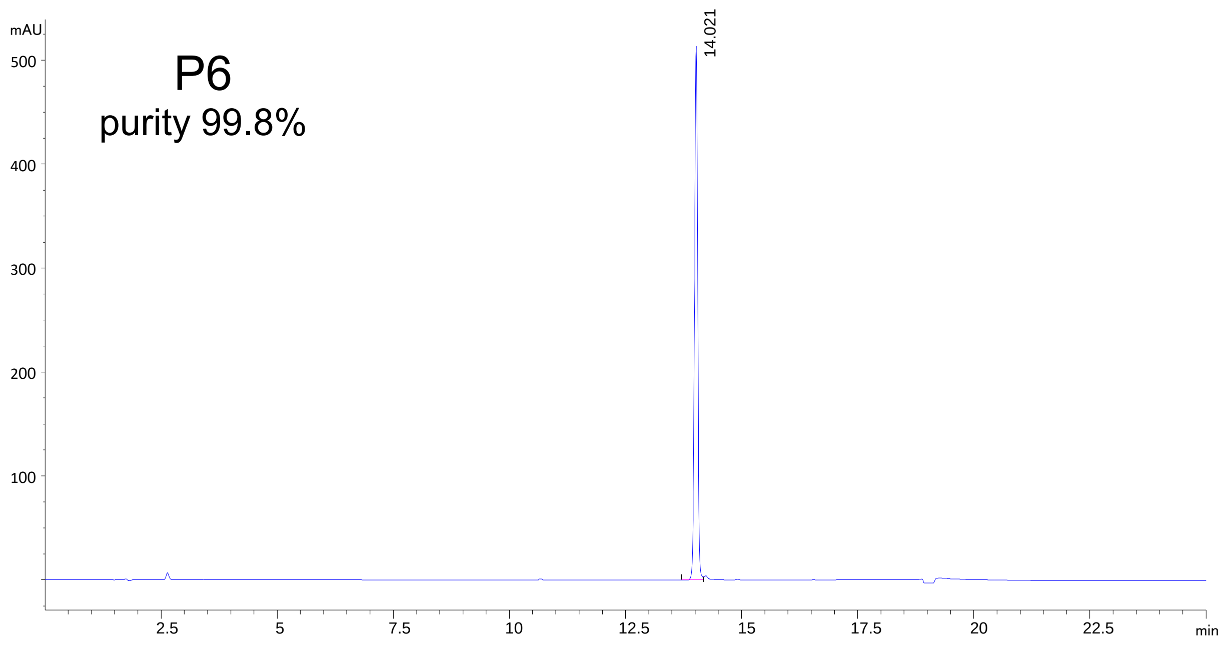

HPLC: Raspberry ketone mycophenolate (**P7**):

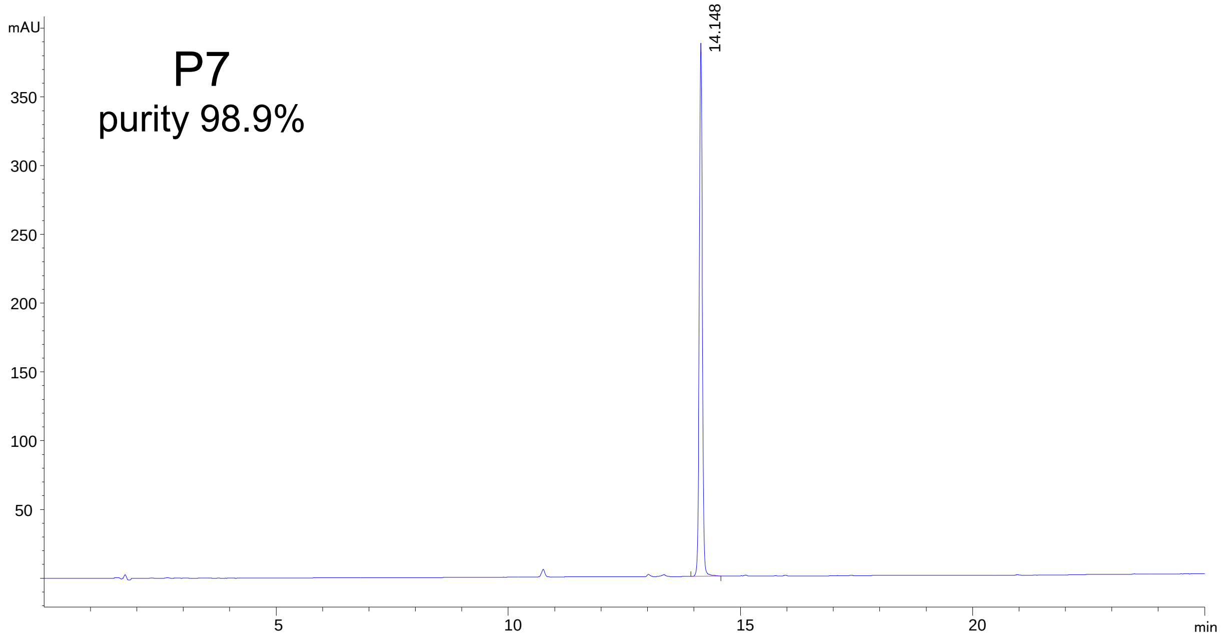

MS: Mycophenolic acid (**MPA**):

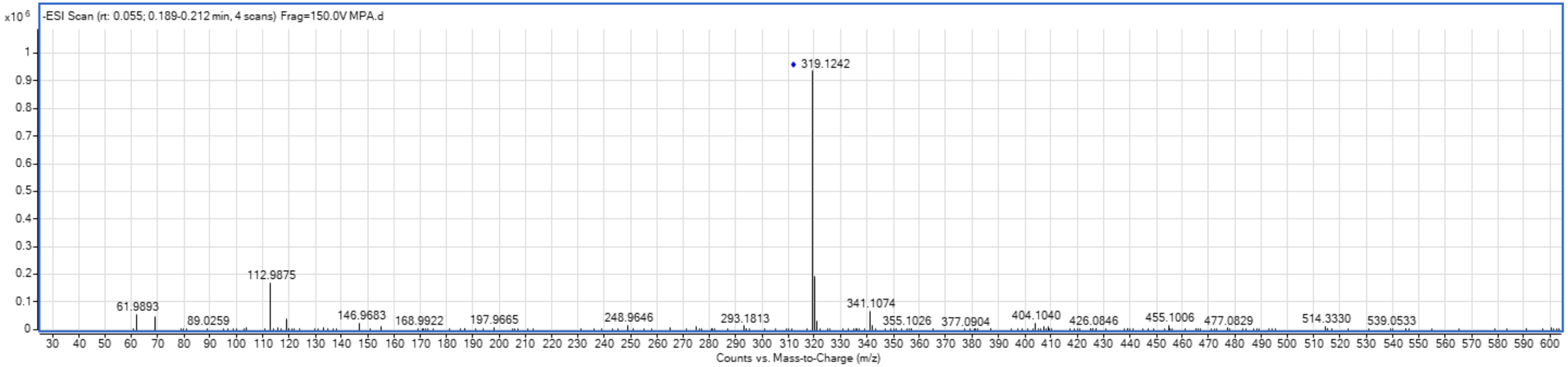

MS: Mycophenolate mofetil (**MMF**):

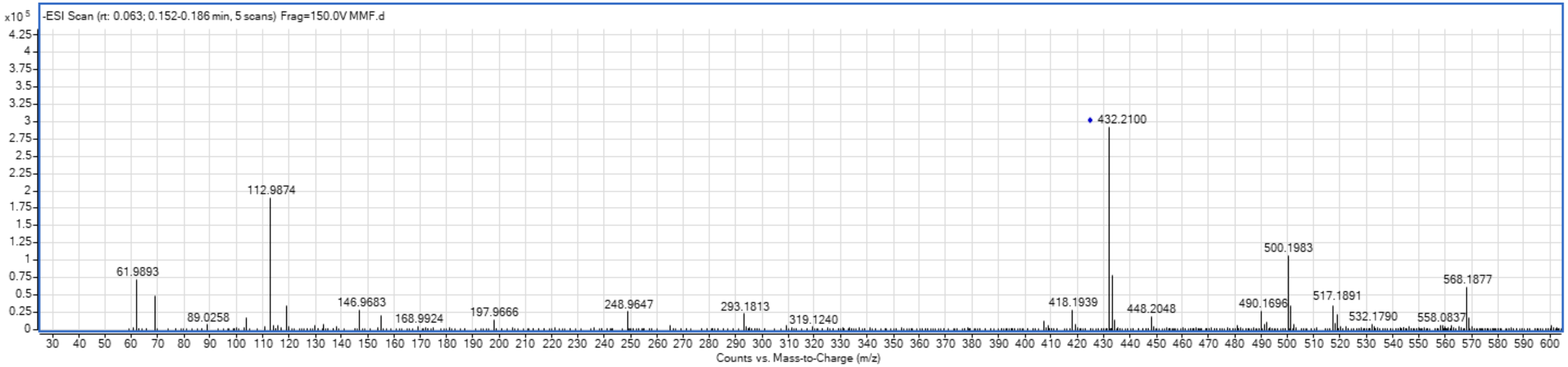

MS: Guaiacol mycophenolate (**MP1**):

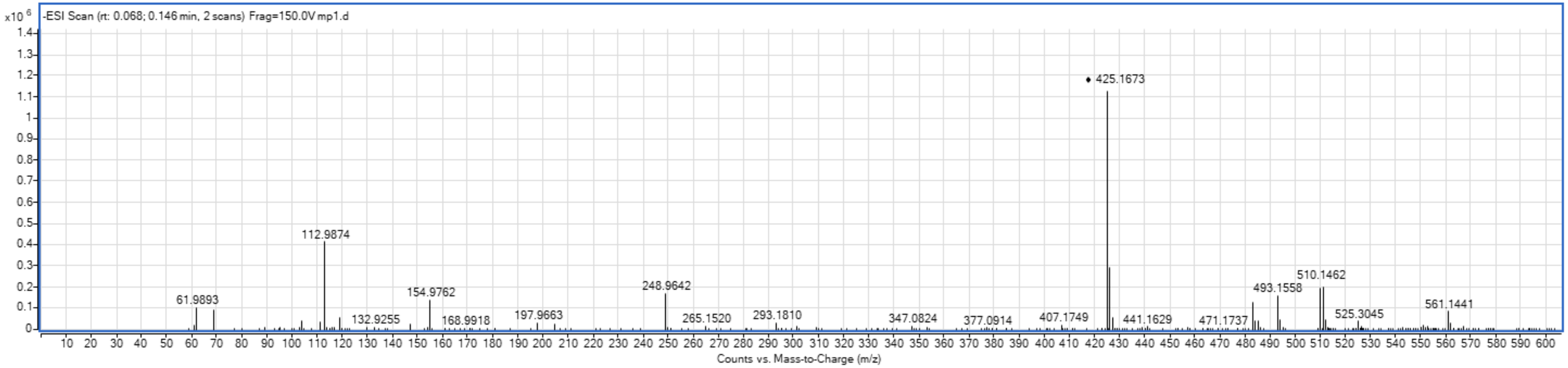

MS: Syringol mycophenolate (**MP2**):

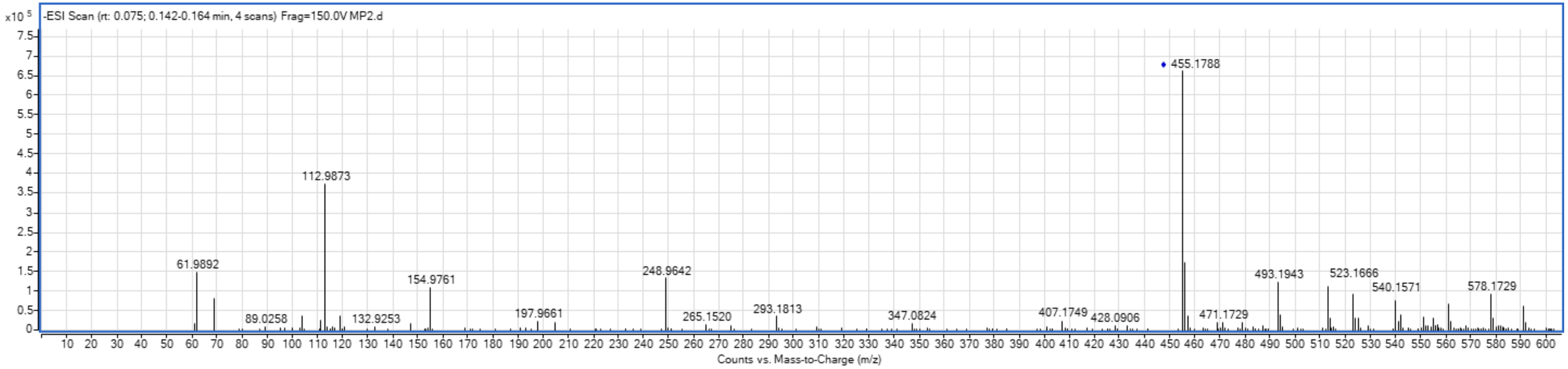

MS: Syringaldehyde mycophenolate (**MP3**):

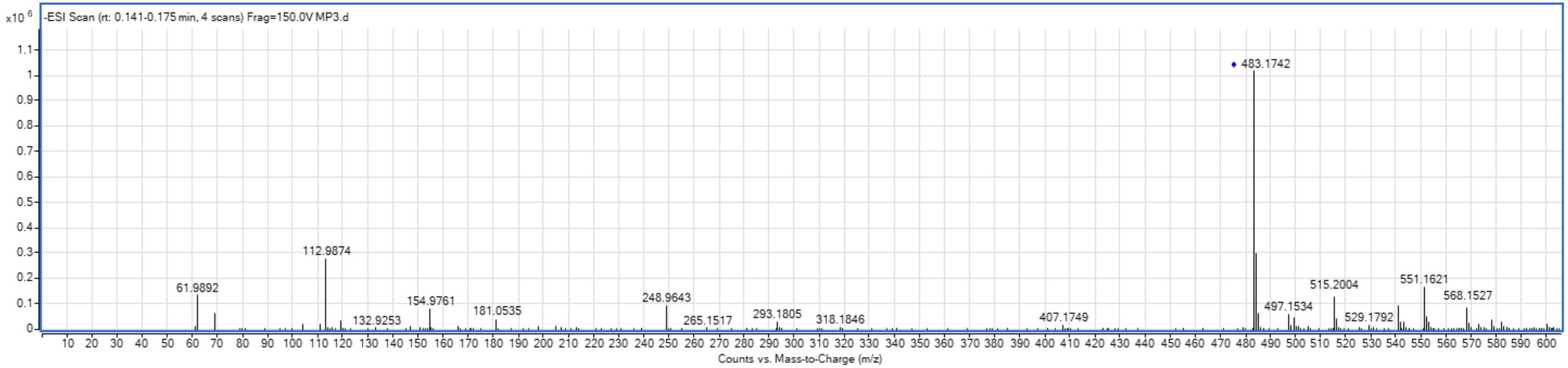

MS: Vanillin mycophenolate (**MP4**):

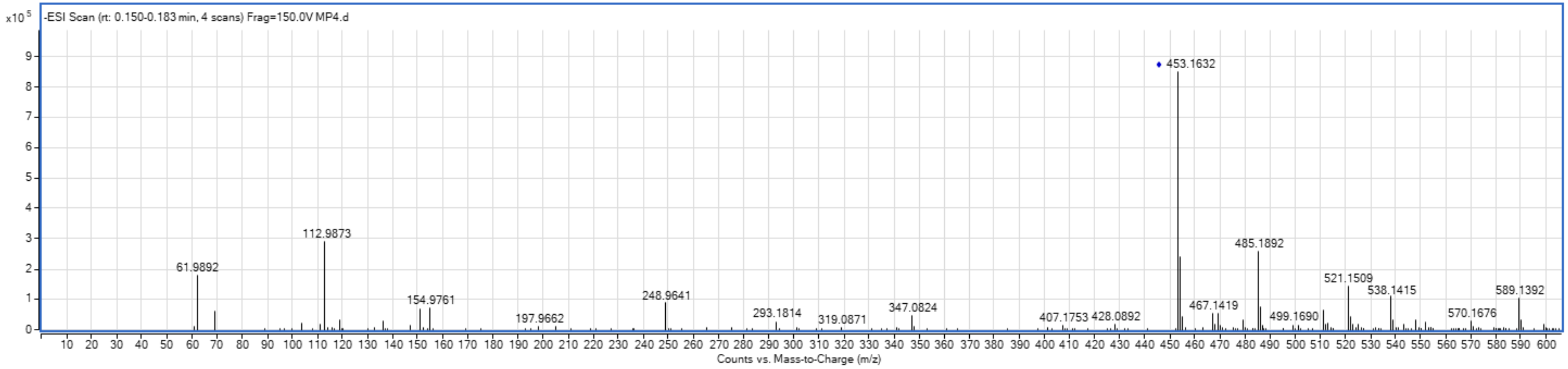

MS: Dehydrozingerone mycophenolate (**MP5**):

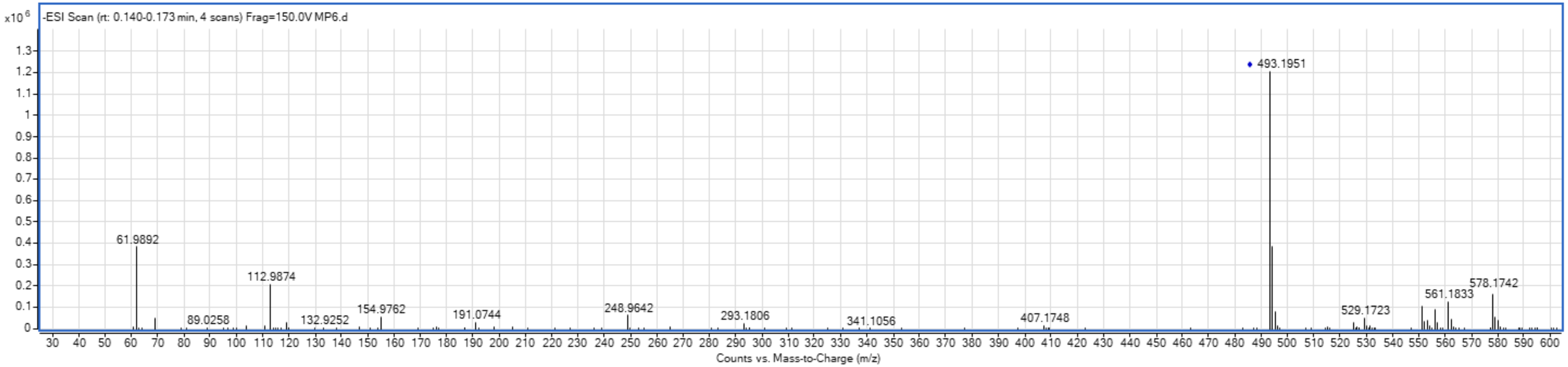

MS: Zingerone mycophenolate (**MP6**):

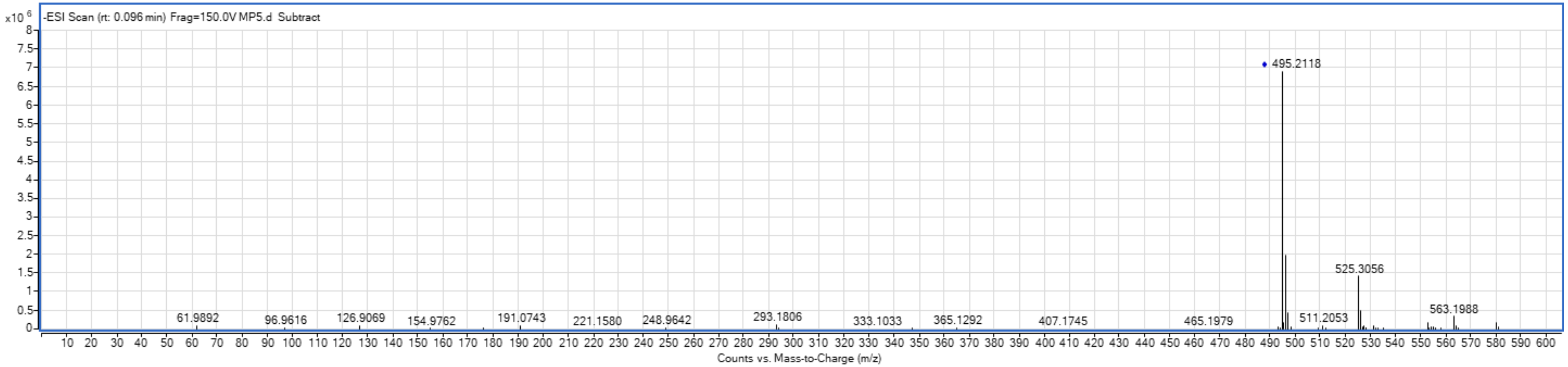

MS: (Methyl ferulate) mycophenolate (**MP7**):

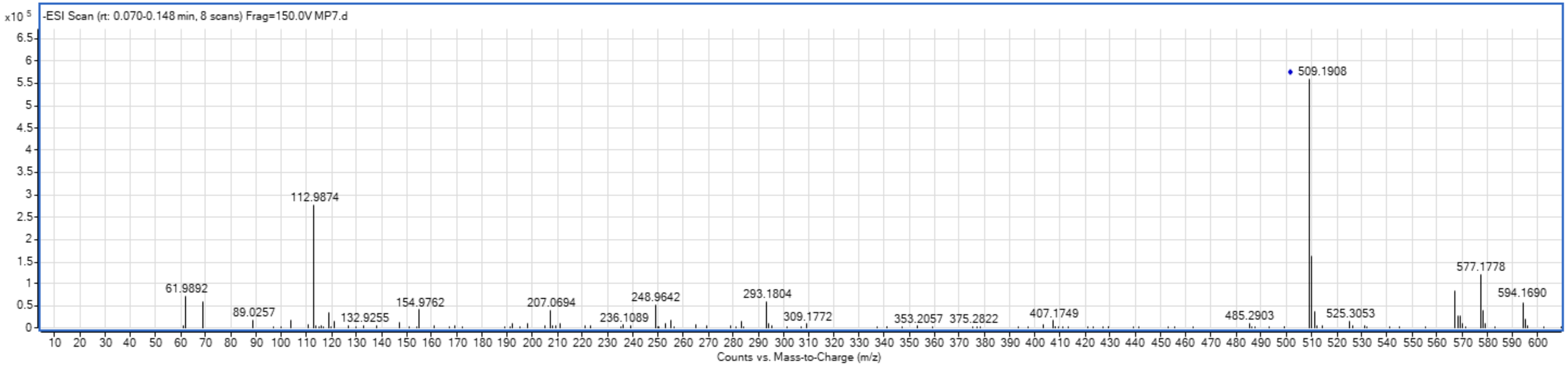

MS: Eugenol mycophenolate (**MP8**):

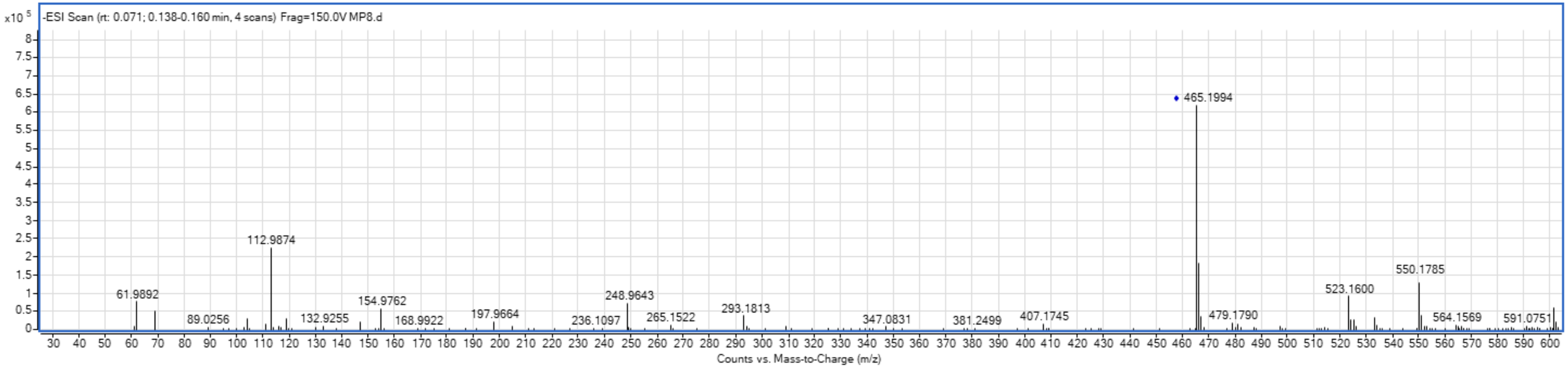

MS: Isoeugenol mycophenolate (**MP9**):

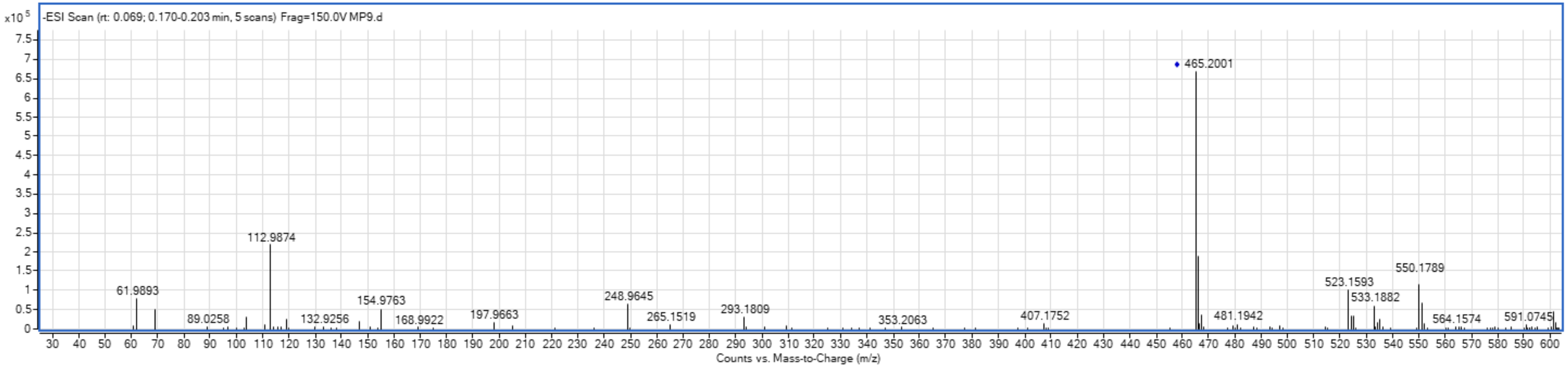

MS: Sesamol mycophenolate (**P1**):

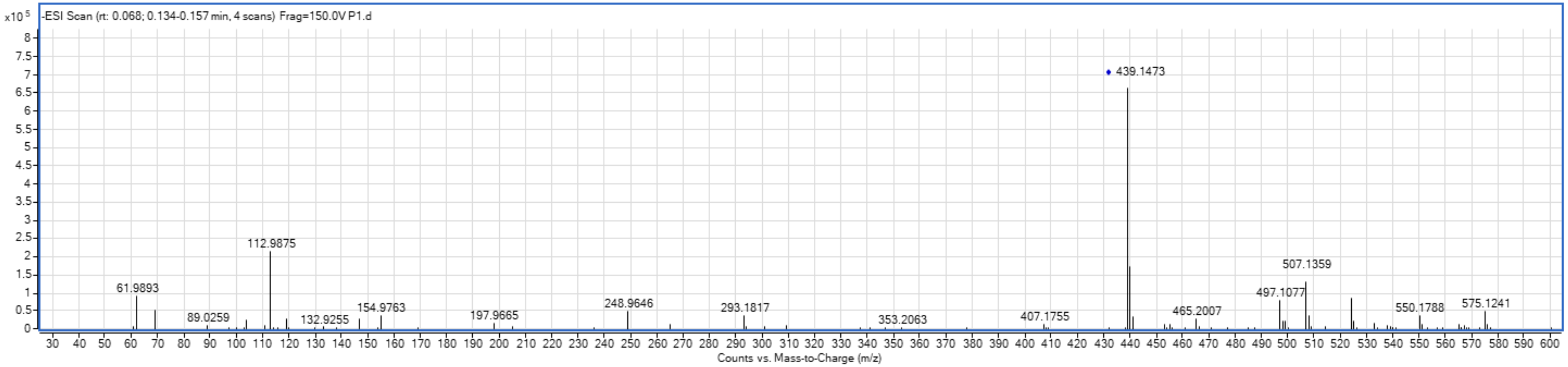

MS: Thymol mycophenolate (**P2**):

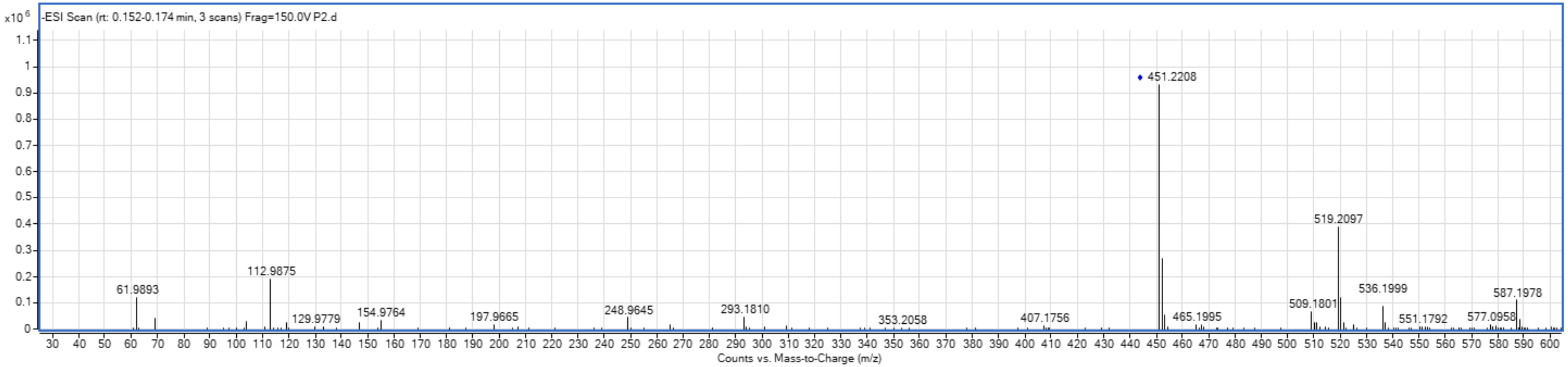

MS: Bakuchiol mycophenolate (**P3**):

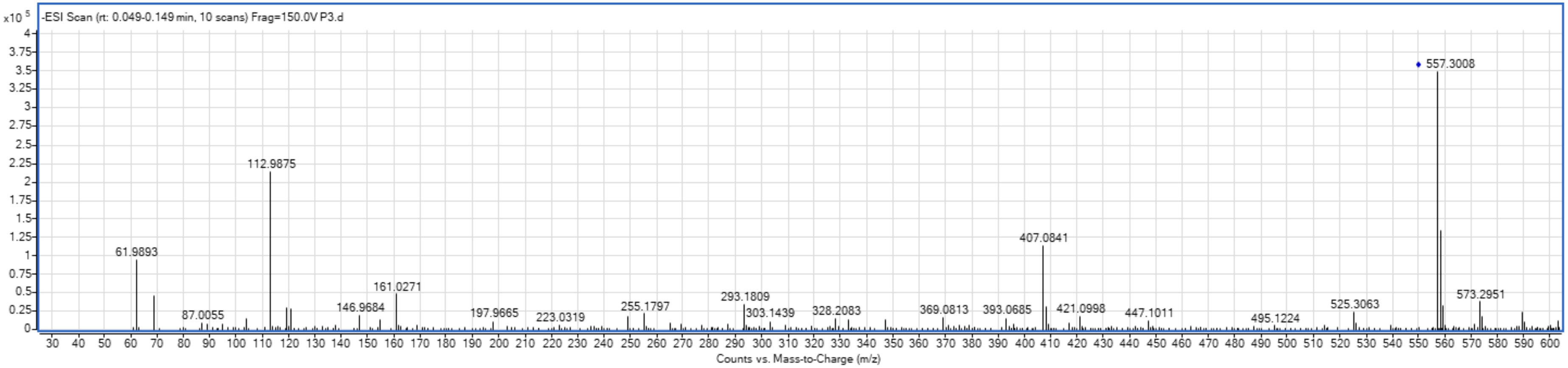

MS: (Methyl *o*-coumarate) mycophenolate (**P4**):

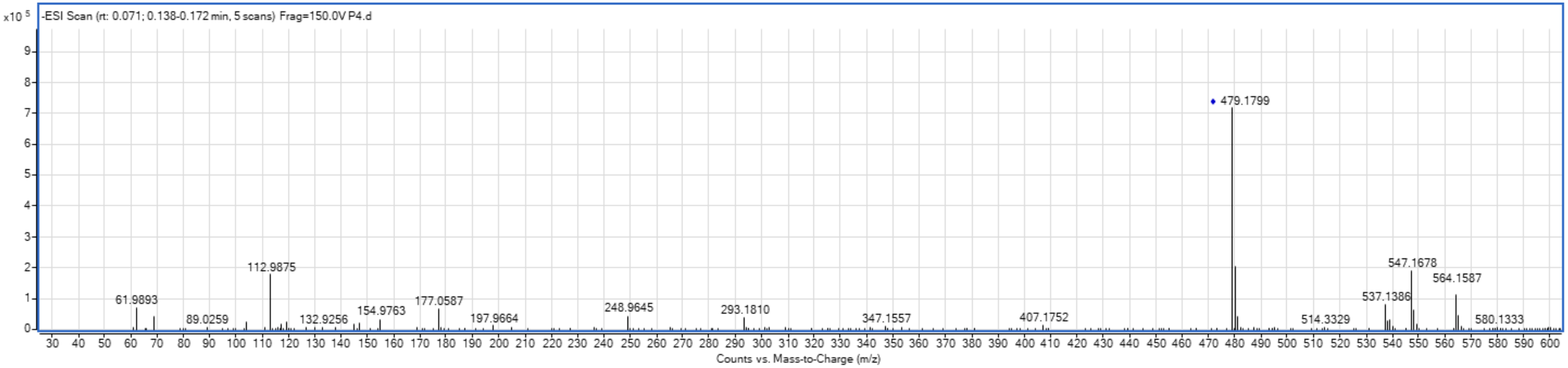

MS: 4-Hydroxybenzaldehyde mycophenolate (**P5**):

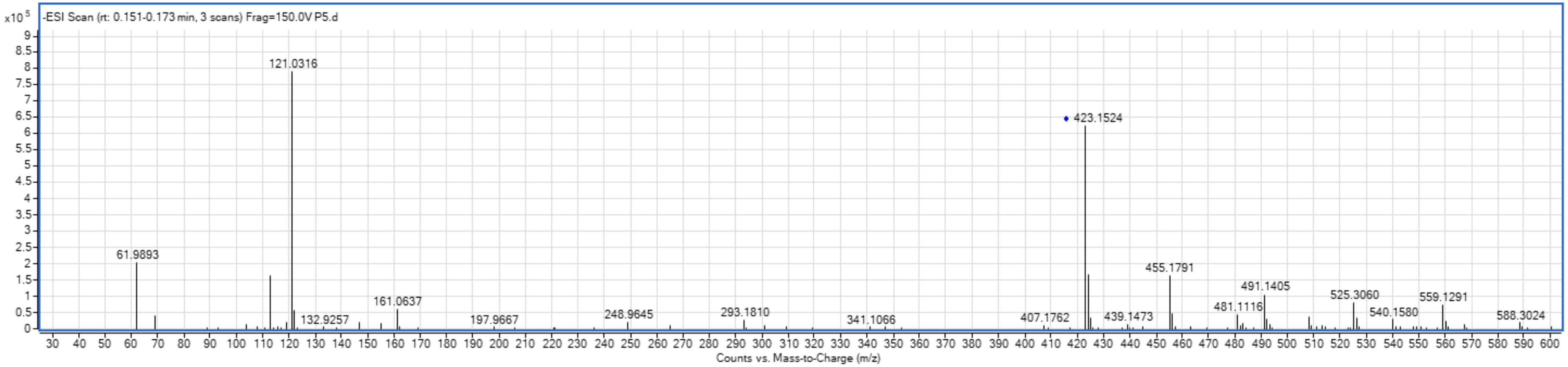

MS: Dehydroframbinone mycophenolate (**P6**):

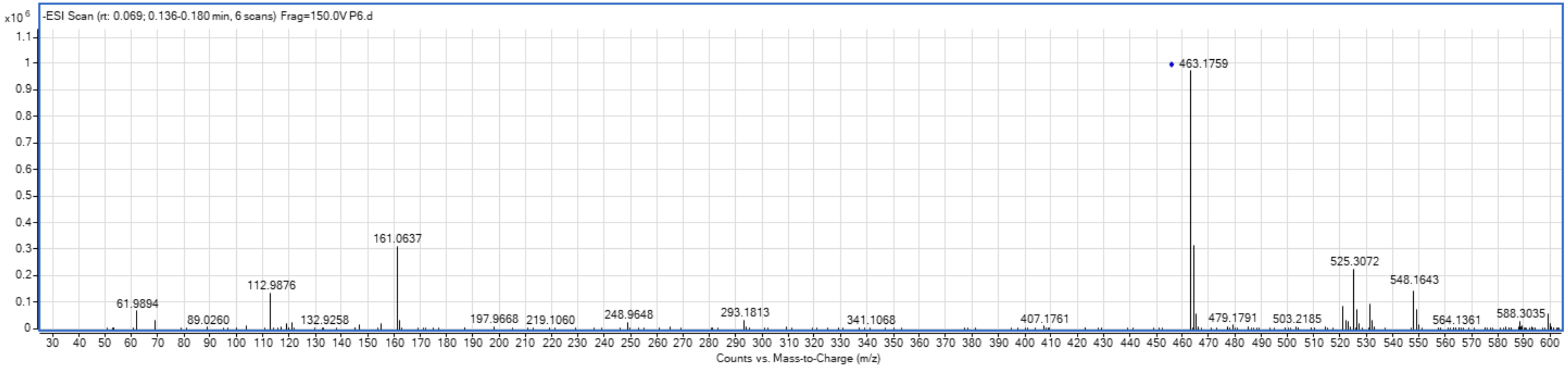

MS: Raspberry ketone mycophenolate (**P7**):

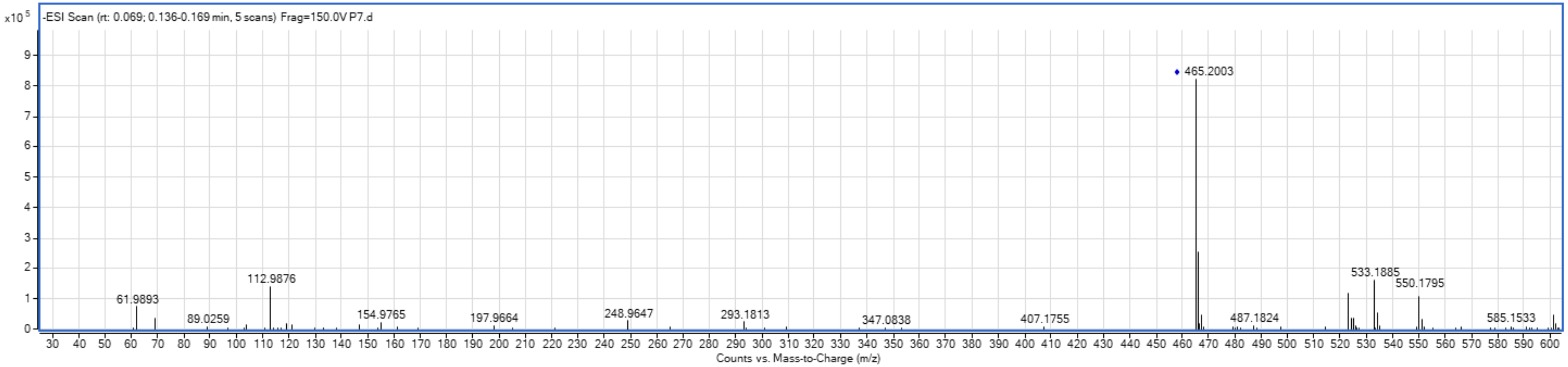

Remaining experiments (synthesis)

Synthesis of 7-*O*-sililated MPA

The same procedure was used as in [1].

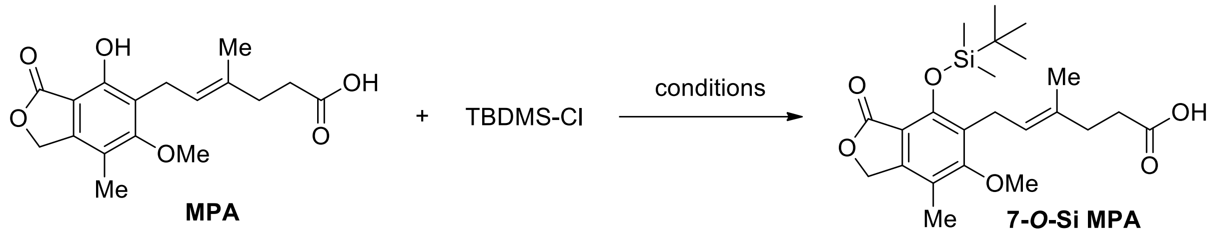

MPA (2 g, 6.243 mmol, 1.0 eq) was dissolved in dry DMF (10 mL), followed by the addition of tert-butyldimethylsilyl chloride (5.643 g, 37 mmol, 6.0 eq), imidazole (3.398 g, 50 mmol, 8.0 eq). The reaction mixture was stirred at room temperature for 1 hour. TLC indicated complete conversion of MPA. The mixture was then diluted with water (30 mL) and diethyl ether (60 mL). The organic layer was separated, washed with water (5 x 20 mL), dried over anhydrous $MgSO_4$, and filtered. The solvent was removed under reduced pressure to afford the crude product.

This crude intermediate was dissolved in THF (10 mL) and treated with acetic acid (10 mL) and water (10 mL). The resulting solution was stirred at room temperature, and the progress of the reaction was monitored by TLC. After 1 hour, no starting material was detected. Water (30 mL) and diethyl ether (60 mL) were added, and the organic phase was separated and washed five times with water (20 mL each). The combined organic extracts were dried over anhydrous $MgSO_4$, filtered, and concentrated under reduced pressure. The crude product was purified by column chromatography using a CH_2Cl_2:MeOH gradient (from 50:1 to 30:1 v/v), yielding a white solid with 77% yield (2.075 g, 4.8 mmol).

Synthesis of 7-*O*-sililated MPA ester with dehydrozingerone

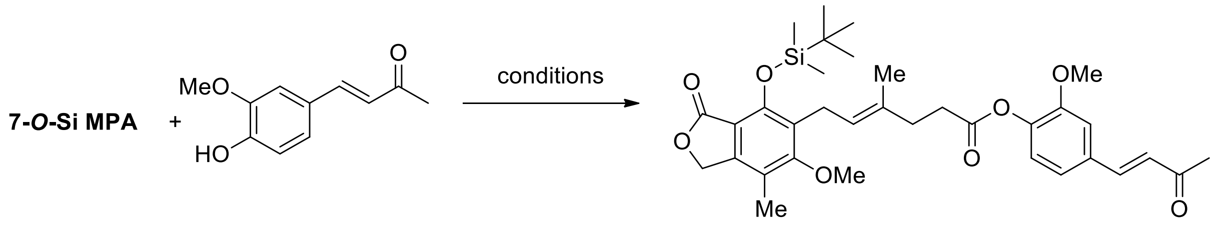

In a flask dried over a flame and flushed with a stream of argon, 100 mg of silylated MPA (0.230 mmol, 1.00 eq) and 47 mg of dehydrozingerone (0.242 mmol, 1.05 eq) were placed. The substrates were then dissolved in 2-3 mL of dry dichloromethane. After dissolving the reagents, the flask was flushed again with an inert gas stream. Subsequently, 83 mg of DMAP (0.023 mmol, 0.10 eq) was added. The mixture was cooled to 0°C, and 60 mg of DCC (0.276 mmol, 1.20 eq) was added in one portion. The system was stirred on a magnetic stirrer for at least 72 hours, allowing it to reach room temperature over time. Excessive evaporation of the volatile solvent can be prevented by placing the flask in a vessel filled with room-temperature water, which absorbs the heat generated by the magnetic stirring process.

The reaction mixture was then diluted with at least 3 mL of methylene chloride and successively extracted with saturated sodium bicarbonate solution, 1 M hydrochloric acid, and saturated brine. The organic phase was dried over anhydrous sodium or magnesium sulfate, filtered, and concentrated under reduced pressure. If the concentrated mixture contains a significant amount of fine crystalline urea byproduct, a small amount of methylene chloride is added to the system, cooled to 0°C, and filtered to discard the precipitate. The filtrate is then concentrated under reduced pressure.

The crude mixture was used in the next step after assessing its purity by TLC. The ester derivative was obtained as an off-white powder with a 70% yield (0.161 mmol, 98 mg).

Silyl protection removal (synthesis of dehydrozingerone mycophenolate, MP5)

The deprotection protocol was previously described[ESI1].

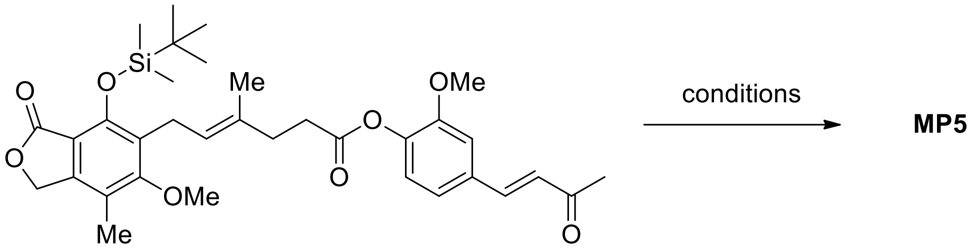

98 mg of 7-*O*-sililated MPA ester with dehydrozingerone (0.161 mmol) was dissolved in 3 mL of dry THF and subsequently treated with 135 μL of a 1 M solution of tetrabutylammonium fluoride in THF. The reaction mixture was stirred at room temperature for 15 minutes. It was then partitioned between water (10 mL) and ethyl acetate (10 mL). The organic phase was separated, dried over anhydrous $MgSO_4$, and filtered. The solvent was removed under reduced pressure. The crude product was purified by column chromatography using petroleum ether:ethyl acetate, 3:1 (v/v), affording 14 mg of MP5 in 17% yield, calculated with respect to silylated starting material.

Mukaiyama reaction procedure

The following conditions were developed based on the reference[ESI2].

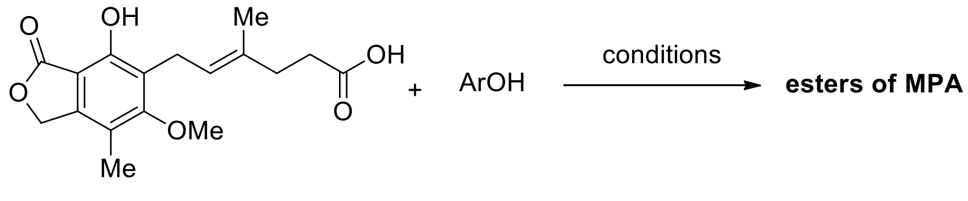

Mycophenolic acid (100 mg, 0.31 mmol, 1.0 eq) and the selected phenol (0.343 mmol, 1.1 eq) were dissolved in dry CH_2Cl_2 (3 mL) and cooled in an ice bath to 0 °C. 2-Chloro-N-methylpyridinium iodide, CMPI (160 mg, 0.624 mmol, 2.0 eq) and DMAP (76 mg, 0.624 mmol, 2.0 eq) were added. The ice bath was removed and the reaction mixture was stirred at room temperature for at least 72 hours. Reaction progress was monitored by TLC.

After completion, the reaction mixture was diluted with an equal volume of ethyl acetate (3 mL) and the resulting suspension was filtered. The filtrate was treated with 20% aqueous NH_4Cl (ca. 15 mL), and the layers were separated. The aqueous phase was extracted twice with ethyl acetate (2 × 10 mL). The combined organic layers were washed with 8% aqueous $NaHCO_3$ and brine, dried over anhydrous

Na_2SO_4, filtered, and concentrated under reduced pressure. The crude product was purified by column chromatography.

Yamaguchi reaction procedure for MP5 derivative

The synthesis of MP5 was adapted to current conditions in relation to the literature[ESI3].

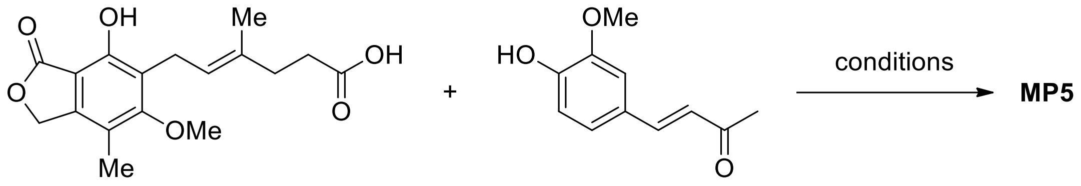

MPA (100 mg, 0.312 mmol), dehydrozingerone (60 mg, 0.312 mmol), DMAP (4 mg, 0.031 mmol), were dissolved in 2 mL of anhydrous chloroform. Then, benzoyl chloride (61 mg, 51 µL, 0.437 mmol), triethylamine (126 mg, 174 µL,1.249 mmol) were added simultaneously, and reaction mixture was stirred overnight at room temperature. The reaction progress was followed with TLC. No substrate consumption was spotted after 72 hours of continuous stirring.

ESI1. Cholewiński, G., Iwaszkiewicz-Grześ, D., Trzonkowski, P. & Dzierzbicka, K. Synthesis and biological activity of ester derivatives of mycophenolic acid and acridines/acridones as potential immunosuppressive agents. J. Enzyme Inhib. Med. Chem., **31**, 974–982 (2015).
ESI2. Hermann, T., Hochegger, P., Dolensky, J., Seebacher, W., Pferschy-Wenzig, E.-M., Saf, R., Kaiser, M., Mäser, P. & Weis, R. Synthesis and structure-activity relationships of new 2-phenoxybenzamides with antiplasmodial activity. Pharmaceuticals, **14**, 1109 (2021).
ESI3. Prejs, M., Cholewiński, G., Trzonkowski, P., Kot-Wasik, A. & Dzierzbicka, K. Synthesis and antiproliferative activity of new mycophenolic acid conjugates with adenosine derivatives. J. Asian Nat. Prod. Res., 1–8 (2018).

2D NMR — recorded for P7
HSQC

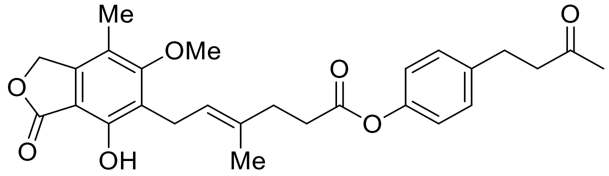

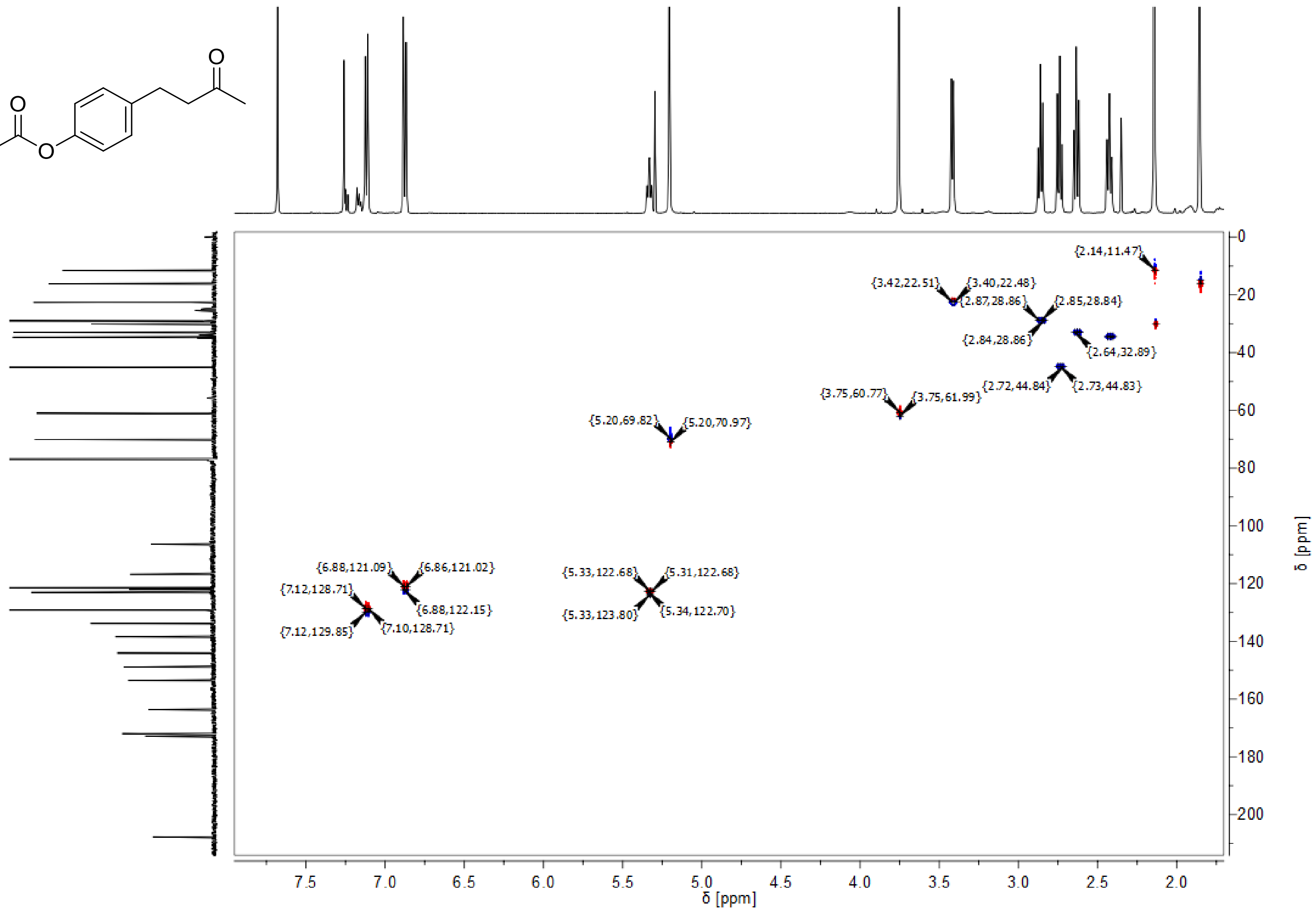

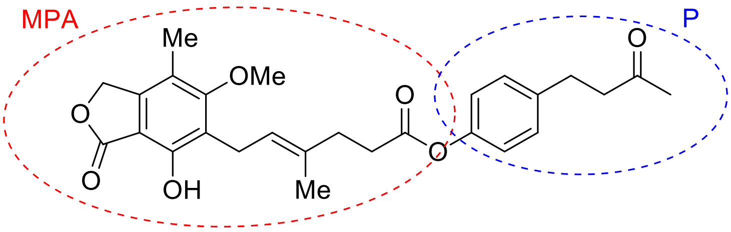

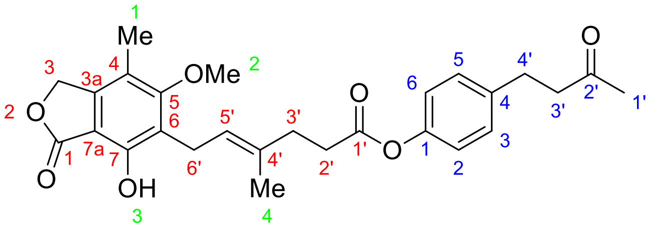

	HSQC		
	1D NMR		2D NMR
**C-H type**	**δ_H [ppm]**	**δ_C [ppm]**	**(δ_H; δ_C) [ppm]**
=C(-CH_3)- (MPA); 4	1.86	16.17	(1.85; 15.95)
-(C=O)-CH_3 (P); 1'	2.14	30.10	(2.13; 29.91)
Ar-CH_3 (MPA); 1	2.14	11.58	(2.14; 11.36)
=C(-Me)-CH_2- (MPA); 3'	2.43	34.62	(2.40; 34.43)<br>(2.42; 34.42)<br>(2.44; 34.42)
-CH_2-COO-Ar_P (MPA); 2'	2.63	33.04	(2.61; 32.83)<br>(2.63; 32.84)<br>(2.64; 32.83)
-CH_2-(C=O)- (P); 3'	2.74	45.05	(2.71; 44.84)<br>(2.73; 44.86)<br>(2.75; 44.86)
Ar_P-CH_2- (P); 4'	2.86	28.97	(2.84; 28.78)<br>(2.85; 28.78)<br>(2.87; 28.78)
Ar-CH_2- (MPA); 6'	3.42	22.61	(3.40; 22.40)<br>(3.42; 22.40)
Ar-OCH_3 (MPA); 2	3.75	61.00	(3.75; 60.83)
Ar-CH_2O- (MPA); 3	5.20	70.06	(5.20; 69.90)
>C=CH- (MPA); 5'	5.33	123.08	(5.31; 122.97)<br>(5.33; 122.96)<br>(5.34; 122.95)
$C_{Ar,P}$(2 and 6)-H; 2, 6	6.88	121.45	(6.86; 121.33)<br>(6.88; 121.32)
$C_{Ar,P}$(3 and 5)-H; 3, 5	7.12	129.16	(7.10; 129.05)<br>(7.12; 129.05)

The HSQC analysis results in proper carbon atoms assignment: 123.08 (><u>C</u>=C(-CH_3)-) and 122.02 (<u>C</u>$_{Ar}$-CH_2-CH=).

HMBC

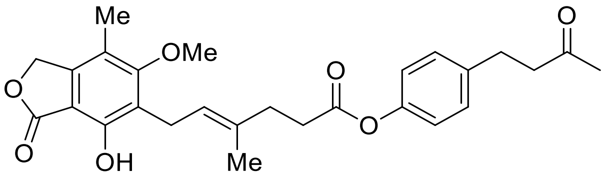
Me
OMe
O
O
O
O
O
OH
Me

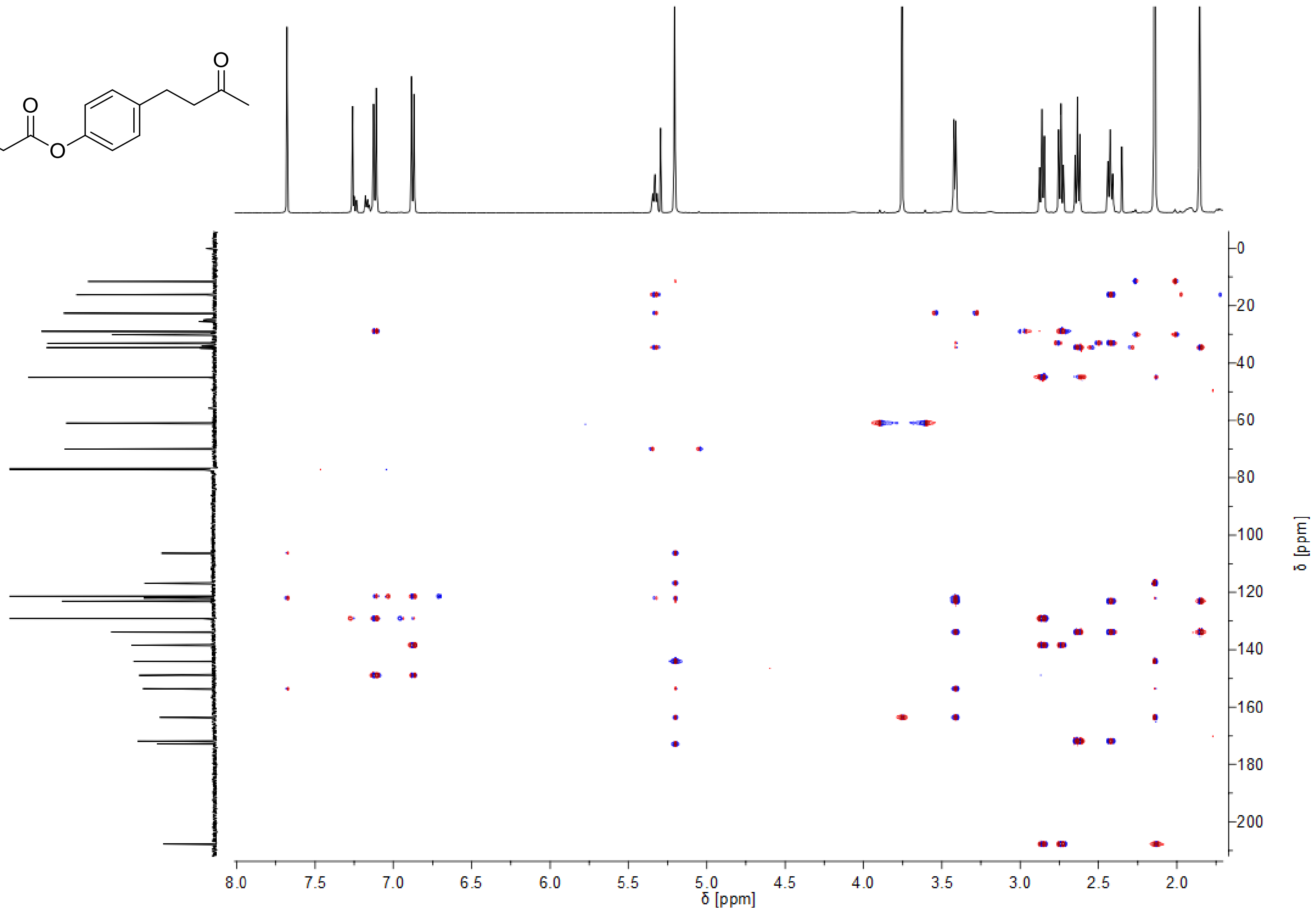
0
20
40
60
80
100
120
140
160
180
200
δ [ppm]
8.0
7.5
7.0
6.5
6.0
5.5
5.0
4.5
4.0
3.5
3.0
2.5
2.0
δ [ppm]

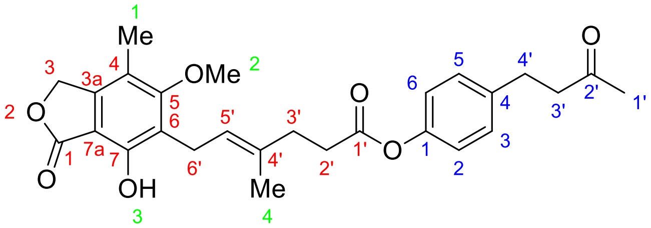

HMBC			
**Proton type**	**1D NMR δ$_H$ [ppm]**	**2D NMR (δ$_H$; δ$_C$) [ppm]**	**Comment**
4	1.86	(1.85; 34.54)	H4-C3’, 3 bonds
		(1.85; 123.03)	H4-C5’, 3 bonds
		(1.86; 133.78)	H4-C4’, 2 bonds
1’	2.14	(2.13; 207.76)	H1’-C2’, 2 bonds
1	2.14	(2.14; 116.72)	H1-C4, 2 bonds
		(2.13; 144.01)	H1-C3a, 3 bonds
		(2.14; 163.61)	H1-C5, 3 bonds
3’	2.43	(2.42; 16.08)	H3’-C4, 3 bonds
		(2.42; 32.95)	H3’-C2’, 2 bonds
		(2.42; 123.03)	H3’-C5’, 3 bonds
		(2.42; 133.79)	H3’-C4’, 2 bonds
		(2.42; 171.91)	H3’-C1’, 3 bonds
2’	2.63	(2.63; 34.54)	H2’-C3’, 2 bonds
		(2.63; 133.78)	H2’-C4’, 3 bonds
		(2.64; 171.90)	H2’-C1’, 2 bonds
3’	2.74	(2.74; 28.86)	H3’-C4’, 2 bonds
		(2.74; 138.41)	H3’-C4, 3 bonds
		(2.74; 207.75)	H3’-C2’, 2 bonds
4’	2.86	(2.86; 44.98)	H4’-C3’, 2 bonds
		(2.86; 129.11)	H4’-C3, 3 bonds<br>H4’-C5, 3 bonds
		(2.86; 138.38)	H4’-C4, 2 bonds
		(2.86; 207.75)	H4’-C2’, 3 bonds
6’	3.42	(3.41; 122.02)	H6’-C6, 2 bonds
		(3.41; 133.79)	H6’-C4’, 3 bonds
		(3.41; 153.55)	H6’-C7, 3 bonds
		(3.41; 163.62)	H6’-C5, 3 bonds
2	3.75	(3.75; 163.62)	H2-C5, 3 bonds
3	5.20	(5.20; 11.43)	H3-C1, 4 bonds
		(5.20; 106.29)	H3-C7a, 3 bonds
		(5.20; 116.73)	H3-C4, 3 bonds
		(5.20; 121.99)	H3-C6, 5 bonds
		(5.20; 144.00)	H3-C3a, 2 bonds
		(5.20; 153.53)	H3-C7, 4 bonds
		(5.20; 163.62)	H3-C5, 4 bonds
		(5.20; 172.89)	H3-C1, 3 bonds
5’	5.33	(5.32; 16.08)	H5’-C4, 3 bonds
		(5.34; 22.53)	H5’-C6’, 2 bonds
		(5.32; 34.53)	H5’-C3’, 3 bonds
		(5.32; 121.99)	H5’-C6, 3 bonds

HMBC			
**Proton type**	**1D NMR δ_H [ppm]**	**2D NMR (δ_H; δ_C) [ppm]**	**Comment**
2, 6	6.88	(6.88; 121.43)	H2-C6, 3 bonds<br>H6-C2, 3 bonds
		(6.88; 138.41)	H2-C4, 3 bonds<br>H6-C4, 3 bonds
		(6.88; 148.88)	H2-C1, 2 bonds<br>H6-C1, 2 bonds
3, 5	7.12	(7.12; 28.89)	H3-C4', 3 bonds<br>H5-C4', 3 bonds
		(7.12; 129.12)	H3-C5, 3 bonds<br>H5-C3, 3 bonds
		(7.12; 148.88)	H3-C1, 3 bonds<br>H5-C1, 3 bonds
3	7.68	(7.68; 106.27)	H3-C7a, 3 bonds
		(7.68; 121.91)	H3-C6, 3 bonds
		(7.68; 153.57)	H3-C7, 2 bonds

The HMBC analysis results in proper proton and carbon assignments (in 1D and HSQC measurements). (a) 172.90 (-C̲OO-) ⇔ 171.91 (-C̲OO-Ar_P) — on the basis of 3, 3', and 2' correlations. (b) 123.08 (>C̲=C(-CH_3)-) ⇔ 122.02 ($\underline{C}_{Ar}$-CH_2-CH=) — based on 3' and 3. (c) 34.62 (-C̲H_2-CH_2-) ⇔ 33.04 (-C̲H_2-COO-Ar_P) — in relation to 2', 3', and 5' interplay.

NOESY

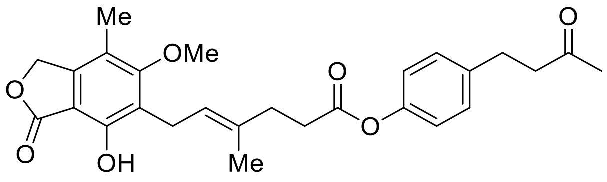
Me
OMe
O
O
O
OH
Me
O
O
O

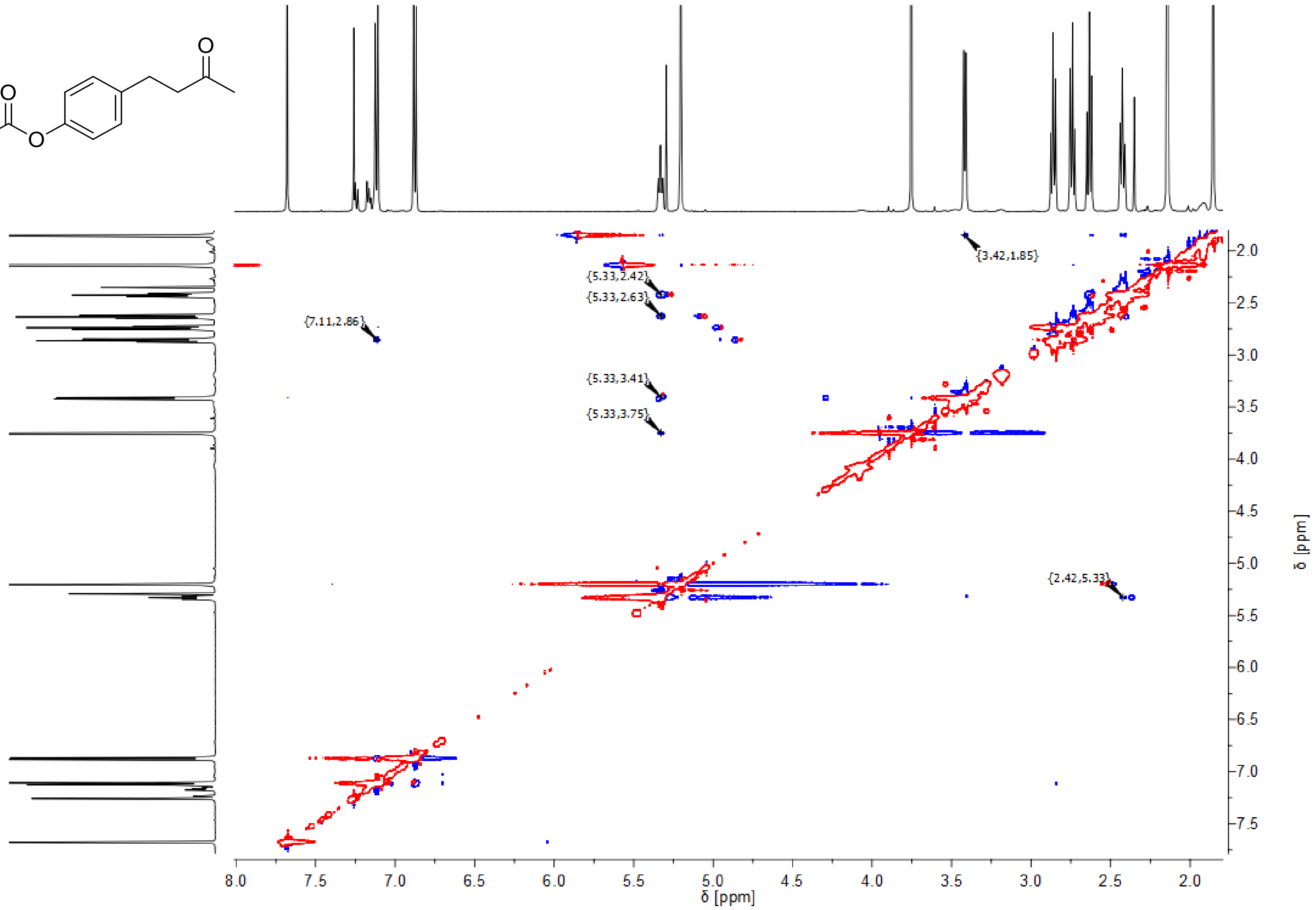
{3.42,1.85}
{5.33,2.42}
{5.33,2.63}
{7.11,2.86}
{5.33,3.41}
{5.33,3.75}
{2.42,5.33}
8.0
7.5
7.0
6.5
6.0
5.5
5.0
4.5
4.0
3.5
3.0
2.5
2.0
δ [ppm]
2.0
2.5
3.0
3.5
4.0
4.5
5.0
5.5
6.0
6.5
7.0
7.5
δ [ppm]

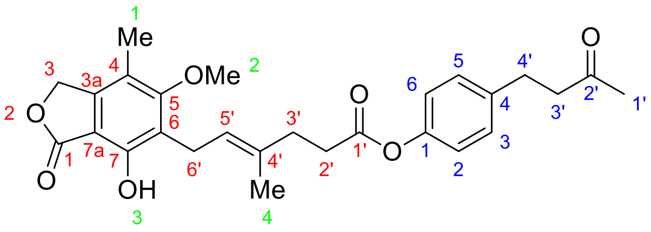

NOESY	
2D NMR (δ_H; δ_H) [ppm]	Comment
(1.85; 3.42)	H4-H6'
(2.42; 5.33)	H3'-H5'
(2.63; 5.33)	H2'-H5'
(2.86; 7.11)	H4'-H(3,5)
(3.41; 5.33)	H6'-H5'
(3.75; 5.33)	H2-H5'
(5.33; 2.42)	H5'-H3'
